# A trophic latitudinal gradient revealed in anchovy and sardine from the Western Mediterranean Sea using a multi-proxy approach

**DOI:** 10.1038/s41598-020-74602-y

**Published:** 2020-10-19

**Authors:** Eneko Bachiller, Marta Albo-Puigserver, Joan Giménez, Maria Grazia Pennino, Neus Marí-Mena, Antonio Esteban, Elena Lloret-Lloret, Angelique Jadaud, Belén Carro, José María Bellido, Marta Coll

**Affiliations:** 1 Institut de Ciències del Mar (ICM-CSIC), Passeig Marítim de la Barceloneta, 37-49, 08003 Barcelona, Spain; 2 Instituto Español de Oceanografía, Centro Oceanográfico de Vigo, Subida Radio Faro, 50, 36390 Vigo, Spain; 3 AllGenetics and Biology SL, Edificio CICA, Campus de Elviña, 15008 A Coruña, Spain; 4 Instituto Español de Oceanografía, Centro Oceanográfico de Murcia, Varadero 1 Apdo 22, 30740 San Pedro del Pinatar, Murcia, Spain; 5 Marine Biodiversity, Exploitation and Conservation (MARBEC), Ifremer, University Montpellier, CNRS, IRD, Sète, France; 6 Present Address: AZTI, Sustainable Fisheries Management (Data), Basque Research and Technology Alliance (BRTA), Txatxarramendi uhartea z/g, 48395 Sukarrieta, Bizkaia (Basque Country) Spain

**Keywords:** Food webs, Marine biology

## Abstract

This work combines state-of-the-art methods (DNA metabarcoding) with classic approaches (visual stomach content characterization and stable isotope analyses of nitrogen (*δ*^15^N) and carbon (*δ*^13^C)) to investigate the trophic ecology of anchovy (*Engraulis encrasicolus*) and sardine (*Sardina pilchardus*) at high taxonomic and spatial resolution in the Western Mediterranean Sea. Gut contents observed are in accordance with the dietary plasticity generally described for anchovy and sardine, suggesting a diet related to the opportunistic ingestion of available prey in a certain area and/or time. Genetic tools also showed modest inter-specific differences regarding ingested species. However, inter-specific and intra-specific differences in ingested prey frequencies and prey biomass reflected a latitudinal signal that could indicate a more effective predation on large prey like krill by anchovy versus sardine, as well as a generalized higher large prey ingestion by both species southwards. In fact, both species presented lower *δ*^15^N in the northernmost area. This latitudinal gradient indicates changes in the trophic ecology of anchovy and sardine that coincide with previously described better biological conditions for fish in the southern part of the study area as well as higher landings of both species in recent years.

## Introduction

European sardine (*Sardina pilchardus*) and European anchovy (*Engraulis encrasicolus*) are two of the most exploited small pelagic fish in the Mediterranean Sea, representing *ca.* 50% of the total Mediterranean fish landings^1^. In addition to their commercial importance, their high abundance and pivotal trophic position in the marine food web highlights their relevance for the Mediterranean ecosystem^2–5^.

During the last decade, declines in stock biomass of these species have concerned the fishing industry^2,6^. Historical observations over the past two decades in the Western Mediterranean Sea have shown decreases in stock biomass (and landings) for sardine in the north and fluctuations in the southern part^7–10^, whereas anchovy biomass has decreased or fluctuated in the north and increased in the south^7,8,10^. In addition, changes in body condition, growth, size at first maturity and disappearance of older ages have been observed for both species, also shows a latitudinal trend with higher incidence of change in the northern versus the southern part of the Western Mediterranean Sea^7–9,11^.

In this sense, understanding ecological processes affecting fluctuations of such fisheries stocks has been a key issue in recent studies^2,12^. In fact, these species are planktivorous consuming a wide range of prey during their life cycle^13–19^. Recent studies have suggested a relatively high niche overlap since 2010 and a reduction in prey diversity in the northern part of the Western Mediterranean^20^, that might have caused fluctuations in stocks partially affected by inter-specific feeding interactions. However, most studies have been focused on the northwesternmost Mediterranean area, the Gulf of Lion^20^, whereas the ecological status and trophic ecology of small pelagic fish in the whole Western Mediterranean Sea remains uncertain^21^.

Recently, DNA metabarcoding has emerged as a useful technique to study the trophic ecology of several organisms^22–24^. However, these techniques are not so often combined with other methods to enhance their utility. In fact, methods like the high-throughput DNA sequencing (HTS) of diets^25–27^ can be useful to parameterize food webs at enhanced taxonomic resolution, potentially improving the parametrization of ecosystem models, although they cannot quantify the amount of ingested prey^28^.

The direct quantification of ingested prey can be achieved by analysing gut contents under the microscope, which is time-consuming and requires high taxonomic expertise^29^ or through mass-balanced stable isotope mixing models^30^, which require a proper isotopic reference collection of potential food sources and normally gives poor taxonomic resolution^31,32^. Furthermore, gut content analysis by visual inspection and DNA metabarcoding only provides a snapshot of what the fish has ingested in the last few hours^29,33^, which can lead to some bias if we are interested in the long term trophic ecology of the species. In addition, the gut content characterization technique also presents some limitations, as for example some gelatinous species and certain fish eggs that are difficult to detect due to their high vulnerability to the digestion processes^34^.

Complementary, stable isotope analysis provides information on the assimilated diet rather than the ingested one over a longer time period^35^. In the absence of potential prey stable isotope signatures (imperative to estimate the assimilated diet), they still provide useful information about trophic position, isotopic width and overlap, as a proxy of trophic niche width and overlap between species^36,37^. Thus, the combination of different techniques can provide new insights and show a general overview of the feeding ecology of organisms^22^ (Table 1).

**Table 1:** Comparative methodological framework applied in this study to characterize the trophic ecology of anchovy (*Engraulis encrasicolus*) and sardine (*Sardina pilchardus*) in the Western Mediterranean Sea, using four different aspects (1–4), the corresponding comparable metrics and three sources of information (A–C).

Variable	A. Gut content characterization	B. Stable Isotope Analysis	C. DNA metabarcoding
1. Diet characterization	Feeding Intensity (SFD) Numerical frequency (%N) Prey biomass (%B) Frequency of occurrence (%FO)	*δ*^13^C and * δ*^15^N	%FO mOTUs/OTUs
2. Prey diversity/niche width	Species Richness Rarefaction curves (species richness) Shannon–Wiener diversity index	Ellipses width (SEA_B_)	Species Richness Rarefaction curves (species richness)
3. Diet similarity	Beta-diversity	–	Beta-diversity
4. Niche overlap	Pianka Niche Overlap	Ellipses area overlap (SEA_B_)	Pianka Niche Overlap

In this study, we characterize the trophic ecology of sardine and anchovy in the Western Mediterranean Sea, and explore potential geographical (i.e. latitudinal) variations in their diet, combining the three different methodologies mentioned above: microscope analysis and DNA metabarcoding of gut contents, and stable isotope analysis of muscle samples. In addition, we perform generalized additive models (GAMs) to test the influence of latitude, area, bathymetry, total length of fish on gut content indices and stable isotope variables for both species.

Our hypothesis is that due to an uneven stock status and population dynamics of the two species during the last decade, latitudinal changes in trophic ecology may partially explain apparent fluctuations in stocks. Previous studies have shown that the genetic structure of both species is similar in the Western Mediterranean Sea^38^ except around south-western waters (Alboran Sea), where a transition area between the Mediterranean Sea and the Atlantic Ocean populations has been established^9,38^. Therefore, plausible causes of ecological change need to be investigated.

The present study represents the first approach that combines individual visual diet characterization of small pelagic fish with metabarcoding and stable isotope analyses in the Mediterranean marine ecosystem. This new information is essential to advance on our understanding of the ecological status and processes affecting fluctuations and change in population dynamics of anchovy and sardine in the study area. It also provides useful information about their feeding dynamics for future studies addressing potential trophic interactions between pelagic species sharing the ecosystem.

## Results

**Figure 1:**
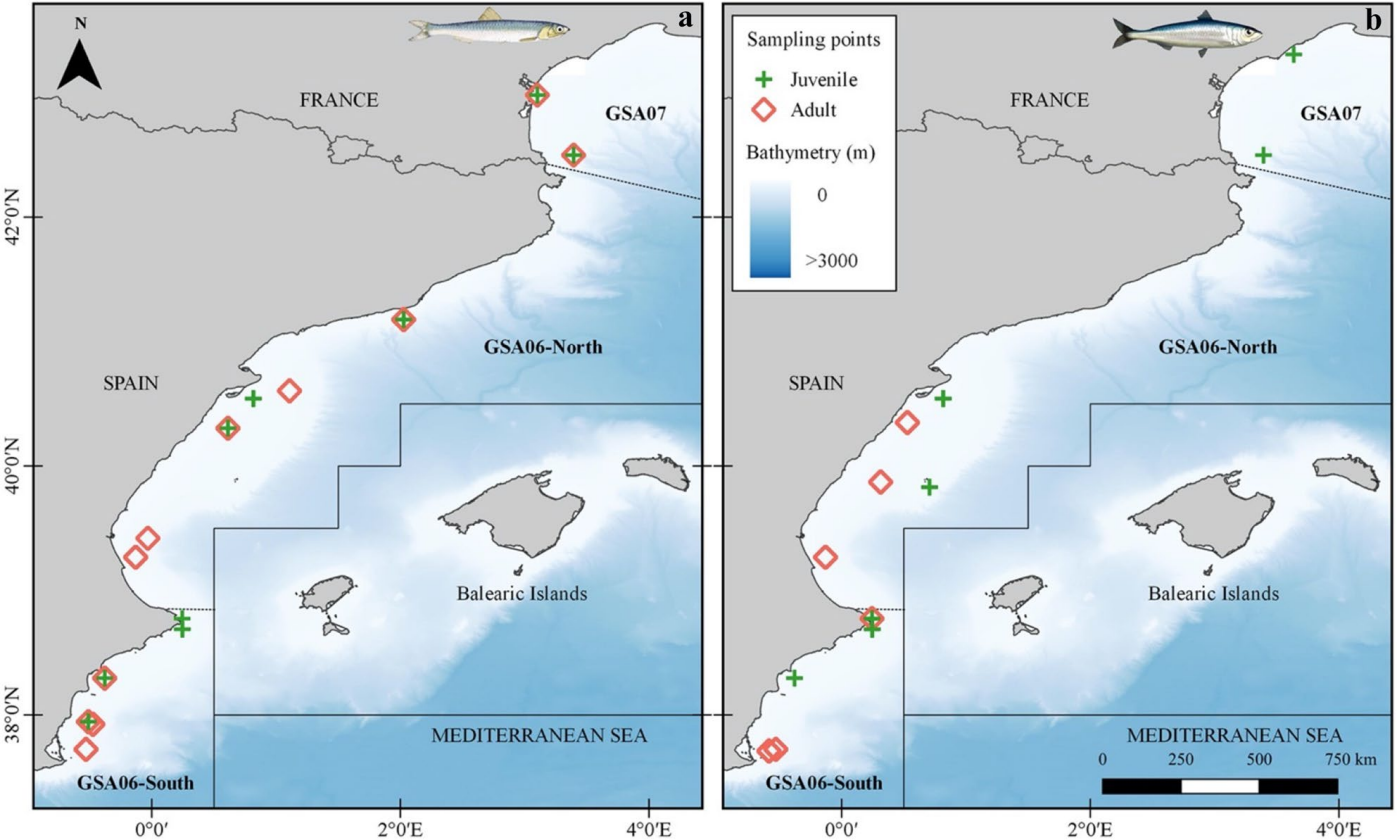
Sampling area in the Western Mediterranean Sea where (**a**) anchovy (*Engraulis encrasicolus*) and (**b**) sardine (*Sardina pilchardus*) were collected. The sampling area was divided in GSA07 (Gulf of Lion), GSA06-North (Catalan Sea and Gulf of Valencia) and GSA06-South (Gulf of Alicante). Locations where adults and juvenile individuals were collected are indicated with a red diamond for adults and a green cross for juveniles. All samples collected in the marked stations were used for gut content characterization (under the microscope and with DNA metabarcoding) and for isotope analysis (see Table 6 for details). Figure generated with QGIS v. 3.2.1-Bonn^117^ (https://qgis.org/en/site/).

Diet characterization under the microscope, DNA metabarcoding and stable isotope analyses were combined in order to assess the trophic ecology of anchovy and sardine in terms of diet composition and diet similarity between both species, ontogenetic stages (juveniles vs adults) of each species and geographical areas by a latitudinal order (Table 1). The GSA07 corresponding with the Gulf of Lion was the northernmost region of the studied area, followed by GSA06-North in the Catalan Sea and Gulf of Valencia, and GSA06-South in the Gulf of Alicante (Fig. 1).

Specifically, the following aspects were investigated (see Methods; Table 1): (1) feeding intensity and prey composition in the diet, based on individual characterization of prey items in gut contents; (2) prey diversity in gut contents and niche width, assessed with species richness, Shannon–Wiener diversity index and isotopic Bayesian standard ellipse areas (SEA_B_^37^); (3) similarity of diets, assessed by beta-diversity between anchovy and sardine in the three areas^39,40^; and (4) trophic niche and diet overlap, assessed by Pianka’s^41^ niche overlap and by isotopic SEA_B_ overlap. In order to ease the interpretation of results, juvenile vs adult comparison figures are presented as Supplementary information.

### Feeding intensity

The best and most parsimonious generalized additive model (GAM) based on significant predictors, low AIC (Akaike Information Criterion) and high D (deviance explained) values included total length of fish, latitude and depth as explanatory variables of the stomach filling degree (SFD, a proxy of feeding intensity) variability, for both species (Table S01 online). The final models explained 66.3% and 76.2% of the deviances in anchovy and sardine, respectively (Table S01a online). Adults of both anchovy and sardine (i.e. larger fish) showed relatively higher SFD values, or higher feeding intensity, than juveniles (i.e. smaller fish) (Figure S01 online; Table S01b online). Significantly higher SFD values were observed for anchovy and sardine at lower latitudes (i.e. southern areas), and also in shallower depths in the case of sardine (Figure S01 online; Table S01b online).

### Diet composition

Adults of both species ingested a broader range of prey sizes compared to juveniles, the latter demonstrating a diet composition generally dominated by copepods (Figure S02 online). Larger prey such as euphausiids and decapods were the main source of biomass in all species and areas, except for adult sardine and anchovy juveniles in GSA06-North, where copepods still represented more than half of the biomass input in the diet. Anchovy juveniles from GSA06-South also ingested large amounts of molluscs (gastropods and bivalve larvae) (Figure S02 online).

**Figure 2:**
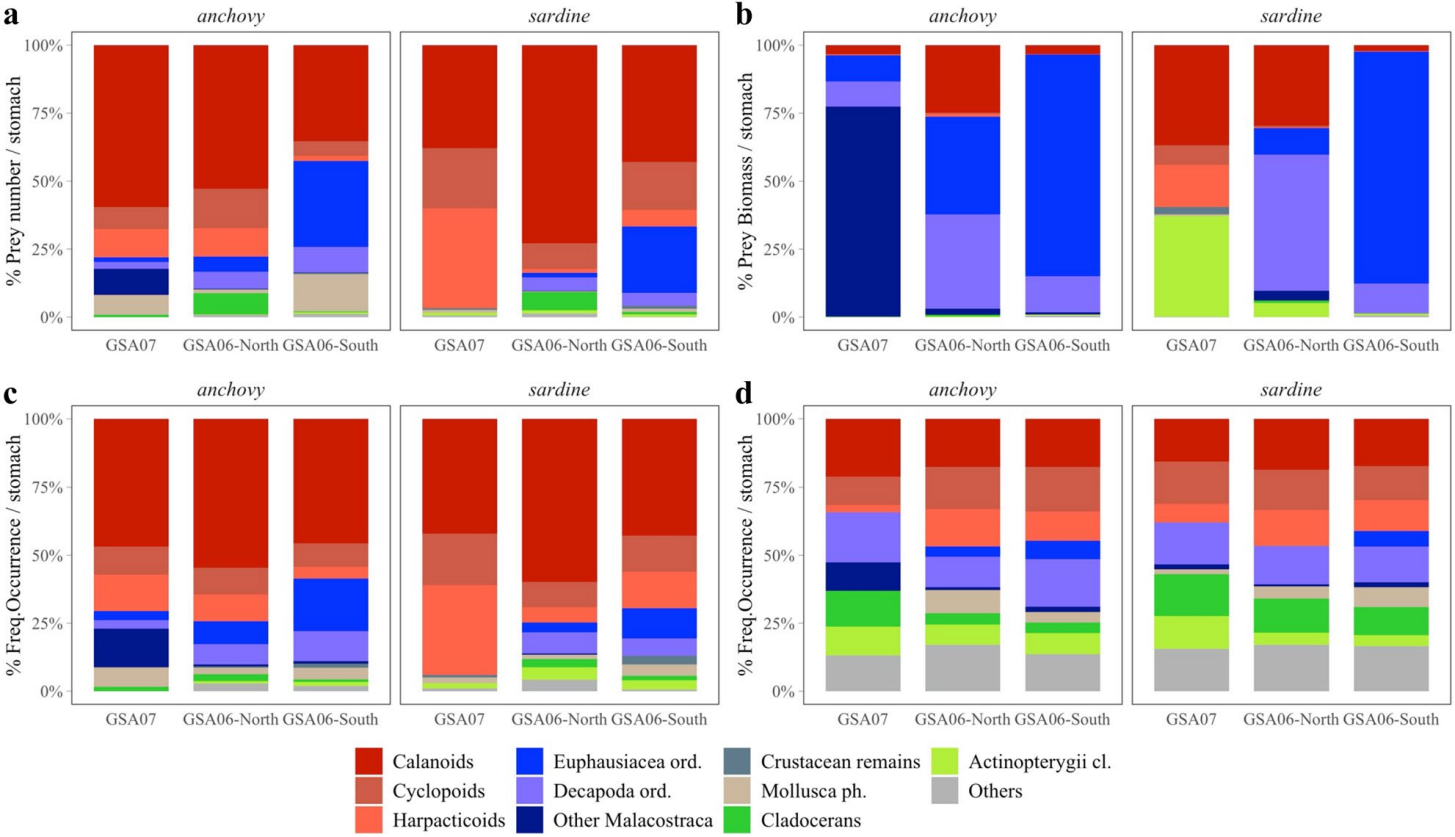
Prey group composition per stomach determined under the microscope as percentage of (**a**) mean prey abundance, (**b**) mean prey biomass, and (**c**) occurrence, and (**d**) prey group occurrence determined with DNA metabarcoding, presented as means averaged across sampling sites within areas, for anchovy (*Engraulis encrasicolus*) and sardine (*Sardina pilchardus*).

In terms of numerical frequencies, large krill was frequently ingested in GSA06-South, especially by adults of anchovy and sardine. This is in accordance with previous SFD analysis, since higher abundances of relatively larger prey in stomachs can partially explain higher SFD values in southern areas. In this sense, when GSA07 results (considering juvenile and adults together, Fig. 2a,b) were incorporated, although in general anchovy ingested relatively larger prey more frequently than sardine, some latitudinal differences were observed. While in northern areas (GSA07) anchovy preyed mainly on copepods but obtained most biomass from amphipods (i.e. ‘Other Malacostraca’, Fig. 2a,b), moving southwards, they fed more frequently on molluscs (mainly gastropod and bivalve larvae), euphausiids and decapods. Northern sardine based more than 50% of their diet on copepods (and some fish eggs contributing important amounts of biomass), and moving southwards sardine ingested more euphausiids and decapods, the highest numbers and biomass inputs coming from such large prey in GSA06-South (Fig. 2a,b).

When frequency of occurrence of prey is considered (%FO), results from the microscope analysis and DNA metabarcoding can be compared, obtaining new insights on diet composition analyses. This allows increasing the taxonomic resolution defined under the microscope to the highest (i.e. species) level, but is, at the same time, a trade-off between comparability of methods, as well as increasing the taxonomic resolution versus loosing relevant quantitative information (Fig. 2c,d, Tables S02–S07 online). Comparing both methodologies, our results showed that for both anchovy and sardine, copepods were the most abundant prey in numbers over the entire sampling area (Fig. 2a), whereas in terms of occurrence, the combination of methods showed that the relative importance of krill (i.e. euphausiids, decapods and amphipods defined as ‘other Malacostraca’) and other small prey like cladocerans or molluscs was higher than expected from the microscope analysis (Fig. 2c,d). Further, the relative importance of copepods in sardine diet from the northernmost area (GSA07) could be much lower than expected from microscope analysis (Fig. 2c), if we consider the presence of other prey groups only detected with DNA metabarcoding (e.g. decapods and other crustacean and molluscs, Fig. 2d).

Genetic methods (see detailed information in Tables S02–S04 and S06 online) allowed detecting 12 (10 demersal + 1 pelagic + 1 lantern-fish) fish species (as ‘Actynopterygii cl., Fig. 2d), which could be presented as potential evidence of intraguild predation (i.e. predation of fish egg and/or larvae) by the two species in all areas.

**Figure 3:**
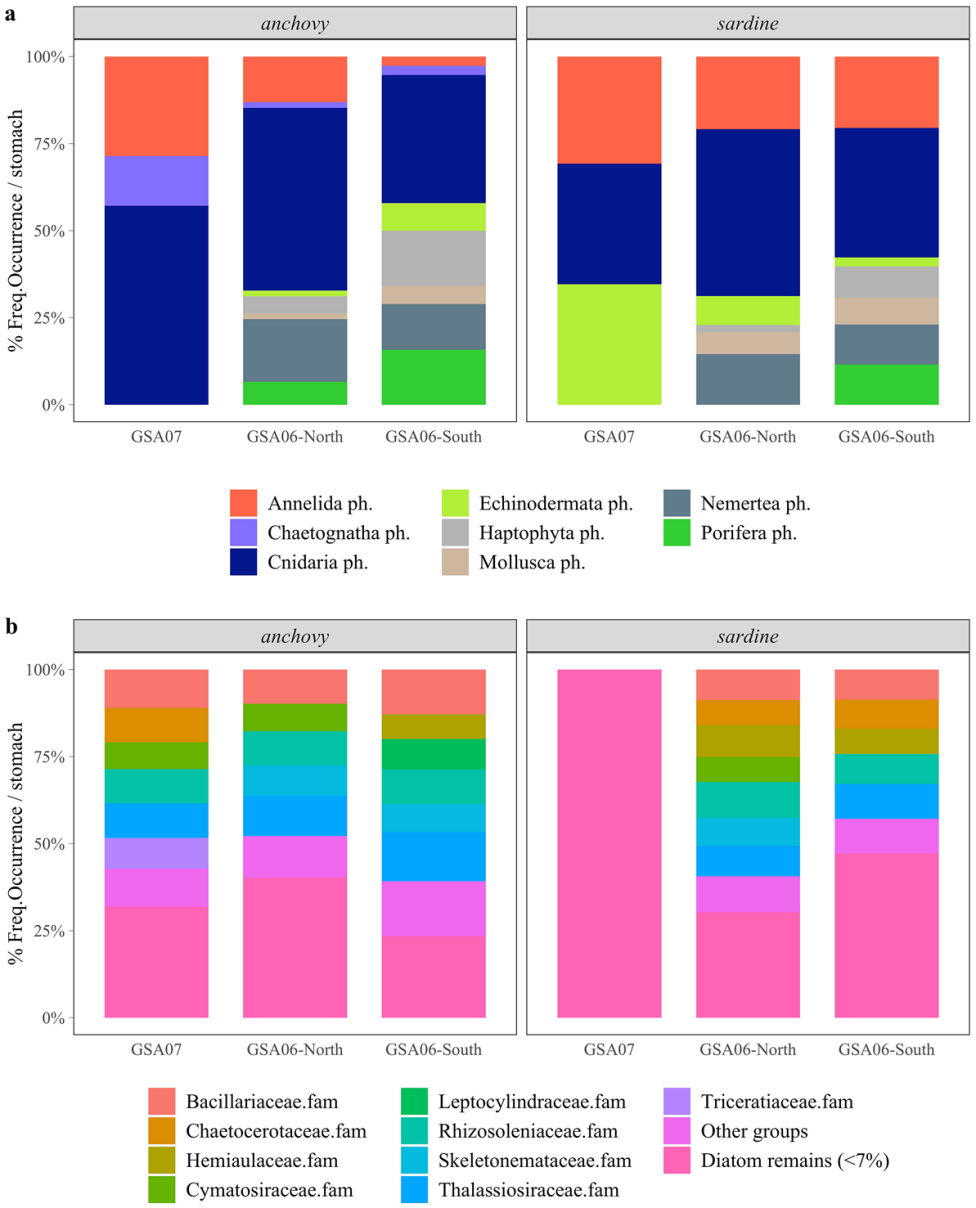
Occurrence percentage of (**a**) phyla within ‘Others’ prey group and (**b**) diatoms, determined with DNA metabarcoding and presented as means averaged across sampling sites within areas for anchovy (*Engraulis encrasicolus*) and sardine (*Sardina pilchardus*) diet.

DNA metabarcoding also indicated that the ‘Others’ group was composed of cnidarians (15 hydrozoan, 1 scyphozoan and 1 anthozoan groups), other molluscs (5 additional species), annelids (9 Polychaeta groups), echinoderms (7 groups), nemertines (1 group) and chaetognaths (1 group) (Fig. 3a). Results show that medusae were common in all areas and species, as well as annelids (with higher occurrences, at least based on percentages of detections, in GSA07) and echinoderms (especially important for sardine in GSA07) (Fig. 3a; Table S06 online).

Both anchovy and sardine ingested several taxa of diatoms, which were unidentifiable under the microscope but assessed with DNA metabarcoding (Fig. 3b). Results showed that the different species found in gut contents were ingested across the sampling area with no apparent trend. However, sardine from GSA07 seemed to have eaten different taxa from the others, as reflected by the 100% of occurrence of diatom groups that were not common (i.e. smaller occurrence % than the 70th percentile) in the other areas and species. It should also be noted that potentially harmful diatom species (i.e. within the Bacillariaceae and Chaetocerotaceae families; Table S04 online), as well as many rare species (Table S04 online), were observed in guts of anchovy and sardine (occurrence percentages of all identified taxonomic groups are presented in Table S07 online).

On the other hand, stable isotope analyses extended the diet traceability to a longer timescale, and allowed us to elucidate key differences for gut content analyses between areas for both species. Anchovy and sardine showed significant differences between areas in *δ*^13^C and *δ*^15^N (Table S08 online).

**Figure 4:**
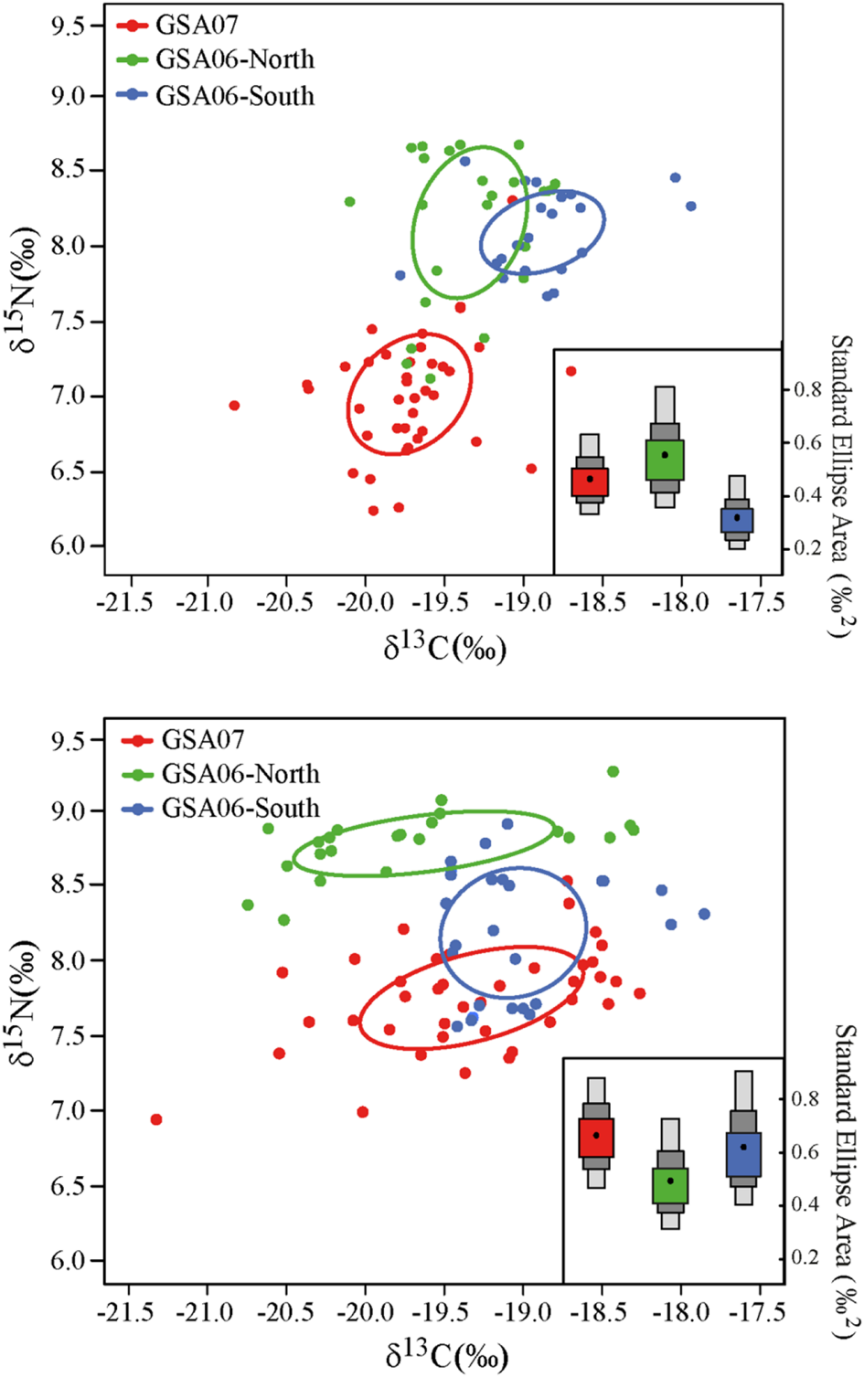
Corrected standard ellipses areas (SEA_C_; solid lines) in each area, for (**a**) anchovy and (**b**) sardine. Individual *δ*^13^C and *δ*^15^N values of GSA07 (red dots), GSA06-North (green dots) and GSA06-South (blue dots) are also graphed. Density plots showing Bayesian standard ellipses areas (SEA_B_) of (**a**) anchovy (*Engraulis encrasicolus*) and (**b**) sardine (*Sardina pilchardus*) are represented in the bottom-right of each graph as a measure of trophic niche width.

In the case of anchovy, *δ*^15^N changed from the lowest values in the north (i.e. GSA07) to the highest in the south (i.e. GSA06-South), in accordance with relatively larger prey ingested in the latter (observed in microscope analysis) (Figs. 4 and S03 online). Accordingly, the final selected GAM for *δ*^15^N of anchovy included the latitude as the only significant variable, which explained alone the 70.6% of the total variability, highlighting a negative relationship (Figure S04 online, Tables 2a and S09a online). Regarding *δ*^13^C of anchovy, the final GAM included not only the depth and latitude but also the total length of fish as significant variables. A negative linear relationship was found between latitude, depth and *δ*^13^C, while total length of fish showed a positive linear relationship. These results suggest that larger fish in shallow waters of the northern areas have higher *δ*^13^C values (Figure S05 online, Tables 2b and S09b online).

**Table 2:** Numerical summaries of the best **(a)** *δ*^15^N, **(b)** *δ*^13^C, and **(c)** Shannon diversity index (*H*’) GAMs, obtained for anchovy and sardine (see model comparisons in Table S09 online).

**A. Response variable: δ**^**15**^**N**				
**Explanatory variables**	**edf**	**Ref.edf**	**F**	***p*** **value**
**Anchovy**				
Lat	2.59	2.87	65.05	< 2e-16
**Sardine**				
TL	2.86	2.98	8.43	9.22- e-05
**Parameter coefficients**	**Mean**	**Std.Error**	**t-value**	***p*** **value**
Intercept (Area GSA06-North)	2.15	0.00	245.59	< 2e−16
Area (GSA07)	− 0.09	0.01	− 7.17	2.92e−10
Area (GSA06-South)	− 0.06	0.01	− 6.09	3.42e−10
**B. Response variable: δ**^**13**^**C**				
**Explanatory variables**	**edf**	**Ref.edf**	**F**	***p*** **value**
**Anchovy**				
Latitude	1.00	1.00	29.70	4.49e−07
Total Length (cm)	1.26	1.46	4.99	0.027
Depth (m)	1.00	1.00	4.75	0.032
**Sardine**				
Depth (m)	2.70	2.93	9.44	< 2e−16
**C. Response variable H’**				
**Explanatory variables**	**edf**	**Ref.edf**	**F**	***p*** **value**
**Anchovy**				
TL	1.47	1.61	7.16	0.00222
Lat	2.00	2.00	6.71	0.00217
TL: Lat	7.87	8.89	4.69	8.85e−05
**Sardine**				
TL	2.92	2.99	3.12	3.07e−02
Lat	1.00	1.00	6.55	12.6e−03
TL:Lat	6.80	6.98	5.29	4.17e−05

Statistics acronyms are: edf = degrees of freedom, Ref.edf = relative degrees of freedom, F = F statistic, Std.Error = Standard error.

For sardine, a similar latitudinal trend was observed for *δ*^15^N (i.e. higher values in GSA06 than in GSA07; Figs. 4 and S03 online, Tables 2a and S09a online). In the selected GAM for *δ*^15^N, the final significant variables were the total length of fish and the area factor, which jointly explained 67.4% of the total variability (Figure S04 online, Tables 2a and S09a online). In particular, results highlight the lowest *δ*^15^N values in GSA07 with respect to GSA06-North (i.e. reference level, mean = − 0.09, sd = 0.01) and GSA06-South (mean = − 0.06, sd = 0.01) (Table 2a). Higher values of *δ*^15^N were found in larger (i.e. adult) fish (Figure S06 online, Table S08). The selected GAM model for *δ*^13^C showed bathymetry as the only significant variable, explaining 28% of the total *δ*^13^C variability and highlighting a decreasing trend from 80 m depth (Figure S05 online, Tables 2b and S09b online).

#### Species richness and diversity

**Figure 5:**
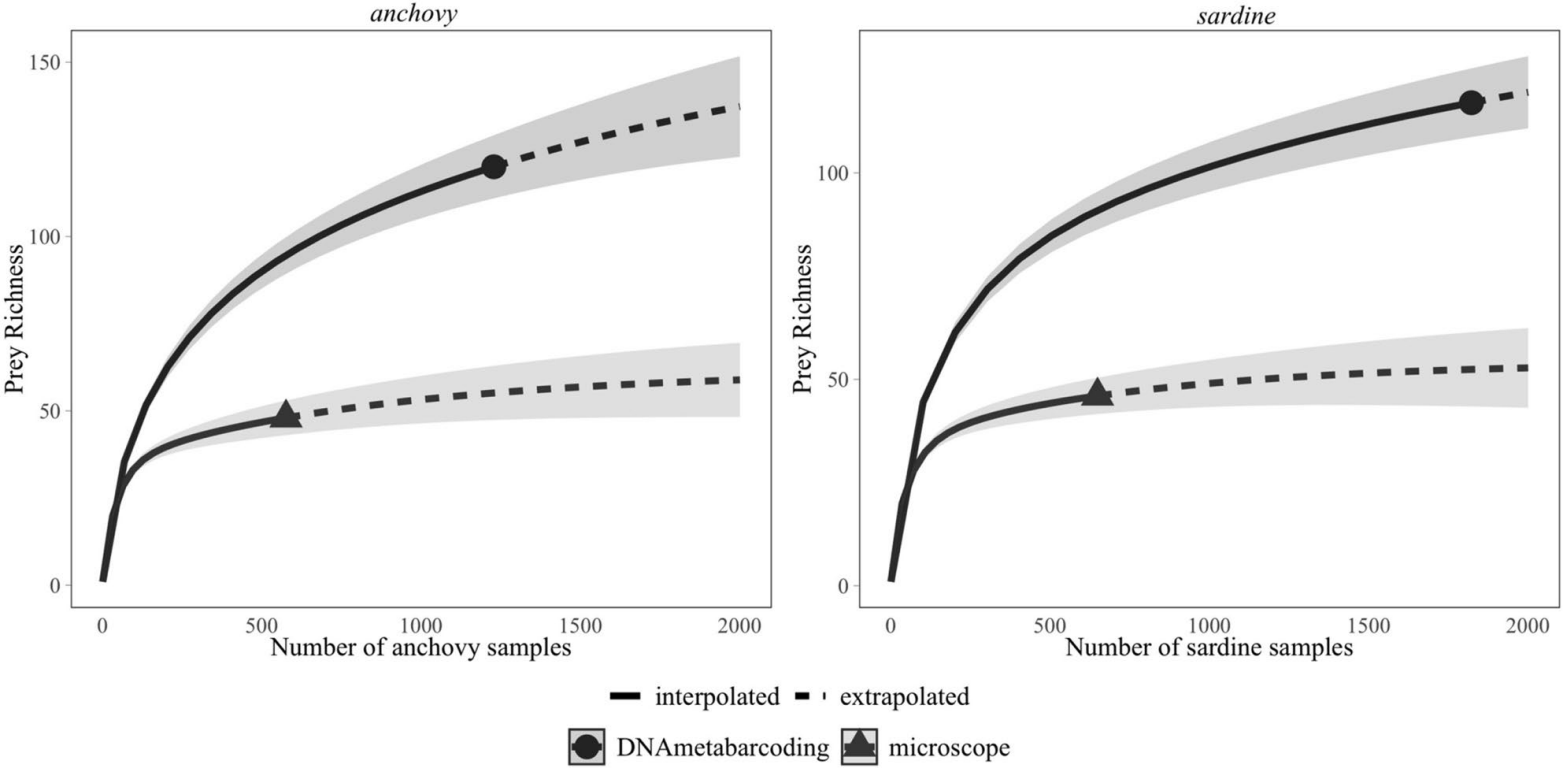
Sample-size-based rarefaction (solid line segments) and extrapolation (dotted line segments) sampling curves with 95% confidence intervals (shaded areas) for prey species richness obtained analyzing gut contents of anchovy and sardine under the microscope and with DNA metabarcoding. The solid dots/triangles represent the reference samples. Graphical representation was made according to Hsieh et al.^118^.

As expected from the previous section, prey species richness as well as the Shannon–Wiener diversity index in diets were the highest in the southernmost area (GSA06-South, Table 3). A total of 144 prey groups were identified with DNA metabarcoding, 2.6 times more than the 55 groups identified under the microscope. DNA metabarcoding detected higher numbers of prey species (Fig. 5) in all taxonomic groups, e.g. copepods (40 vs 28 taxa), molluscs (10 vs 2), decapods (24 vs 4 taxa), fish (14 vs 3) or groups merged as ‘Others’ (42 vs 3 taxa).

**Table 3:** Species richness and diversity in gut contents of anchovy (*E.enc*: *Engraulis encrasicolus*) and sardine (*S.pil*: *Sardina pilchardus*), averaged by prey groups (number of prey species), areas and methods. ‘Total’ denotes results for all the areas together. Shannon diversity index, calculated for gut contents analysed under the microscope, is presented as the mean ± standard error. Pairwise beta-diversity was calculated for anchovy and sardine according to Koleff et al.^39^.

	Microscope analysis								DNA metabarcoding							
	GSA07		GSA06-North		GSA06-South		Total		GSA07		GSA06-North		GSA06-South		Total	
	*E.enc*	*S.pil*	*E.enc*	*S.pil*	*E.enc*	*S.pil*	*E.enc*	*S.pil*	*E.enc*	*S.pil*	*E.enc*	*S.pil*	*E.enc*	*S.pil*	*E.enc*	*S.pil*
Calanoids	8	7	16	11	14	13	16	13	10	14	19	22	25	26	28	29
Cyclopoids	2	2	3	4	4	4	5	5	1	2	4	2	4	5	5	5
Harpacticoids	3	4	5	5	3	5	5	6	1	2	4	3	4	4	5	4
Euphausiacea ord	3	0	5	3	5	6	5	6	0	0	3	0	2	2	3	2
Decapoda ord	1	0	4	4	4	4	4	4	7	9	15	11	12	16	19	19
Other Malacostraca	3	0	2	1	2	0	4	1	3	1	1	1	2	3	5	4
Crustacean remains	0	1	1	0	2	2	2	3	0	0	0	0	0	0	0	0
Mollusca ph	2	1	2	1	2	2	2	2	0	1	3	5	9	6	10	7
Cladocerans	1	0	1	1	1	1	1	1	1	4	3	4	2	3	3	4
Actinopterygii cl	0	1	1	2	2	2	2	2	4	4	4	5	7	5	12	9
Others	0	1	1	1	2	2	2	3	6	11	22	30	26	23	31	35
*Diatoms	*0*	*0*	*0*	*0*	*0*	*0*	*0*	*0*	*26*	*32*	*36*	*39*	*32*	*36*	*38*	*41*
Species Richness	23	17	41	33	41	41	48	46	33	48	78	83	93	93	121	118
Shannon Diversity	1.27 ± 0.09	1.29 ± 0.08	1.57 ± 0.10	1.50 ± 0.11	1.72 ± 0.11	1.77 ± 0.07	1.52 ± 0.17	1.56 ± 0.17	–	–	–	–	–	–	–	–
Beta-Diversity	0.35		0.24		0.17		0.14		0.44		0.28		0.27		0.20	

*Diatoms were excluded in diversity calculations.

In addition, DNA metabarcoding detected 41 species of diatoms, not observed under the microscope. This group was excluded from prey richness and diversity comparisons in order to make results comparable.

While differences within copepods were largely due to taxonomic identification (Table S02), most of fish, decapods and groups within ‘Others’ were only detected with DNA metabarcoding (Fig. 3a; Table S03 online). This resulted in higher prey richness indices than those obtained with microscope analysis, and small inter-specific differences regarding certain prey groups. For example, in GSA07, prey richness in anchovy and sardine were different depending on the method used to detect prey, possibly due to differences in the number of identified copepod species (Table 3). Nevertheless, relative spatial differences remained similar between DNA metabarcoding and microscope analysis.

Accordingly, inter-specific differences in prey richness and diversity were generally low within each area, whereas a latitudinal decreasing trend in both prey richness and prey diversity was observed, which is partially related to the widening of the prey spectrum in the diet of both anchovy and sardine in the south (Table 3). In fact, the final selected GAMs showed a significant negative relationship between Shannon index and latitude for both anchovy and sardine, as well as a positive relationship with the length of fish (Figure S07 online, Tables 2c and S09c online). The interaction between the length of the fish and the latitude was significant for both species and highlighted that smaller fish sampled in the northernmost areas (GSA07) showed lower prey diversities than the larger fish sampled in the south (GSA06-South), with higher prey diversity in their diet composition (Table S12 online). Overall, the best GAMs explained 29.6% and 57.5% of the deviance for anchovy and sardine prey diversity, respectively (Table S09c online).

Regarding the isotopic niche width (Bayesian standard ellipse area, SEA_B_^36^), contrary to what was observed with gut content analyses both anchovy and sardine showed a wider niche in GSA07. Specifically, in the case of anchovy the area that presented the widest SEA_B_ was the GSA06-North followed by GSA07. The GSA06-south was the area where anchovy had the narrowest isotopic niche width. In the case of sardine, in the three areas the isotopic niche width was similar with higher values of SEA_B_ for GSA07 and GSA06-South than GSA06-North, mainly due to a wider variability in *δ*^13^C values (Fig. 4; Figure S06 online).

#### Similarity of diets, trophic niche and diet overlap

In order to compare the similarity of diets between anchovy and sardine for each area, determined by microscope and DNA metabarcoding analyses, a beta-diversity approach was applied for anchovy and sardine with prey presence-absence data^39^. Assuming the same diet activity for both species, results obtained with both microscope analysis and DNA metabarcoding showed that anchovy and sardine shared the same niche in all areas equally (i.e. beta-diversity values were closer to 0 than to 1, see Methods). The highest differences between methods (i.e. microscope and DNA metabarcoding) were obtained in GSA06-South, probably due to the remarkable increase in numbers of copepods, decapods and mollusc species identified by DNA metabarcoding analyses. Accordingly, the inter-specific diet similarity was the highest in GSA06-South, where higher beta-diversity values were obtained compared to GSA07 (Table 3).

A high diet overlap (*O*) was found between anchovy and sardine, regardless of the methodology used for the analysis. For the whole sampling area, the *O* index was 0.98 and 0.96, considering diets based on microscope analysis and DNA metabarcoding, respectively. The *O* index was > 0.85 when considering each area separately (Table 4). When the overlap indices are compared between areas, the diet overlap was the highest between GSA06-North and GSA06-South for both species (*O* > 0.95, Table 4). The lowest diet overlap was observed for sardine between GSA07 and GSA06-North based on microscope analysis, but DNA metabarcoding also revealed a high degree of overlap in this case. In contrast, anchovy seemed to show lower niche overlap between these two areas regardless of the methodology used to analyse gut contents (Table 4).

**Table 4:**
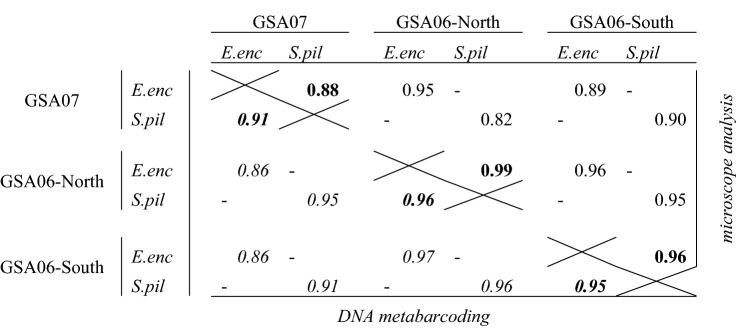
Pairwise contingency table presenting Pianka index of niche overlap between species (*E.enc*: anchovy; *S.pil*: sardine) and areas. Results in the top-right part of the table correspond to diet information obtained from visual microscope analysis, whereas the results in the bottom-left part of the table (numbers in italics) are based on DNA metabarcoding analyses.

Numbers in bold represent species comparison of overlap indices within a certain area, and light numbers, the overlap indices between areas (i.e. considering the species separately).

When calculating trophic overlap with stable isotope data, a longer temporal period than the one covered with gut content analysis is integrated, obtaining information not only based on the prey consumed but also on the assimilated food. Thus, such overlap analysis of isotopic data (SEA_B_) between areas (i.e. GSA07, GSA06-North and GSA06-South) presented a general smaller overlap for each species (Fig. 4, Figure S06 online, Table S10 online). Some overlap was detected between GSA06-North and GSA06-South for anchovy (17.96% and 11.07% overlap) and between GSA07 and GSA6-South for sardine (30.16% and 28.17% of overlap). Regarding the overlap between species in each subarea, smaller overlap was detected except for GSA6-South, where a medium level overlap was apparent (27.53% and 54.80% overlap). SEA_B_ overlap values and credible intervals are presented in Table S10 online).

## Discussion

The gut contents characterized in this study is in accordance with the dietary plasticity generally described for anchovy and sardine^13,17,42–46^, suggesting that the diet composition of both pelagic species is generally related to the opportunistic ingestion of available prey in a certain area and/or at a certain point in time^47,48^. Genetic tools indicated small inter-specific differences regarding ingested species, which also supports the opportunistic feeding and the ability of both species to switch between particulate and/or filter feeding strategies according to prey availability. However, inter-specific and intra-specific differences in ingested prey frequencies and the ingested prey biomass reflected a latitudinal signal that could illustrate a more effective predation on large prey like krill by anchovy than by sardine, e.g. especially in the *north* (hereafter referring to GSA07), as well as a generalized higher large prey ingestion by both species as they move *southwards* (hereafter referring to GSA06-South as the southernmost area) (Table 5).

**Table 5:** Summary table showing comparative results regarding the latitudinal gradient hypothesis of the study, depending on species.

	Method	*GCC*		*SIA*		*DNA-M*	
Variable (metrics)	Species	*E.enc*	*S.pil*	*E.enc*	*S.pil*	*E.enc*	*S.pil*
Diet characterization	*SFD*	S > N	S > N	–	–	–	–
**%ABD**							
	copepods	N > S	N > S	–	–	–	–
	krill	S > N	S > N	–	–	–	–
	others	NLD	NLD	–	–	–	–
**%BIO**							
	copepods	NLD	N > S	–	–	–	–
	krill	NLD	S > N	–	–	–	–
	fish eggs/larvae	NLD	N > S	–	–	–	–
	other prey	NLD	N > S	–	–	–	–
**%FO**							
	copepods	NLD	N > S	–	–	NLD	NLD
	krill	NLD	S > N	–	–	NLD	NLD
	fish eggs/larvae	NLD	NLD	–	–	NLD	N > S
	cnidarians	–	–	–	–	N > S	NLD
	other prey	NLD	NLD	–	–	NLD	NLD
**S (values)**							
Prey diversity/niche width	[total]	S > N	S > N	–	–	S > N	S > N
	copepods	S > N	S > N	–	–	S > N	S > N
	krill	S > N	S > N	–	–	S > N	S > N
	fish eggs/larvae	S > N	S > N	–	–	S > N	NLD
	other prey	S > N	S > N	–	–	S > N	S > N
	diatoms	–	–	–	–	NLD	NLD
	*S* (rarefaction curves)*	DNA > GCC	–	DNA > GCC	–	–	–
	*H* and SEA_B_ area	NLD	NLD	N > S	NLD	–	–
Diet similarity	${\beta }_{w}$	N < S	–	N < S	–	–	–
Niche overlap	*O* and SEA_B_ overlap	NLD	S > N	NLD	–	–	–

(*E.enc*: *Engraulis encrasicolus*; *S.pil*: *Sardina pilchardus*), methods (*GCC*: gut content characterization; *DNA-M*: DNA metabarcoding; *SIA*: stable isotope analysis) and metrics (*SFD*: stomach filling degree; *%ABD*: percentage of prey number; *%BIO*: percentage of prey biomass; *%FO*: percentage of prey occurrence; *S*: prey richness; *H*: Shannon diversity index; ${\beta }_{w}$: beta-diversity; *O*: Pianka overlap index; SEA_C_ & SEA_B_: corrected and bayesian standard ellipses areas, respectively). “Copepods” refers to Calanoids, Cyclopoids and Harpacticoids; “Krill” refers to “Euphausiacea ord.”, “Decapoda ord.” and “Other Malacostraca”; “Fish egg/larvae” refers to “Actinopterygii cl.”; remaining groups are presented as “Other prey”. “NLD” means no latitudinal difference (i.e. < 30% difference). Paired comparisons are made between the northernmost (GSA07, referred as N) vs. the southernmost (GSA06-South, referred as S) areas.

“*” indicates comparison between methods.

“ > ” and “ < ” indicate in which area the magnitude of the comparison is higher.

Combining the stomach filling degree (SFD) approach with numerical prey frequency and prey biomass analyses, it seemed clear that predation on large prey is of great interest in terms of energy intake. Not only northern anchovy but also both species in the south get most of the biomass input from decapods, euphausiids and other malacostracans. It might be expected that such opportunistic active predation widens the prey size spectrum as fish grow, and that adult fish ingest higher numbers of larger prey^49^ due to their energetic requirements (e.g. for reproduction^50^) and a larger gape (i.e. mouth) width^42^. This high voraciousness in adults might also explain the higher SFD determined in adult anchovy and sardine. This does not necessarily mean that juveniles cannot get some success feeding on relatively larger prey. This is the case in GSA06-North, where sardine juveniles ingest important numbers of krill, in contrast with anchovy juveniles that get > 50% biomass from copepods; or in the south, where molluscs are often found in guts contents of anchovy juveniles. Such opportunistic predation by small (i.e. juvenile) anchovy and sardine has also been previously reported e.g. in the Bay of Biscay^42,43,51^.

As large prey are incorporated in the diet, prey richness and diversity also tend to increase, as reflected especially in the southern area for both species (also observed in the modelling results) (Table 5). This trend is in accordance with previous observations in the Bay of Biscay^43^, but also contrasts with sardine studies a decade ago in North Aegean Sea that showed higher prey diversities related to higher phytoplankton ingestion by large fish^16^. The fish size range in the northernmost area was smaller than in the other areas (e.g. sardines were all juveniles, Table 6; sampled fish were smaller in GSA07, Figure S08 online). This may be caused by a lower growth and poorer feeding condition of northern Mediterranean fish observed in recent years by other authors^20,21,52^. However, further research is required in order to determine a hypothetical size-dependent latitudinal feeding behaviour, while considering that sardine can effectively filter even the smallest prey sizes during their whole life-cycle^13,16,17,46,53^. In any case, in this study the major diversity compound is based on copepod ingestion, with several ingested species (33 and 43 groups based on microscope analysis and DNA metabarcoding, respectively), most likely determined by the available zooplankton in the area^14,49,54^. This results in low inter-specific or spatial differences and a relatively high degree of diet overlap (especially in the south). Then, when the number of ingested prey groups is low (e.g. by microscope analysis in northern anchovy and sardine), the diet dissimilarity (i.e. inter-specific difference) is relatively high. In contrast, incorporating DNA metabarcoding increases the number of detected species and therefore the beta-diversity^39^, resulting on lower spatial inter-specific differences in such diet dissimilarities. Nevertheless, latitudinal differences remain clear regardless of the method applied, i.e. anchovy and sardine sharing the niche and showing higher diet similarity in the south (Table 5).

**Table 6:** Number of stomach samples (*N*) and total length (*TL*) range (i.e. minimum–maximum length in cm) of anchovy.

		TL (cm)	Gut content characterization (N)	DNA metabarcoding (N)	TL (cm)	Stable Isotope Analysis (N)
GSA07	*E.enc*	8.50–11.00	40 (*juv*)	10 (*juv*)	8.50–12.40	26 (*juv*) + 13 (*ad*)
	*S.pil*	9.60–12.80	40 (*juv*)	10 (*juv*)	9.60–12.90	38 (*juv*)
GSA06-North	*E.enc*	8.90–14.80	15 (*juv*) + 20 (*ad*)	15 (*juv*) + 20 (*ad*)	8.90–14.50	14 (*juv*) + 14 (*ad*)
	*S.pil*	7.80–16.90	15 (*juv*) + 15 (*ad*)	15 (*juv*) + 15 (*ad*)	7.80–16.90	12 (*juv*) + 12 (*ad*)
GSA06-South	*E.enc*	9.20–16.20	6 (*juv*) + 22 (*ad*)	6 (*juv*) + 22 (*ad*)	9.20–16.20	6 (*juv*) + 16 (*ad*)
	*S.pil*	8.50–17.80	15 (*juv*) + 19 (*ad*)	15 (*juv*) + 15 (*ad*)	8.50–17.70	12 (*juv*) + 15 (*ad*)
**All samples**	*E.enc*	8.50–16.20	103	73	8.50–16.20	89
	*S.pil*	7.80–17.80	104	70	7.80–17.70	89

(*E.enc*: *Engraulis encrasicolus*) and sardine (*S.pil*: *Sardina pilchardus*). Results are presented by area, ontogenetic stage (*juv*: juveniles; *ad*: adults) and method applied for the analyses: gut content characterization under the microscope and DNA metabarcoding (i.e. the same fish were used for these two methods), and Stable Isotopes

On the other hand, the isotopic niche overlap gives contrasting results compared to gut content data. The higher *δ*^15^N values of sardine in the northern and central part in comparison with anchovy values, explain the segregation of both species. Such higher values of *δ*^15^N are usually associated with the ingestion of prey with higher trophic position^55^. In the north, inter-specific differences might be explained by the higher biomass obtained from fish eggs and/or larvae (i.e. Actinopterygii cl.) by sardine as well as by the high biomass input coming from decapods for anchovy. Instead, in the south the niche overlap between anchovy and sardine is higher, similar to the results from gut content analysis. Such an overlap between sardine and anchovy has also been previously reported^14,20,47,56^. When comparing latitudinally, both species present lower *δ*^15^N in the northernmost area, which is in agreement with the observed prey composition^20^, since a higher presence of larger prey in the south than in the north might also explain differences in the *δ*^15^N signal. Also, sardine and anchovy individuals were smaller in GSA07 than in the other areas, which could partially explain the lower *δ*^15^N values in the northernmost area. However, the isotopic niche width is similar across areas for both species, with slightly lower values in the south for anchovy and in the centre for sardine. The longer time-integrated period (e.g. weeks, months) reflected in stable isotopes in contrasting with the snapshot represented by gut content analyses could explain such differences in these results. Differences in the information provided from metrics on gut content and stable isotopes have also been highlighted in previous studies^57^.

In addition, information obtained from certain prey species highlights differences both in terms of feeding efficiency and potential (i.e. energetic) interest in the diet. For instance, DNA metabarcoding allows determining several (previously underestimated) decapod species, such as *Solenocera membranacea*, indicator of muddy or sandy-muddy bottoms within the continental shelf and/or slope^58^, which occurs in higher percentage of sardine gut contents in the north, as well as in anchovy gut contents in the south (Table S07 online). Similarly, DNA remains of other species like the annelid *Magelona* sp. and starfish *Paracentrotus lividus* were also identified in both fish species all over the sampling area, suggesting that the feeding activity of anchovy and sardine might also affect to (most likely) pelagic early-life stages of demersal species. Moreover, when samples were collected during the daytime (see Methods), both anchovy and sardine could also be feeding in deeper areas^17,59^, which highlights the importance of these species not only for pelagic but also for the demersal-pelagic energy transfer^60^. On the other hand, DNA metabarcoding reveals an important presence of fish eggs and/or larvae in (mainly juvenile fish) gut contents, especially of demersal species, such as *Trisopterus capelanus* and *Spicara maena*, but also of pelagic species like *Sprattus sprattus* (mainly sardine predation). However, just with occurrence data it is difficult to determine whether such predation might cause potential (negative) effects in survival indices of early life stages of potential prey and competitors^61–63^.

In any case, our study showed that both anchovy and sardine took advantage of whatever prey species was available for them; and fish predation, as well as the predation on relatively large prey groups, may occur especially during late hours of the day and/or night, when such prey are more vulnerable to active predation^18^. The same may apply for the predation on jellyfish, which have not been detected in the diet of anchovy and sardine before this study. Such jelly organisms might be considered as especially vulnerable during the night, and were mainly identified by DNA metabarcoding probably because they were mostly digested by the time of fish sample collection (i.e. daytime). Determining jelly organisms ingestion by fish is of special interest, since such expanding organisms could be considered indicators of global warming and changes in the pelagic ecosystem^64,65^. However, it is not clear whether hydrozoans have not been ingested in the past, or the lack of such information is related to methodological issues. Recently, other jelly organisms like salps have been detected as part of the diet in sardinella (*Sardinella aurita*) within the same area^66^, which is in accordance with the observed ingestion of siphonophores such as *Nanomia bijuga* and *Muggiaea atlantica*, or hydrozoans like *Clytia hemisphaerica*, and medusae *Lizzia blondina* and *Aglaura hemistoma*, all of them often detected as DNA remains of gut contents in anchovy and especially in sardine in this study (Table S07 online). Further research is needed to determine potential consequences of the increase in numbers of cnidarians that might be expected in the diet of anchovy and sardine in a near future due to environmental changes.

Genetics also determined that both anchovy and sardine ingest diatoms. It is generally known that phytoplankton is more important in the diet of sardine due to their more effective filtering apparatus (i.e. gill-raker size^46,67^), and the occurrence and diversity of phytoplankton in gut contents is higher in sardine. According to our study, the ingested diatom species by anchovy and sardine are most likely determined by the phytoplankton diversity in the feeding area during the sampling period; such diatoms might have been directly ingested through filter feeding, or even as part of the zooplankton ingested by fish prey, such as copepods and other phytophagous crustaceans or molluscs. That said, for instance, *Chaetoceros* spp. and *Thalassiosira* spp., known as common spring bloom species in the Mediterranean^68–70^, are found in guts from both anchovy and sardine. Other diatoms such as *Minidiscus trioculatus*, reported as ‘extremely rare’ in previous work^71^ are commonly found in samples of both anchovy and sardine in the present study (Tables S05 and S07 online), indicating a potential expansion of such species distribution in the Mediterranean during recent years. The random phytoplankton ingestion is also confirmed with the observed harmful diatom species^70^, such as *Pseudo-nitzschia* spp., *Chaetoceros socialis* and *Cerataulina pelagica* (Tables S05 and S08 online), which might not cause further problems in anchovy or sardine population, unless blooms of this kind of algae occur.

It should be noted that DNA metabarcoding have some limitations when is used in diet-related studies, such as DNA degradation from the time of feeding until the sample collection, DNA extraction efficiency, selection of molecular markers, PCR inhibition or uncompleted reference databases^72–74^. In addition, DNA metabarcoding without microscope analysis does not determine the trophic position of each detected prey species in the food web. In the same way, further development is needed in this method in order to quantify the detected prey, which would increase the sampling coverage and the diet characterization in the laboratory more cost-effective. However, at this stage our DNA metabarcoding approach has increased the detection success of prey species and, complementary to the results obtained from the other two methods, provides relevant information of anchovy and sardine diet composition.

In summary, this study reports a latitudinal gradient in the diet composition of both anchovy and sardine in the Western Mediterranean Sea, which may reflect a widening of the prey spectrum for both anchovy and sardine as they move southwards (Table 5). All applied methods show that the northern area is the most different one, where both species showed a higher inter-specific difference in the diet probably related to poorer feeding conditions (i.e. less numbers or relatively large prey^20^) that lead to higher phytoplankton and small copepod ingestion especially by sardine, and a more effective opportunistic (active) predation on large prey by anchovy^3,13,16,17,20,21,53,54^. The largest ingestion of biomass in the diet of both species came from large prey, such as fish eggs and cnidarians in the north for sardine and anchovy, respectively, or krill, ingested by all fish all over the sampling area but with higher apparent success in the south. In fact, according to this study, in the south, inter-specific feeding differences were smaller, most likely because anchovy and sardine took advantage of more abundant and diverse prey groups, including a wider range of large prey. The generalist feeding behaviour of anchovy and sardine is known to result in intra-specific individual variation in the diet composition^42,43^, as observed also in the present study.

Overall, every method used in this study provided specific diet information of anchovy and sardine, and methodological differences were mainly reflected in the obtained absolute numbers (e.g. higher diversity and richness values obtained with DNA metabarcoding, Fig. 5)^22^. Only when all of the methods were combined, a global insight of the feeding ecology could be obtained. This is the very first time these three different methods are combined to investigate feeding ecology of pelagic fish in a broad spatial scale. This is indeed the greatest contribution of this work as, for instance, when considering different time scales, the whole prey size range and other potential interactions could be often underestimated when using methods separately. It should be noted also that despite the use of different methods, the latitudinal trends were clearly detected by all methods, illustrating changes of the trophic ecology of anchovy and sardine in the Western Mediterranean Sea. This latitudinal gradient matches with the ecological gradient previously described and the better conditions of both species in the southern area in recent years^9^. The evidenced inter-specific latitudinal difference is fundamental for further multidisciplinary approaches that could integrate different aspects such as fish distribution^2,75^, plankton availability^76^, fish growth^20,52^ or the potential ecological effects of microplastic ingestion by fish^77,78^.

## Methods

### Sample collection

Fish samples of anchovy and sardine were collected in the Western Mediterranean area (Fig. 1) in Geographical Sub-Areas 6 and 7 (GSAs) defined by the General Fisheries Commission for the Mediterranean (GFCM^1^), during the MEDITS 2018 survey (Mediterranean International bottom Trawl Survey). MEDITS was considered an ideal survey for obtaining standardized information (i.e. abundance index by swept area method), as researchers use the same sampling protocol throughout the Mediterranean Sea^79^. Therefore, the sampling procedures were standardized according to a common protocol over GSAs and years. The standard fishing device was a bottom trawl GO73 with 20 mm cod-end mesh size net^80^. The average vertical opening of the gear was 2 m and its wing-span was 18 m. All the tows were performed during daylight hours. The samples of the study were collected during the months of May and June, 2018.

### Gut content characterization under the microscope

Gut contents of 103 anchovy and 104 sardine samples (Table 6) were analysed individually, with no subsampling, under a NIKON SMZ1270 stereomicroscope with 20–80 × amplification. The laboratory practice followed the standard procedures to avoid contamination during sample processing. Accordingly, microscope analysis was conducted in a ‘clean room’ and with an air extractor placed 20–30 cm above the petri plate with the gut samples. To avoid contamination between samples, glassware, bench, microscope slide and dissection equipment (i.e., stainless-steel scissors, scalpel and lancet) were rinsed with 96% ethanol prior to each gut content analysis^81^.

In order to exclude bias caused by different rates of digestion and cod-end feeding^82^ only material contained in the stomachs was considered, whereas the contents of the intestine and oesophagus were discarded. During processing, stomach contents were carefully taken apart and all identifiable prey counted and specified to the lowest possible taxonomic group, not including broken parts of appendixes when quantifying, and categorized into 59 groups. Parasitic organisms found in stomachs (e.g. Trematoda and Nematoda larvae) did not show any relationship either with total prey abundance in gut contents or with stomach fullness, so they were excluded from the diet analysis. After the microscope analysis, stomach contents were preserved on 96% ethanol for later DNA metabarcoding analysis.

The feeding intensity was assessed calculating the stomach filling degree (SFD). This parameter is a useful qualitative metric that allows determining if feeding intensity (or efficiency) is relatively higher in a certain area and/or time^43,83^. In order to exclude the effect of fish size, the response variable SFD was defined as the sum of the weights of all the prey in a stomach (mg) divided by the total length of the fish (mm).

The diet composition was first explored using numerical and weight percentages of prey groups relative to total prey consumption. To determine the weight of each prey group, length − weight conversion equations were used based on literature. Average total length of prey species (mm) was obtained from Bachiller & Irigoien^42^, where direct length measurements were made for the first 50 prey items in each stomach. Since most of prey species observed in the present study were also observed in the Bay of Biscay, conversion of prey counts into biomass was made to the detailed species level. For missing prey species or groups (i.e. observed only in the Mediterranean), biomass of the same genera or the corresponding upper taxonomic level was assigned (Table S03 online). To exclude the effect of the sample size (number of fish per station) on the identified prey abundance, the biomass of each prey group was weighted by the number of fish per predator species and area.

Diet composition was presented as percentages of the total prey consumption for the three areas when considering all sampled fish, and for GSA06-North and GSA06-South also by ontogenetic stages (i.e. adults vs. juveniles); fish < 11 cm and < 13 cm were considered as juveniles for anchovy^84,85^ and sardine^86^, respectively.

To ease later interpretation of the figures, prey groups were categorized into 11 groups (see Table S04 online): Calanoids, Cyclopoids, Harpacticoids, Euphausiacea ord., Decapoda ord., Other Malacostraca, Crustacean remains (i.e. undefined taxa and/or broken parts of organisms within Crustacea subph.), Mollusca ph., cladocerans, Actinopterygii cl., and Others (including the rest of the groups with a frequency in number < 5% of the total prey consumption observed under the microscope). This ‘Others’ group was then broken down in detail, based on results from the DNA metabarcoding and presenting 38 taxonomic groups, merged into 8 phyla (Table S04). Finally, the diatom (Bacillariophyta ph.) content in gut contents of anchovy and sardine was also assessed by the DNA metabarcoding (see procedures below), detecting 41 different algae taxa, merged into 11 groups (i.e. based on family taxonomic level, and presenting algae with < 7% of the total algae occurrence as ‘Diatom remains’ for graphical representation; Table S05 online).

Prey species richness was defined as the number of different prey groups found in gut contents. The diet diversity was expressed by the Shannon–Wiener diversity index (*H’*), calculated from prey abundance composition based on the extended prey species list obtained from microscope analysis (i.e. 59 groups).

Beta-diversity (*β*_*W*_) for anchovy and for sardine was calculated using prey presence-absence data (considering each area separately), according to the following equation ^40^:

1 $$\beta_{w} = \frac{a + b + c}{{\left( {2a + b + c} \right)/2}} - 1$$

where component *a* comprises the total number of species that occur in both anchovy and sardine; component *b* comprises the total number of species that occur in anchovy but not in sardine; and component *c* comprises the total number of species that occur in sardine but not in anchovy. Regarding this pairwise comparison and assuming that the diet composition is similar for both species, a minimum value of 0 beta-diversity (similarity measures) would mean that both species share the niche equally, whereas a maximum value of 1 (dissimilarity measures) might be expected when one community dominates the ecosystem^39^.

The overlap in resource use between species (i.e. anchovy and sardine) and between areas was assessed using Pianka’s^41^ index of niche overlap:

2 $$O = \frac{{\sum p_{i,j} p_{i,k} }}{{\sqrt {\left[ {\left( {\sum p_{i,j}^{2} } \right)\left( {\sum p_{i,k}^{2} } \right)} \right]} }}$$

where *O* is the overlap index between the two species *j* and *k* expressed as a value between 0 and 1, where 0 means no overlap and 1 complete overlap in diets. *P*_*i,j*_ and *p*_*i,k*_ are the proportions of presence of the *i*^th^ prey group in the diets of species *j* and *k*, respectively. To assess the overlap index between areas (considering the species separately), ‘species’ were replaced with ‘areas’ in the same equation. For the diet overlap comparisons, the gut contents were categorized into the 11 prey groups mentioned before and used for graphical representations. To test for significance, the presence-proportion of a given prey group in a given diet was randomized according to the Randomization Algorithm (RA2) defined by Lawlor^87^ and iterated 1000 times for each comparison of diet overlap. Lawlor^87^ described four randomization algorithms (RA1-RA4) for niche overlap, in which the zero states (the empty prey groups) and the niche breadth (the degree of utilization of a prey group) can be either relaxed or retained. Under RA2, the zero states are retained (i.e. empty prey groups from the stomach samples remain empty in the simulations), while niche breadth is relaxed (i.e. the proportion in the diet of each non-empty prey group is replaced by a uniform value between 0 and 1). As in the case of the Bay of Biscay^43^, RA2 was considered to give the most realistic reflection of the Mediterranean Sea pelagic system because some of the prey groups would be unavailable to fish in certain areas, due to the patchy distribution of the plankton prey^88,89^, whereas none of the fish species were assumed to have constraints on the utilization of the prey groups that were actually present.

### DNA metabarcoding in gut contents

DNA was extracted from 143 gut content samples of anchovy and sardine analysed previously under the microscope (Table 6), using the NZY Tissue gDNA Isolation kit as per manufacturers protocol (NZYTech, Lisbon, Portugal). Prior to DNA extraction, vials were shaken by hand to homogenise the gut contents of each individual sampled fish. An extraction blank was included in every DNA extraction round and treated as a regular sample to check for cross-contamination. DNA was resuspended in a final volume of 100 μL.

#### Zooplankton and diatom characterization in diet

For library preparation of zooplankton, a 313 bp-fragment of the Cytochrome Oxidase subunit I (COI) region was amplified using the primers COIintF (5′ GGWACWGGWTGAACWGTWTAYCCYCC 3′) and COI dgHCO2198 (5′ TAAACTTCAGGGTGACCAAARAAYCA 3′)^90^, to which the Illumina sequencing primer sequences were attached to their 5′ ends. In order to limit the amplification of predator DNA, specific blocking primers were designed following Leray et al.^91^ using Geneious 11.1.5 (www.geneious.com), based on COI sequences of *Sardina* sp. and *Engraulis* sp. The blocking primers *dgHCO2198_engraulis* (with 5′–3′ sequence AAGAATCAGAATAGGTGTTGATAAAGAATC-C3) and *dgHCO2198_sardina* (with 5′–3′ sequence AAGAATCAGAATAGGTGCTGATACAGAATG-C3) were used during PCR in order to prevent amplification from anchovy and sardine, respectively. A C3 CPG spacer was added to the 3′ end of each blocking primer to prevent elongation.

Instead, for library preparation of diatoms, a fragment of around 312 bp of the rbcL chloroplast gene was amplified using the primers Diat_rbcL 708F_2 (5′ AGG TGA AGT TAA AGG TTC WTA YTT AAA 3′) and R3_1 (5′ CCT TCT AAT TTA CCW ACW ACT G 3′)^92^, to which the Illumina sequencing primer sequences were attached to their 5′ ends.

The libraries were constructed by PCR amplification of a region of the COI gene for zooplankton and a region of the rbcL gene for diatoms. PCRs were prepared in a volume of 25 μL, containing 1 μL of template DNA, 0.5 μM of the primers, 10 μM of the blocking primers, 6.25 μL of Supreme NZYTaq 2 × Green Master Mix (NZYTech, Lisbon, Portugal), and ultrapure water up to 25 μL. The thermal cycling profile included an initial denaturation at 95 °C for 5 min, followed by 35 cycles of 95 °C for 30 s, 53 °C for 30 s, 72 °C for 30 s, and a final extension step at 72 °C for 10 min. The same protocol but using only 5 cycles and 60 °C as the annealing temperature was used in a second PCR round to add index sequences for multiplexing different libraries in the same sequencing pool (see Fig. 1 in Vierna et al.^93^). The product sizes of both PCRs were checked by electrophoresis and visualised on 2% agarose gels. Then, COI and rbcL libraries were purified using the Mag-Bind RXNPure Plus magnetic beads (Omega Biotek, Norcross, USA), quantified using the Qubit dsDNA HS Assay (Thermo Fisher Scientific, Waltham, USA), and pooled equimolarly. Finally, the pool was sequenced in a MiSeq PE300 run (Illumina, San Diego, USA) at the facilities of Parque Científico de Madrid Foundation (Madrid, Spain).

The quality of the Illumina paired-end raw files was checked using the software FastQC (www.bioinformatics.babraham.ac.uk/projects/fastqc). Merging of paired-end reads was performed with FLASH2^94^. The mismatch resolution in the overlapping region (< 30 bp –base pairs–) was accomplished by keeping the base with the higher quality score. The CUTADAPT software 1.3^95^ was used to remove sequences that did not contain the PCR primers (allowing up to 2 mismatches) and sequences that ended up between 293 and 333 bp for the COI region and between 250 and 375 bp for the rbcL region. Sequences were quality-filtered (Phred score > 20) and labelled using the script multiple *split_libraries.py* implemented in Qiime v1.9.1^96^.

The resulting FASTA file was processed using the VSEARCH bioinformatic tool^97^. Sequences were dereplicated (-*derep full length*), clustered with the SWARM 2.0 algorithm^98^ with a *d* value of 13, and sorted (-*sortbysize*). Then, chimeras were de novo detected and removed using the UCHIME algorithm^99^ implemented in VSEARCH. Finally, sequences were assigned to Operational Taxonomic Units (OTUs) (-*usearch global*).

In the case of the zooplankton taxonomic identification, a custom reference database was created using an in-house developed script to process the information from the BOLD Public Data Portal (accessed on July 2019). All the COI sequences for the following metazoan groups were retrieved: Actinopterygii, Appendicularia, Ascidiacea, Branchiopoda, Ciliphora, Chaetognatha, Cnidaria, Hexanauplia, Malacostraca, Mollusca, Polychaeta and Thaliacea. The taxonomic identification was performed by querying the clustered centroids against our BOLD reference database using the script *assign_taxonomy.py* implemented in Qiime and the UCLUST algorithm^100^, with a sequence similarity threshold of 95%.

In the case of the diatoms taxonomic identification, it was performed by querying the sequences against the R-Sys reference database^92^. To do so, we used the naïve Bayesian method implemented in RDP^101^, with a confidence threshold of 80%.

OTUs occurring at a frequency below 0.005% in the whole dataset were removed^102^. In DNA metabarcoding studies it has been observed that a low percentage of the reads of a library can be assigned to another library. This phenomenon, referred to as *mistagging* is the result of the misassignment of the indices during library preparation, sequencing, and/or demultiplexing steps^103,104^. In order to correct for this phenomenon, the OTUs occurring at a frequency below 0.01% in each sample were removed.

OTUs that were not assigned to diatom taxa were removed from the final OTU table. In the case of zooplankton, OTUs without taxonomic assignment were queried directly against BOLD, using a similarity match of ≥ 95%. The OTUs that got a taxonomic assignment were included in the final OTU table. OTUs assigned to *Sardina* sp. and *Engraulis* sp. were removed from samples belonging to *Sardina pilchardus* and *Engraulis encrasicolus*, respectively. In the case of zooplankton, samples that ended up with < 20 sequences were also removed from the final OTU table.

Samples in the OTU table were sorted by sampling site, ontogenetic stage, and area. Then, the resulting OTU tables were converted into a Biological Observation Matrix files (.biom) for the posterior analysis of the data. DNA metabarcoding analyses were carried out by AllGenetics & Biology S.L. (www.allgenetics.eu).

Presence-absence information of prey (zooplankton) OTUs was used for diet characterization as well as to calculate the species richness, the beta-diversity for anchovy and for sardine (considering each area separately) and niche overlap, according to the same equations applied to gut content characterization data (see previous section).

The diatoms analysis was considered for diet characterization as well as for prey richness approach. In order to ease the interpretation of graphical representation, diatom groups within the 70^th^ percentile of occurrence frequencies (i.e. < 6.25) were merged as ‘Other diatom groups’. Diatoms without specific taxonomic information were presented as ‘Diatom remains’.

All the raw reads of prey species obtained from gut contents were deposited in the BioProject database (GenBank – NCBI^105^) as Bioproject PRJNA653773.

### Stable isotope analyses

Stable isotope analyses of *δ*^13^C and *δ*^15^N were performed in muscle tissue. A small portion of the dorsal muscle without skin was extracted from each fish sample. Then, tissue samples were oven-dried at 60ºC for 72 h. Dried samples were pulverized and 0.80 – 0.85 mg of muscle powder per fish was packed into tin capsules.

Isotopic analyses were performed at the Laboratory of Stable Isotopes of University of A Coruña, Galicia, Spain (Servicio de Analisis Instrumental (SAI)) through an elemental analyser (Carlo Erba CHNSO 1108) coupled to an isotopic ratio mass spectrometer (Finnigan Matt Delta Plus). The isotopic values are reported as *δ*^13^C (‰) and *δ*^15^N (‰) relative to the Vienna Pee Dee Belemnite or atmospheric nitrogen respectively^106^. USGS40 and L-alanine from the International Atomic Energy Agency were used, as well as internal acetanilide standards. The accuracy (± SE) of the standards replicates and samples for the two isotopes is < 0.1 and < 0.3% respectively. When the C:N ratio was greater than 3.5, it indicates that lipids are present in the sample^107^. Therefore, in these cases a correction was applied to the values of *δ*^13^C following the methodology of Post et al.^107^.

To provide insight into species’ trophic niche widths and to estimate the degree of isotopic niche overlap between the species, standard ellipse areas corrected for the sample size (SEA_C_; i.e. the area containing 40% of the data) as well as the Bayesian standard ellipse areas (SEA_B_^37^) were calculated for each species, ontogenetic stage (i.e. juveniles vs adults) and area (i.e. GSA07, GSA06-North, GSA06-South). This metric represents a measure of the core isotopic niche and higher values of SEA_C_ and SEA_B_ represent a broader trophic niche width^55^. Overlap between SEA_B_ were calculated as percentage of the area of species *A* overlapped with *B* and vice versa. SEA_B_ was computed using 10,000 posterior draws^37^.

### Generalized additive models

Generalized Additive Models (GAMs^108^) were implemented to test the influences of latitude, area (GSA07, GSA06-North, GSA06-South), depth (m), and total fish length (cm) on the SFD, Shannon diversity index, *δ*^13^C and *δ*^15^N of anchovy and sardine. Explanatory variables were tested for collinearity, correlation, outliers, and missing data, before using them in GAMs, following the procedure of Zuur et al.^109^. In particular, correlation among variables was checked by performing a Pearson’s correlation test. Collinearity was tested by computing the generalized variance-inflation factors (GVIF), which are the corrected VIF values by the number of degrees of freedom of a predictor variable^110^. In all cases variables had a correlation lower than 0.70 and a GVIF lower than 3 and consequently were used together in models (Figure S08).

GAMs are often used for their ability to deal with non-linear and non-monotonic relationships between the response variable and the explanatory variables^108,111^. Separated GAMs for each one of the response variable and species were performed. When response variable was not normally distributed a logarithmic transformation was applied; when the normality was not achieved after transformation, a Gamma distribution was used. Accordingly, for both, anchovy and sardine, the SFD was not normally distributed (Shapiro–Wilk normality tests; W = 0.90, *p* value < 0.001 and W = 0.83, *p* value < 0.001, respectively) and therefore a Gamma distribution with a log link was used. On the contrary the Shannon index was normally distributed (Shapiro–Wilk normality tests; W = 0.95, *p* value < 0.001 and W = 0.97, *p* value = 0.06 for anchovy and sardine, respectively) and a Gaussian distribution with the identity link was implemented. For *δ*^13^C a Gaussian distribution with a log-link was applied (Shapiro–Wilk normality tests; W = 0.98, *p* value = 0.40 and W = 0.98, *p* value = 0.10 for sardine and anchovy, respectively), as well as for *δ*^15^N (Shapiro–Wilk normality tests; W = 0.96, *p* value < 0.001 and W = 0.95, *p* value < 0.001 for sardine and anchovy, respectively).

Each GAM was fitted using thin plate regression splines for non-linear covariate and restricting the number of knots at 4 to avoid additional over-fitting. GAMs were performed with all possible combinations of terms. Variables were selected with forward and backward stepwise procedures based on three different criteria such as Akaike Information Criterion (AIC) and deviance explained. The best (and most parsimonious) model was finally chosen based on the compromise between low AIC values, high D (deviance explained) values, and significant predictors.

### Software

R software v.3.6.1^112^ was used for all analyses and graphical representations (except Fig. 1, see corresponding legend). For diet composition figures the package ‘ggplot2′ v.3.2.1^113^ was used. Diet overlap analyses in diet characterization data were performed using ‘EcoSimR’ package^114^. GAMs were performed using ‘mgcv’ package^115^, testing autocorrelation among variables and collinearity using ‘corrplot’ package^116^ and ‘corvif’ function, respectively. Isotopic SEAs and their overlap were calculated using SIBER package^37^. OTU table-based matrices were directly imported into R^112^.

### Ethical statement

Our study did not involve any endangered or protected species. No experimentation with animals was performed. No other ethical issues applied to the present research project. Special permissions or rules or sacrificing fish, from Institutional Animal Care and Use Committee (IACUC) or equivalent animal ethics committees, are at present non-existing in Spain for scientific fish sampling. Normally, the process of trawling and handling until biological sampling would lead to high mortality of the fish. Hence, fish were collected without unnecessary suffering.

## Supplementary information

Supplementary Information.
