# Supplementary material for: A trophic latitudinal gradient revealed in anchovy and sardine from the Western Mediterranean Sea using a multi-proxy approach
Source: Sci Rep. 2020 Oct 19;10:17598. doi: 10.1038/s41598-020-74602-y (PMC7572414; doi:10.1038/s41598-020-74602-y)
Supplement: Supplementary file 1 — Supplementary Information. [file 41598_2020_74602_MOESM1_ESM.docx]

**Supplementary Information**

**A trophic latitudinal gradient revealed in anchovy and sardine from the Western Mediterranean Sea using a multi-proxy approach**

**Eneko Bachiller^1,2,*^, Marta Albo-Puigserver^1^, Joan Giménez^1^, Maria Grazia Pennino^3^, Neus Marí-Mena^4^, Antonio Esteban^5^, Elena Lloret-Lloret^1^, Angelique Jadaud^6^, Belén Carro^4^, José María Bellido^5^, Marta Coll^1^**

^1^ Institut de Ciències del Mar (ICM-CSIC). Passeig Marítim de la Barceloneta, 37-49, 08003 Barcelona, Spain.

^2^ [Current address]: AZTI, Sustainable Fisheries Management (Data), Basque Research and Technology Alliance (BRTA). Txatxarramendi uhartea z/g, 48395 Sukarrieta, Bizkaia (Basque Country), Spain.

^3^ Instituto Español de Oceanografía, Centro Oceanográfico de Vigo. Subida Radio Faro, 50, 36390 Vigo, Spain.

^4^ AllGenetics & Biology SL. Edificio CICA. Campus de Elviña, 15008 A Coruña, Spain.

^5^ Instituto Español de Oceanografía, Centro Oceanográfico de Murcia. Varadero 1 Apdo 22, 30740 San Pedro del Pinatar, Murcia, Spain.

^6^ Marine Biodiversity, Exploitation and Conservation (MARBEC), Ifremer, Univ Montpellier, CNRS, IRD. Sète, France.

^*^ [Corresponding author] ebachiller@mail.com

1. **anchovy**


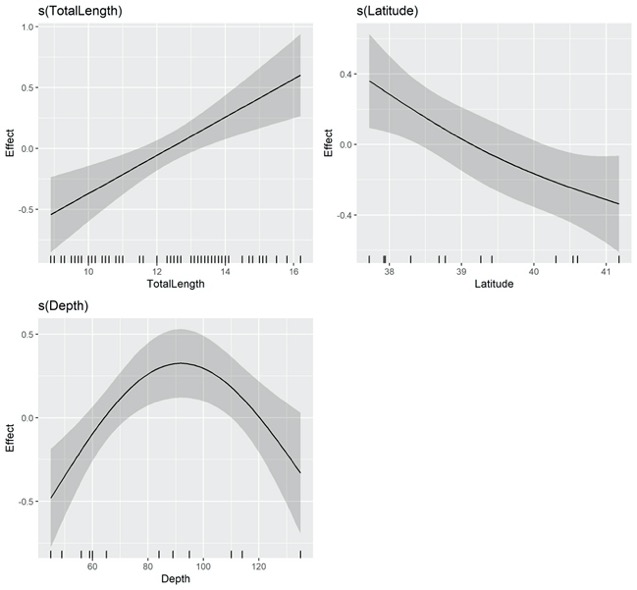


**b) sardine**

**
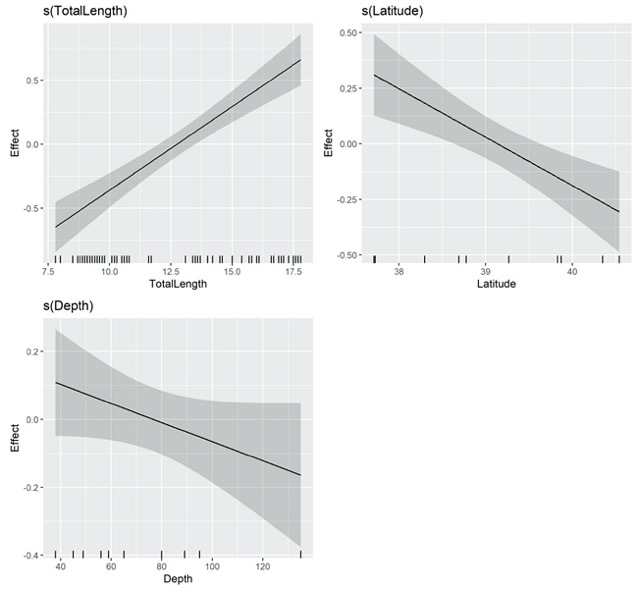
**

**Figure S01.** Partial GAM plots for the SFD Gamma models for **(a)** anchovy and **(b)** sardine. Each

plot represents the response variable shape, independent of the other variables, in relation to the probability of the species SFD in the multivariate model. The ranges of explicative variables are represented on the x-axis, while the y-axis is a relative scale where the effect of different values of the predictors on the response variable is shown. Confidence intervals (95%) around the response curve are shaded in grey.

**
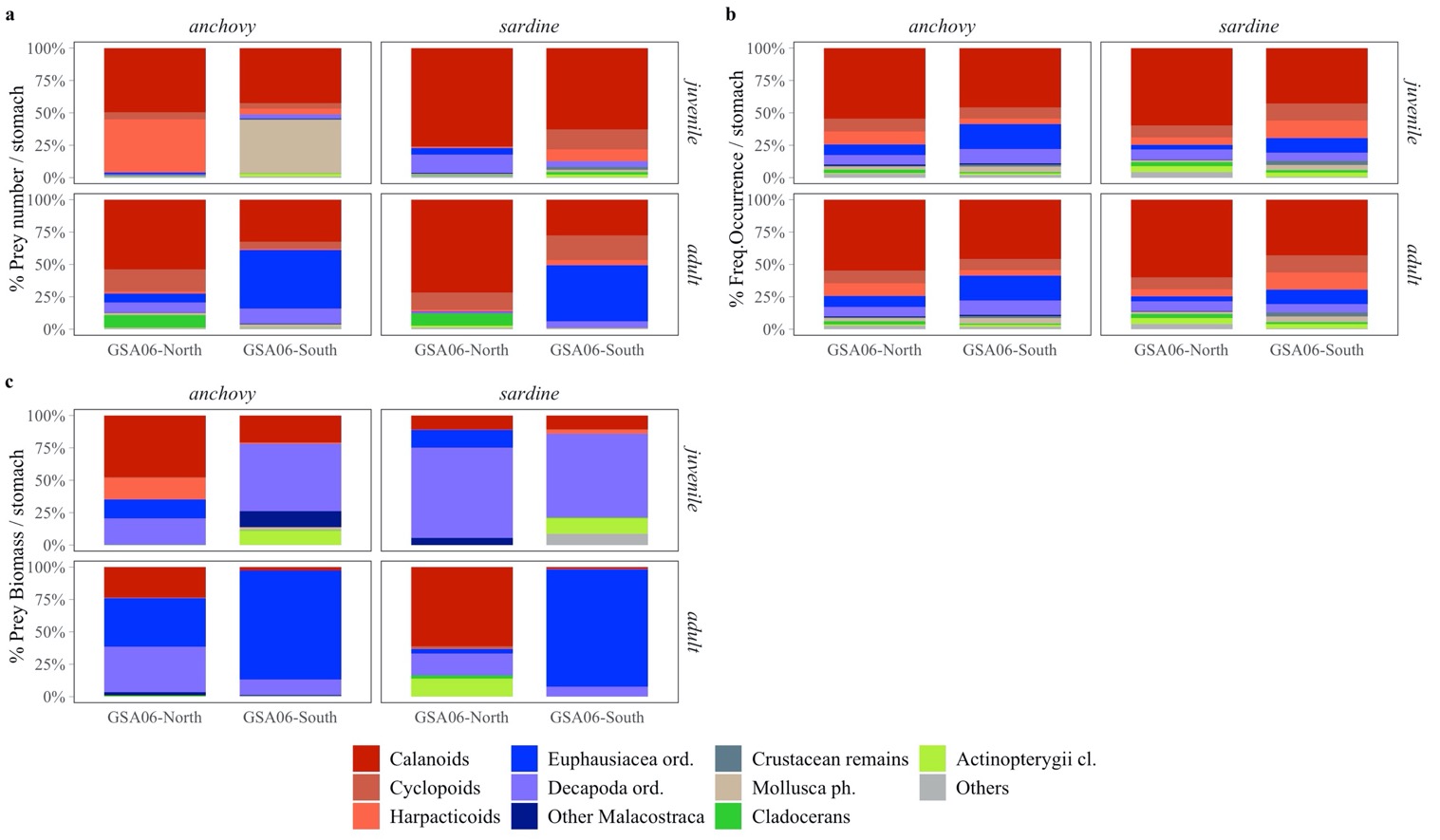
**

**Figure S02.** Prey group composition as percentage of **(a)** mean prey abundance, **(b)** occurrence and **(c)** mean prey biomass per stomach, identified under the microscope for anchovy and sardine, averaged over stages (juveniles vs. adults) and areas (data only available in GSA06-North and GSA06-South).

**Figure S03.** Carbon δ^13^C and nitrogen δ^15^N stable isotope boxplot of anchovy and sardine for each sampling area.

1. **anchovy**


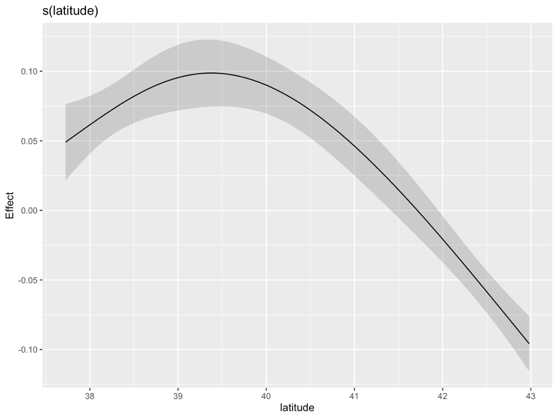


1. **sardine**


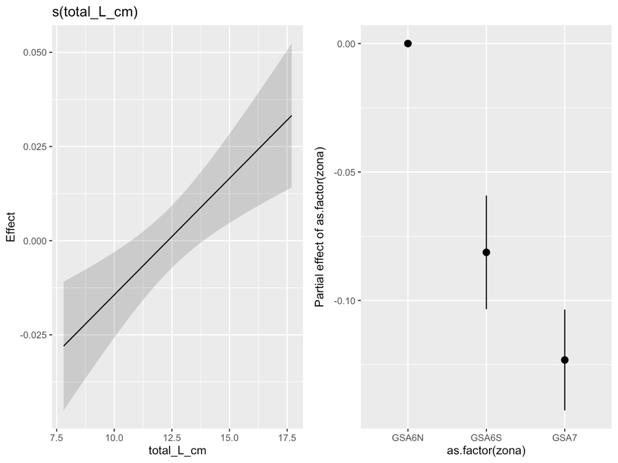


**Figure S04.** Partial GAM plots for δ^15^N GAM models for **(a)** anchovy and **(b)** sardine. Each plot represents the response variable shape, independent of the other variables, in relation to the probability of the species δ^15^N in the multivariate model. The ranges of explicative variables are represented on the x-axis, while the y-axis is a relative scale where the effect of different values of the predictors on the response variable is shown. Confidence intervals (95%) around the response curve are shaded in grey.

1. **anchovy**


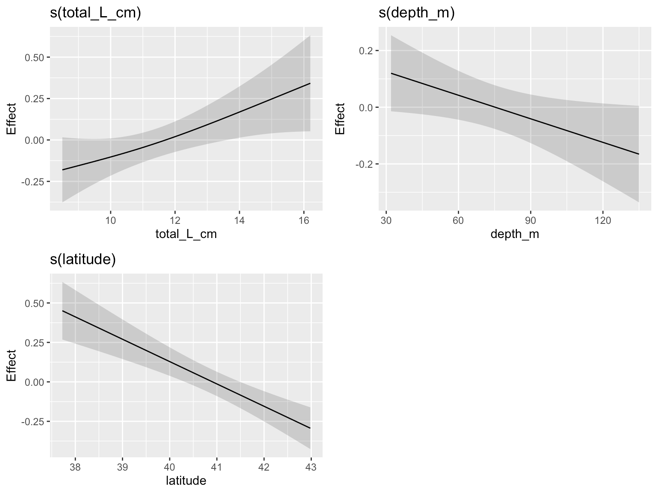


1. **sardine**


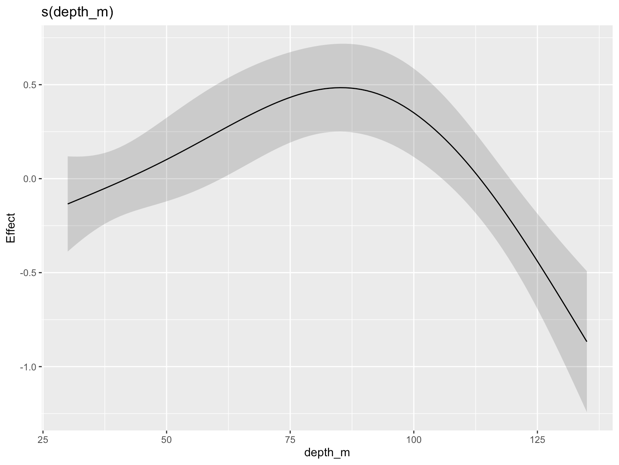


**Figure S05.** Partial GAM plots for δ^13^C GAM models for **(a)** anchovy and **(b)** sardine. Each plot represents the response variable shape, independent of the other variables, in relation to the probability of the species δ^13^C in the multivariate model. The ranges of explicative variables are represented on the x-axis, while the y-axis is a relative scale where the effect of different values of the predictors on the response variable is shown. Confidence intervals (95%) around the response curve are shaded in grey.


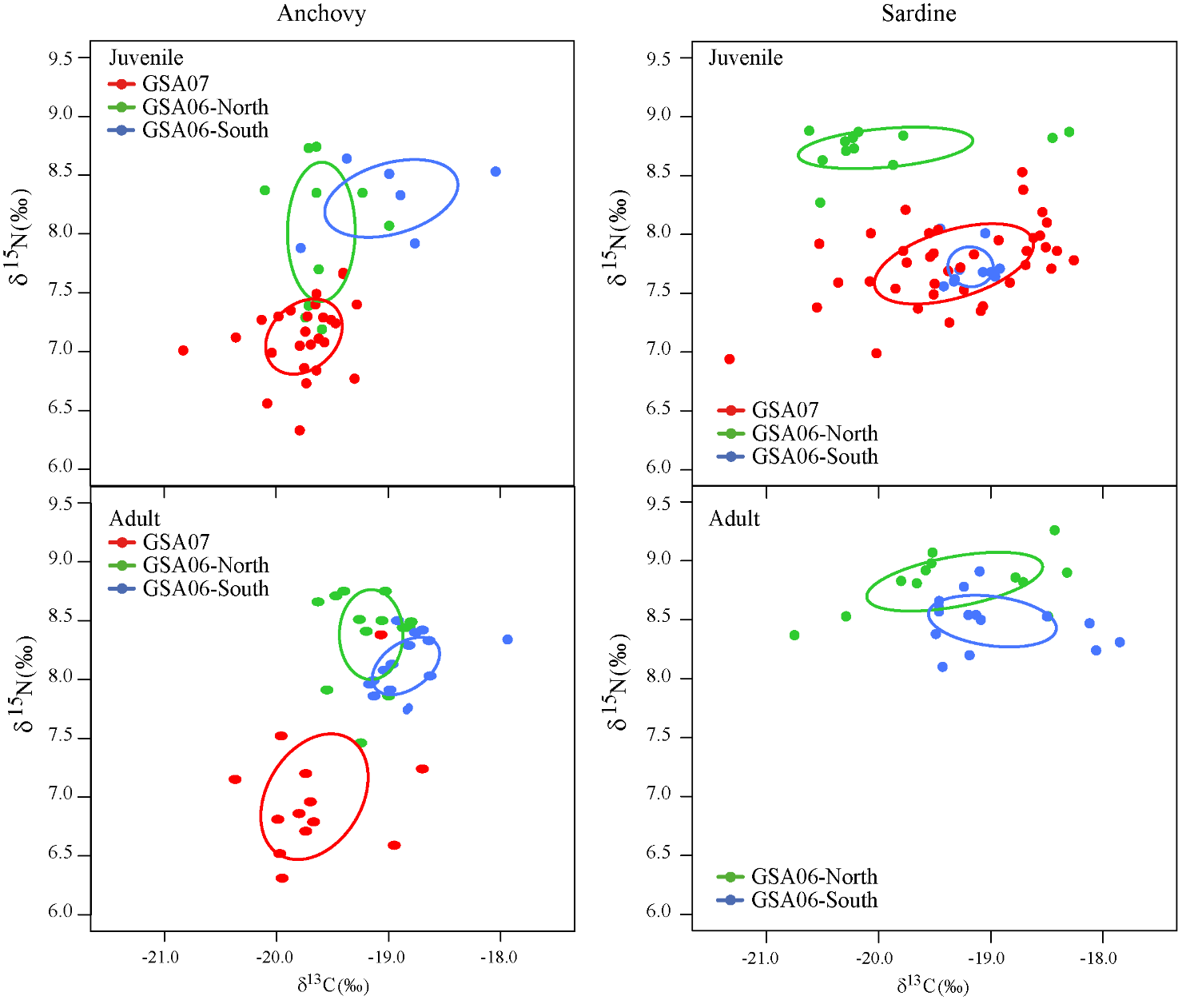


**Figure S06.** Corrected standard ellipses areas (SEA_C_) in anchovy and sardine juveniles and adults, measured for each area. Individual *δ*^13^C and *δ*^15^N values of GSA07 (red dots), GSA06-North (green dots) and GSA06-South (blue dots) are also graphed. In the case of GSA07 no sardine adult was available.

**a) anchovy**

**
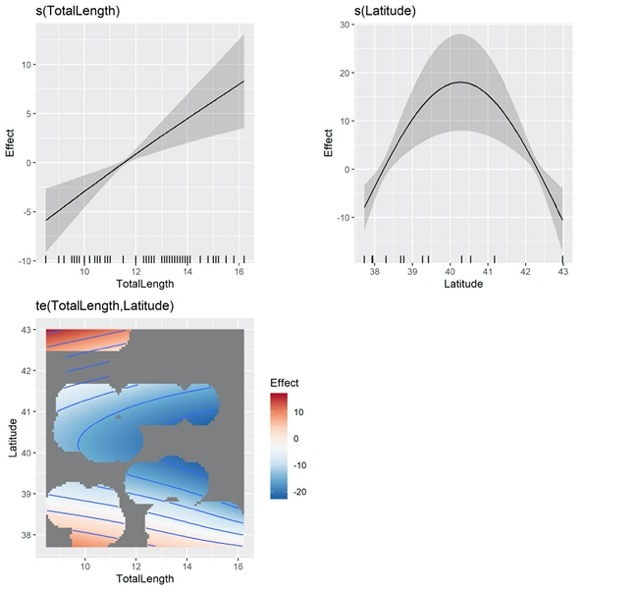
**

**b) sardine**


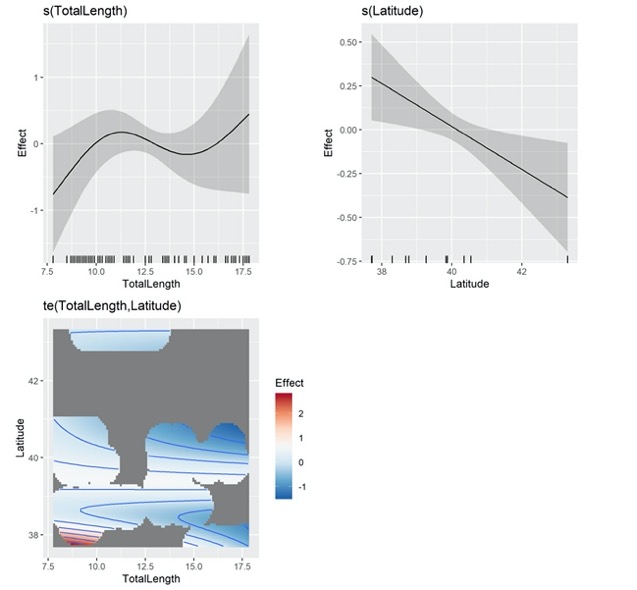


**Figure S07.** Partial GAM plots for the Shannon diversity index GAM models for **(a)** anchovy and **(b)** sardine. Each plot represents the response variable shape, independent of the other variables, in relation to the probability of the species Shannon index in the multivariate model. The ranges of explicative variables are represented on the x-axis, while the y-axis is a relative scale where the effect of different values of the predictors on the response variable is shown. Confidence intervals (95%) around the response curve are shaded in grey.

**a**

|  |  | D^2^ % | AIC | |
| --- | --- | --- | --- | --- |
| **anchovy** | |  |  | |
| **Model 1** | **1*+D*+Lat*+TL*** | **66.3** | **-405.66** | |
| Model 2 | 1*+D*+Lat*+TL*+A | 81.7 | -405.57 | |
| Model 3 | 1*+Lat*+TL* | 61.1 | -364.36 | |
| Model 4 | 1*+D*+Lat* | 59.4 | -361.95 | |
| Model 5 | 1*+D*+TL* | 58.4 | -362.03 | |
| **sardine** | |  | |  |
| **Model 1** | **1*+D*+Lat*+TL*** | **76.2** | **-372.86** | |
| Model 2 | 1*+D*+Lat*+TL*+A | 80.0 | -403.61 | |
| Model 3 | 1*+D*+TL* | 68.7 | -376.84 | |
| Model 4 | 1*+D*+Lat* | 69.0 | -377.21 | |
| Model 5 | 1*+D*+TL* | 70.0 | -392.21 | |

**b**

|  | **edf** | **Ref.edf** | **F** | **p-value** |
| --- | --- | --- | --- | --- |
| **anchovy** |  |  |  |  |
| TL | 1.00 | 1.00 | 14.62 | 3.15e-04 |
| Lat | 1.32 | 1.54 | 5.86 | 7.29e-03 |
| D | 1.94 | 1.99 | 7.67 | 10.23e-04 |
| **sardine** | | | | |
| TL | 1 | 1 | 55.79 | 9.41e-11 |
| Lat | 1 | 1 | 15.46 | 2.13e-04 |
| D | 1 | 1 | 2.96 | 9.04e-02 |

**Table S01.** **(a)** Comparison of the most relevant GAMs tested for SFD of anchovy and sardine. Explanatory variable acronyms are: D = depth (m), Lat = latitude, TL = total length of fish (cm), A = area. Statistics acronyms are: D^2^ = deviance explained, AIC = Akaike Information Criterion. Significant predictors are indicated with ‘*’. The best SFD GAM model is highlighted in bold. **(b)** Numerical summary of the best SFD GAM obtained for anchovy and sardine. Statistics acronyms are: edf = degrees of freedom, Ref.edf = relative degrees of freedom, F = F statistic.

| ***Merged group*** | ***Prey species/group (microscope)*** | ***Prey species/group***  ***(DNA metabarcoding)*** | ***Wet weight (mg)*** |
| --- | --- | --- | --- |
| Calanoids | Calanoid naupli | Calanoida ord. | 0.004 |
|  | Calanoid copepodite | Calanoida ord. | 0.036 |
|  | Calanoid copepodite* | *Nannocalanus minor* | 0.036 |
|  | Unidentified Calanoid | Calanoida ord. | 0.224 |
|  | *Acartia* spp. | *Acartia clausi, A. discaudata* | 0.019 |
|  | *Calanus gracilis* | *Pleuromamma gracilis* | 0.318 |
|  | *Calanus helgolandicus* | *Calanus helgolandicus* | 0.318 |
|  | *Calanus helgolandicus** | *Calanus euxinus, Mesocalanus tenuicornis* | 0.318 |
|  | *Calocalanus tenuis** | *Calocalanus contractus, C. styliremis, Mecynocera clausi, Rhincalanus nasutus, Subeucalanus pileatus* | 0.857 |
|  | *Candacia armata* | *Candacia armata* | 0.773 |
|  | *Candacia armata** | *Paraeuchaeta* spp. | 0.773 |
|  | *Centropages* spp. | *Centropages typicus, Bradyidius armatus* | 0.219 |
|  | *Centropages typicus, C. chierchiae** | *Centropages typicus* | 0.219 |
|  | Clauso-/Para-/Calanidae fam. | *Paracalanus parvus, Mesaiokeras hurei, Scolecithricella dentata* | 0.318 |
|  | *Clausocalanus* spp. | *Clausocalanus arcuicornis, C. jobei, C. lividus, C. parapergens, C. paululus, C. pergens* | 0.736 |
|  | *Diaixis hibernica,* Furcilia larvae* | *Diaixis hibernica* | 0.318 |
|  | *Paraeuchaeta* spp. | *Paraeuchaeta* spp. | 0.996 |
|  | *Pleuromamma abdominalis** | *Pleuromamma borealis, P. gracilis* | 0.318 |
|  | *Temora* spp.*, T. stylifera* | *Temora stylifera* | 0.246 |
|  |  |  |  |
| Cyclopoids | Cyclopoid naupli | Poecilostomatoida ord. | 0.004 |
|  | Cyclopoid copepodite | Poecilostomatoida ord. | 0.032 |
|  | Unidentified Cyclopoid | Poecilostomatoida ord. | 0.028 |
|  | Unidentified Cyclopoid* | *Sapphirina angusta* | 0.028 |
|  | *Corycaeus anglicus* | *Ditrichocorycaeus anglicus* | 0.072 |
|  | *Oithona* spp.* | *Lucicutia flavicornis* | 0.028 |
|  | *Oncaea* spp. | *Oncaea mediterranea, O. scottodicarloi, O. venusta* | 0.016 |
|  | *Oncaea* spp.* | *Triconia dentipes* | 0.016 |
|  |  |  |  |
| Harpacticoids | Harpacticoid naupli | Harpacticoida ord. | 0.004 |
|  | Harpacticoid copepodite | Harpacticoida ord. | 0.034 |
|  | Harpacticoid copepodite* | *Longipedia* sp. | 0.034 |
|  | *Euterpina acutifrons* | *Euterpina acutifrons* | 0.031 |
|  | *Euterpina acutifrons** | *Pseudameira* sp. | 0.031 |
|  | *Microsetella rosea* | *Microsetella* spp. | 0.137 |
|  | *Microsetella* spp. | *Microsetella norvegica* | 0.137 |
|  |  |  |  |
| Euphausiacea ord. | Metanauplius | Euphausiacea ord. | 0.005 |
|  | Calyptopis larvae | Euphausiacea ord. | 5.621 |
|  | Calyptopis larvae* | *Pseudocuma similis* | - |
|  | Furcilia larvae | Euphausiacea ord. | 5.621 |
|  | Unidentified Euphausiid | Euphausiacea ord. | 11.795 |
|  | *Meganyctiphanes norvegica* | *Meganyctiphanes norvegica* | 11.795 |
|  | *Meganyctiphanes norvegica** | *Euphausia krohni* | 11.795 |
|  | *Nyctiphanes couchi** | *Nematoscelis megalops* | 11.795 |
|  |  |  |  |
| Decapoda ord. | Decapod Zoea larvae | Decapoda ord. | 3.284 |
|  | Decapod Megalopa larvae | *Derilambrus angulifrons, Ebalia cranchii, Eurynome spinosa, Goneplax rhomboides, Inachus communissimus, I. dorsettensis, Liocarcinus depurator, L. maculatus, L. vernalis, L. zariquieyi, Pagurus prideaux* | 3.284 |
|  | Decapod Late Larvae | *Anapagurus breviaculeatus, A. chiroacanthus, A. laevis, Eualus cranchii, Eusergestes arcticus, Pagurus forbesii, Pandalina brevirostris, Philocheras bispinosus, Processa edulis* | 5.621 |
|  | Unidentified Decapod | Decapoda ord. | 8.747 |
|  | Unidentified Decapod | *Chlorotocus crassicornis, Jaxea nocturna, Pasiphaea sivado, Processa modica, P. nouveli, Solenocera membranacea* | 8.747 |
|  |  |  |  |
| Other Malacostraca | Unidentified Amphipod | *Ampelisca brevicornis, A. typica, Phrosina semilunata, Vibilia armata* | 18.935 |
|  | Unidentified Malacostraca | Malacostraca cl. | 11.795 |
|  | Unidentified Mysid | Malacostraca cl. | 11.795 |
|  | Unidentified Isopod* | *Rissoides desmaresti* | 18.935 |
|  |  |  |  |
| Crustacean remains | Cirripedia naupli* | *Copilia quadrata* | 0.028 |
|  | Cirripedia | Cirripedia infracl. | 0.029 |
|  | Monstrilla spp.* | - | 0.318 |
|  | Siphonostomatoida ord. | Siphonostomatoida ord. | 0.028 |
|  |  |  |  |
| Mollusca ph. | Bivalve veliger | *Abra nitida, Archiconchoecia striata, Corbula gibba, Porroecia spinirostris, Proceroecia microprocera, Spisula subtruncata* | 0.012 |
|  | Gastropod veliger (Prosobranchia) | Gastropoda cl. | 0.031 |
|  | Gastropod veliger (Prosobranchia) | *Aporrhais pespelecani, Euspira nitida, Limacina inflata, Pusillina inconspicua* | 0.031 |
|  | Gastropod veliger (Prosobranchia)* | Cavoliniidae fam. | 0.031 |
|  |  |  |  |
| Cladocerans | *Penilia avirostris* | *Penilia avirostris* | 0.087 |
|  | *Penilia avirostris** | *Evadne nordmanni* | 0.087 |
|  | *Podon* spp. | *Podon intermedius* | 0.087 |
|  | *Podon* spp.* | *Pleopis polyphemoides* | 0.087 |
|  |  |  |  |
| Actinopterygii cl. | Fish egg: *E. encrasicolus* | - | 3.066 |
|  | Fish egg: Unidentified fish | *Echiodon drummondii, Sciaena umbra, Lesueurigobius friesii, Trisopterus capelanus, Salmo salar, Mullus barbatus, Spicara maena, Crystallogobius linearis, Gymnammodytes cicerelus, Deltentosteus quadrimaculatus, Merluccius merluccius, Sprattus sprattus,* Stomiiformes ord.*, Pomatoschistus pictus* | 2.132 |
|  | Larvae: Unidentified fish |  | 7.648 |
|  |  |  |  |
| Others | Crustacean egg | *-* | *-* |
|  | Hydrozoa unidentified | *Muggiaea atlantica* | 4.014 |
|  | Insect larva | *-* | *-* |
|  |  |  |  |

**Table S02.** Definition of prey groups identified in stomach contents of anchovy and sardine under the microscope, with DNA metabarcoding, the assigned wet weight (based on weight-length conversion equations in Bachiller & Irigoien^1^), and (merged) classification of groups for graphical presentation.

* denotes uncertainty in the identification and/or group assignment.

| ***Merged group*** | ***Prey species/group (DNA metabarcoding)*** | ***Wet weight (mg)*** |
| --- | --- | --- |
| Annelida ph. | *Chaetopterus variopedatus, Clymenura clypeata, Labioleanira yhleni, Magelona* sp.*, Pectinaria koreni,* Phyllodocida *ord., Phyllodoce rosea, Scolaricia sp.,* Terebellida ord. | 0.03 |
| Chaetognatha ph. | *Pseudosagitta lyra* | 0.02 |
| Cnidaria ph. | Actiniaria ord. (Anthozoa cl.)  Hydrozoa cl.: *Abylopsis tetragona, Aglaura hemistoma, Bougainvillia muscus, Clytia hemisphaerica, Corymorpha sp., Euphysa aurata, Helgicirrha cari, Leuckartiara octona, Lizzia blondina, Muggiaea atlantica, Nanomia bijuga, Obelia dichotoma, Podocoryna areolata, Sphaeronectes gracilis, Stauridiosarsia gemmifera*  Scyphozoa cl. | 3.93 |
| Echinodermata ph. | *Amphiura filiformis, Gracilechinus acutus, Luidia sarsi, Ophiocomina nigra, Ophiura grubei, O. ophiura, Paracentrotus lividus* | 0.08 |
| Haptophyta ph. | *Coccolithus* sp. | - |
| Mollusca ph. |  |  |
| Nemertea ph. | Tubulanidae fam. | - |
| Porifera ph. | Porifera ph. | - |

**Table S03.** Definition of prey groups within ‘Others’ group identified in stomach contents of anchovy and sardine with DNA metabarcoding, the assigned wet weight (based on weight-length conversion equations in Bachiller & Irigoien^1^), and merged phylum groups for graphical presentation.

| ***Family*** | ***Prey species/group***  ***(DNA metabarcoding)*** | ***N_E.enc_ (%)*** | | ***N_S.pil_ (%)*** | | ***Harmful*** | ***Rarity*** |
| --- | --- | --- | --- | --- | --- | --- | --- |
| Bacillariaceae | Bacillariaceae fam. (und.sp.) | 16 | (21.92) | 34 | (48.57) |  |  |
|  | *Cylindrotheca closterium* | 8 | (10.96) | 33 | (47.14) | *^2^ | F |
|  | *Nitzschia* sp. | 8 | (10.96) | 20 | (28.57) | *^3^ | VR |
|  | *Psammodictyon constrictum* | 13 | (17.81) | 19 | (27.14) |  |  |
|  | *Pseudo-nitzschia americana* | 14 | (19.18) | 36 | (51.43) | *^3^ |  |
|  | *Pseudo-nitzschia calliantha* | 14 | (19.18) | 36 | (51.43) | *^3^ | F |
|  | *Pseudo-nitzschia delicatissima* | 52 | (71.23) | 55 | (78.57) | *^3^ | F |
|  | *Pseudo-nitzschia galaxiae* | 39 | (53.42) | 40 | (57.14) | *^3^ | F |
|  | *Pseudo-nitzschia multiseries* | 6 | (8.22) | 31 | (44.29) | *^3^ |  |
|  | *Pseudo-nitzschia multistriata* | 5 | (6.85) | 13 | (18.57) | *^3^ | VR |
|  | *Pseudo-nitzschia* sp. | 18 | (24.66) | 30 | (42.86) | *^3^ |  |
| Bolidomonadaceae | *Bolidomonas pacifica* | 11 | (15.07) | 15 | (21.43) |  |  |
| Catenulaceae | *Amphora helenensis* | 1 | (1.37) | 5 | (7.14) |  |  |
| Chaetocerotaceae | Chaetocerotaceae fam. | 16 | (21.92) | 33 | (47.14) |  |  |
|  | *Chaetoceros danicus* | 13 | (17.81) | 35 | (50) | *^4^ | VR |
|  | *Chaetoceros didymus* | 7 | (9.59) | 25 | (35.71) |  |  |
|  | *Chaetoceros socialis* | 29 | (39.73) | 43 | (61.43) | *^2^ |  |
| Corethraceae | *Corethron hystrix* | 0 | (0) | 3 | (4.29) |  | R |
| Coscinodiscaceae | *Coscinodiscus wailesii* | 5 | (6.85) | 23 | (32.86) | *^5^ | VR |
| Cymatosiraceae | *Arcocellulus mammifer* | 18 | (24.66) | 10 | (14.29) |  |  |
|  | *Papiliocellulus simplex* | 34 | (46.58) | 48 | (68.57) |  | ER |
| Entomoneidaceae | Entomoneidaceae fam. | 9 | (12.33) | 12 | (17.14) |  |  |
| Fragilariaceae | *Asterionellopsis guyunusae* | 10 | (13.7) | 17 | (24.29) |  |  |
| Grammatophoraceae | Grammatophoraceae fam. | 1 | (1.37) | 3 | (4.29) |  |  |
| Hemiaulaceae | *Cerataulina pelagica* | 29 | (39.73) | 55 | (78.57) | *^2^ | F |
|  | *Eucampia cornuta* | 17 | (23.29) | 24 | (34.29) |  | VR |
|  | *Hemiaulus sinensis* | 6 | (8.22) | 28 | (40) |  |  |
| Lauderiaceae | *Lauderia annulata* | 7 | (9.59) | 25 | (35.71) |  |  |
| Leptocylindraceae | *Leptocylindrus danicus* | 30 | (41.1) | 43 | (61.43) |  | A |
| Licmophoraceae | *Licmophora abbreviata* | 1 | (1.37) | 1 | (1.43) |  |  |
| Melosiraceae | *Melosira varians* | 13 | (17.81) | 3 | (4.29) |  |  |
| Naviculaceae | Naviculaceae.fam | 7 | (9.59) | 27 | (38.57) |  | ER |
| Paraliaceae | *Paralia sulcata* | 2 | (2.74) | 3 | (4.29) |  |  |
| Pleurosigmataceae | Pleurosigmataceae fam. | 23 | (31.51) | 41 | (58.57) |  | VR |
| Rhizosoleniaceae | Rhizosoleniaceae fam. | 7 | (9.59) | 26 | (37.14) |  |  |
|  | *Guinardia striata* | 17 | (23.29) | 28 | (40) |  | F |
|  | *Pseudosolenia calcar-avis* | 17 | (23.29) | 32 | (45.71) |  |  |
|  | *Rhizosolenia fallax* | 38 | (52.05) | 59 | (84.29) |  |  |
|  | *Rhizosolenia shrubsolei* | 38 | (52.05) | 59 | (84.29) |  |  |
| Skeletonemataceae | Skeletonemataceae fam. | 43 | (58.9) | 40 | (57.14) |  |  |
|  | *Skeletonema potamos* | 6 | (8.22) | 10 | (14.29) |  |  |
| Surirellaceae | Surirellaceae fam. | 1 | (1.37) | 3 | (4.29) |  |  |
| Thalassionemataceae | *Thalassionema frauenfeldii* | 5 | (6.85) | 12 | (17.14) |  | F |
| Thalassiosiraceae.fam | Thalassiosiraceae fam. | 43 | (58.9) | 48 | (68.57) |  |  |
|  | *Minidiscus trioculatus* | 56 | (76.71) | 49 | (70) |  | ER |
|  | *Planktoniella sol* | 10 | (13.7) | 8 | (11.43) |  |  |
|  | *Thalassiosira mediterranea* | 21 | (28.77) | 15 | (21.43) |  |  |
|  | *Thalassiosira oceanica* | 13 | (17.81) | 19 | (27.14) |  | ER |
|  | *Thalassiosira oestrupii* | 0 | (0) | 7 | (10) |  |  |
|  | *Thalassiosira pseudonana* | 2 | (2.74) | 4 | (5.71) |  |  |
| Triceratiaceae.fam | Triceratiaceae fam. | 2 | (2.74) | 2 | (2.86) |  |  |
|  | *Odontella mobiliensis* | 0 | (0) | 14 | (20) |  |  |

**Table S04.** Diatom (Bacillariophyta ph.) families and species determined in stomach contents of anchovy and sardine with DNA metabarcoding. ‘und.sp’ means undetermined species within the indicated taxonomic group. *N_E.enc_* and *N_S.pil_* denote the number of anchovy and sardine gut samples (and the percentage of samples, %) where the corresponding species (or diatom group) was found, respectively. ‘Harmful’ column indicates the references that classified the corresponding taxa as toxic or potentially harmful algae for fish; and the ‘Rarity’ indicates the classification depending on the degree of distribution in the Mediterranean Sea according to Percopo et al.^6^, defined as: A, abundant (>10^5^ cells l^-1^); F, frequent (10^4^–10^5^ cells l^-1^); R, rare (<10^4^ cells l^-1^); VR, very rare (<5x10^3^ cells l^-1^); ER, extremely rare, only observed in electron microscopy or serial dilution culture.

**a) anchovy**

|  | GSA07 | | |  | GSA06-North | | | | | |  | | GSA06-South | | | | | |
| --- | --- | --- | --- | --- | --- | --- | --- | --- | --- | --- | --- | --- | --- | --- | --- | --- | --- | --- |
|  | %ABD | %BIO | %FO |  | %ABD | | %BIO | | %FO | |  | %ABD | | | %BIO | | %FO | |
| Prey group (species)⇓ stage⇒ | juv | juv | juv |  | juv | ad | juv | ad | juv | ad |  | juv | | ad | juv | ad | juv | ad |
| *Acartia* spp. | 19.83 | 0.25 | 5.56 |  | 2.32 | 1.70 | 0.14 | 0.02 | 4.17 | 4.12 |  | 1.61 | | 1.86 | 0.08 | 0.01 | 4.00 | 3.80 |
| Bivalve veliger | 4.74 | 0.04 | 5.56 |  | 0.39 | 1.38 | 0.01 | 0.01 | 1.39 | 2.35 |  | 16.13 | | 0.17 | 0.49 | 0.00 | 4.00 | 0.63 |
| Calanoid copepodite | 16.81 | 0.15 | 12.70 |  | 6.56 | 9.14 | 0.27 | 0.08 | 11.11 | 5.88 |  | 12.90 | | 3.05 | 0.45 | 0.01 | 10.00 | 5.70 |
| Calanoid naupli | 2.16 | 0.01 | 3.17 |  | 0.00 | 0.53 | 0.00 | 0.00 | 0.00 | 1.76 |  | 11.29 | | 0.34 | 0.12 | 0.00 | 6.00 | 0.63 |
| *Calanus gracilis* | 0.00 | 0.00 | 0.00 |  | 0.00 | 0.00 | 0.00 | 0.00 | 0.00 | 0.00 |  | 0.00 | | 0.00 | 0.00 | 0.00 | 0.00 | 0.00 |
| *Calanus helgolandicus* | 0.43 | 0.09 | 0.79 |  | 3.09 | 2.34 | 2.97 | 0.46 | 6.94 | 3.53 |  | 0.81 | | 5.25 | 0.67 | 0.29 | 4.00 | 5.70 |
| *Calocalanus tenuis* | 0.00 | 0.00 | 0.00 |  | 0.00 | 0.64 | 0.00 | 0.34 | 0.00 | 1.76 |  | 0.40 | | 0.85 | 0.90 | 0.12 | 2.00 | 0.63 |
| Calyptopis larvae | 0.86 | 3.09 | 1.59 |  | 0.77 | 1.06 | 13.14 | 3.71 | 2.78 | 4.71 |  | 0.00 | | 0.34 | 0.00 | 0.33 | 0.00 | 1.27 |
| *Candacia armata* | 0.00 | 0.00 | 0.00 |  | 3.09 | 17.32 | 16.54 | 19.01 | 2.78 | 6.47 |  | 1.61 | | 5.08 | 7.40 | 1.54 | 4.00 | 3.16 |
| *Centropages chierchiae* | 0.00 | 0.00 | 0.00 |  | 0.39 | 0.00 | 0.16 | 0.00 | 1.39 | 0.00 |  | 0.00 | | 0.00 | 0.00 | 0.00 | 0.00 | 0.00 |
| *Centropages* spp. | 0.00 | 0.00 | 0.00 |  | 1.54 | 1.28 | 0.64 | 0.11 | 2.78 | 2.94 |  | 1.21 | | 0.17 | 0.43 | 0.00 | 2.00 | 0.63 |
| *Centropages typicus* | 3.88 | 0.34 | 3.97 |  | 4.25 | 1.49 | 1.75 | 0.13 | 5.56 | 3.53 |  | 0.00 | | 0.34 | 0.00 | 0.01 | 0.00 | 1.27 |
| Cirripedia | 0.00 | 0.00 | 0.00 |  | 0.39 | 0.00 | 0.03 | 0.00 | 1.39 | 0.00 |  | 0.40 | | 0.00 | 0.03 | 0.00 | 2.00 | 0.00 |
| Cirripedia naupli | 0.00 | 0.00 | 0.00 |  | 0.00 | 0.00 | 0.00 | 0.00 | 0.00 | 0.00 |  | 0.00 | | 0.00 | 0.00 | 0.00 | 0.00 | 0.00 |
| Clauso-/Para-calanidae fam. | 2.16 | 0.44 | 3.17 |  | 4.25 | 4.14 | 4.09 | 0.82 | 6.94 | 5.29 |  | 1.61 | | 0.34 | 1.33 | 0.02 | 6.00 | 1.27 |
| *Clausocalanus* sp. | 0.00 | 0.00 | 0.00 |  | 2.70 | 1.28 | 6.03 | 0.58 | 2.78 | 3.53 |  | 2.02 | | 2.03 | 3.85 | 0.26 | 4.00 | 4.43 |
| *Clytemnestra* spp. | 0.00 | 0.00 | 0.00 |  | 0.00 | 0.11 | 0.00 | 0.01 | 0.00 | 0.59 |  | 0.00 | | 0.00 | 0.00 | 0.00 | 0.00 | 0.00 |
| *Corycaeus anglicus* | 2.16 | 0.10 | 1.59 |  | 1.54 | 5.63 | 0.34 | 0.25 | 5.56 | 4.71 |  | 0.00 | | 0.17 | 0.00 | 0.00 | 0.00 | 0.63 |
| Cyclopoid copepodite | 0.00 | 0.00 | 0.00 |  | 0.00 | 0.00 | 0.00 | 0.00 | 0.00 | 0.00 |  | 0.00 | | 0.34 | 0.00 | 0.00 | 0.00 | 0.63 |
| Cyclopoid.naupli | 0.00 | 0.00 | 0.00 |  | 0.00 | 0.00 | 0.00 | 0.00 | 0.00 | 0.00 |  | 2.82 | | 0.00 | 0.03 | 0.00 | 2.00 | 0.00 |
| Decapod late larvae | 2.59 | 9.28 | 3.17 |  | 0.00 | 1.91 | 0.00 | 6.67 | 0.00 | 4.12 |  | 1.21 | | 6.44 | 17.64 | 6.20 | 4.00 | 5.70 |
| Decapod megalopa larvae | 0.00 | 0.00 | 0.00 |  | 0.00 | 0.53 | 0.00 | 1.08 | 0.00 | 1.18 |  | 0.00 | | 1.53 | 0.00 | 0.86 | 0.00 | 1.27 |
| Decapod zoea larvae | 0.00 | 0.00 | 0.00 |  | 0.00 | 0.53 | 0.00 | 1.08 | 0.00 | 1.76 |  | 0.81 | | 0.85 | 6.87 | 0.48 | 2.00 | 1.90 |
| *Diaixis hibernica* | 0.00 | 0.00 | 0.00 |  | 0.39 | 0.00 | 0.37 | 0.00 | 1.39 | 0.00 |  | 0.00 | | 0.00 | 0.00 | 0.00 | 0.00 | 0.00 |
| Egg: crustacean | 0.00 | 0.00 | 0.00 |  | 0.77 | 0.85 | 0.03 | 0.01 | 2.78 | 2.94 |  | 0.81 | | 0.85 | 0.02 | 0.00 | 2.00 | 1.27 |
| Egg: *E. encrasicolus* | 0.00 | 0.00 | 0.00 |  | 0.00 | 0.00 | 0.00 | 0.00 | 0.00 | 0.00 |  | 0.00 | | 0.00 | 0.00 | 0.00 | 0.00 | 0.00 |
| Egg: unidentified fish | 0.00 | 0.00 | 0.00 |  | 0.00 | 0.00 | 0.00 | 0.00 | 0.00 | 0.00 |  | 0.81 | | 0.00 | 4.46 | 0.00 | 2.00 | 0.00 |
| *Euterpina acutifrons* | 8.62 | 0.17 | 10.32 |  | 0.39 | 0.11 | 0.04 | 0.00 | 1.39 | 0.59 |  | 0.00 | | 0.00 | 0.00 | 0.00 | 0.00 | 0.00 |
| Fish larvae (unidentified) | 0.00 | 0.00 | 0.00 |  | 0.00 | 0.21 | 0.00 | 0.28 | 0.00 | 1.18 |  | 1.21 | | 0.17 | 6.69 | 0.06 | 2.00 | 0.63 |
| Furcilia larvae | 0.00 | 0.00 | 0.00 |  | 0.39 | 1.17 | 1.33 | 0.82 | 1.39 | 2.35 |  | 0.00 | | 3.56 | 0.00 | 0.69 | 0.00 | 2.53 |
| Gastropod veliger Prosobranchia | 2.59 | 0.05 | 1.59 |  | 0.00 | 0.32 | 0.00 | 0.01 | 0.00 | 0.59 |  | 25.40 | | 1.86 | 2.06 | 0.01 | 4.00 | 2.53 |
| Harpacticoid copepodite | 1.29 | 0.01 | 2.38 |  | 0.00 | 0.32 | 0.00 | 0.00 | 0.00 | 1.76 |  | 2.02 | | 0.17 | 0.07 | 0.00 | 4.00 | 0.63 |
| Harpacticoid naupli | 0.00 | 0.00 | 0.00 |  | 0.00 | 0.00 | 0.00 | 0.00 | 0.00 | 0.00 |  | 0.00 | | 0.00 | 0.00 | 0.00 | 0.00 | 0.00 |
| Hydrozoa (unidentified) | 0.00 | 0.00 | 0.00 |  | 0.00 | 0.00 | 0.00 | 0.00 | 0.00 | 0.00 |  | 0.00 | | 0.34 | 0.00 | 0.50 | 0.00 | 0.63 |
| Insect larva | 0.00 | 0.00 | 0.00 |  | 0.00 | 0.00 | 0.00 | 0.00 | 0.00 | 0.00 |  | 0.00 | | 0.00 | 0.00 | 0.00 | 0.00 | 0.00 |
| *Meganyctiphanes norvegica* | 0.00 | 0.00 | 0.00 |  | 0.00 | 0.21 | 0.00 | 1.56 | 0.00 | 0.59 |  | 0.00 | | 6.95 | 0.00 | 14.04 | 0.00 | 4.43 |
| Metanauplius (Euphausiid) | 0.00 | 0.00 | 0.00 |  | 0.00 | 0.00 | 0.00 | 0.00 | 0.00 | 0.00 |  | 0.00 | | 0.00 | 0.00 | 0.00 | 0.00 | 0.00 |
| *Microsetella rosea* | 0.00 | 0.00 | 0.00 |  | 33.20 | 1.28 | 13.75 | 0.11 | 6.94 | 4.12 |  | 1.61 | | 0.68 | 0.57 | 0.02 | 4.00 | 1.27 |
| *Microsetella* spp. | 0.43 | 0.04 | 0.79 |  | 7.34 | 0.32 | 3.04 | 0.03 | 5.56 | 1.18 |  | 0.81 | | 0.00 | 0.29 | 0.00 | 4.00 | 0.00 |
| *Monstrilla* spp. | 0.00 | 0.00 | 0.00 |  | 0.00 | 0.00 | 0.00 | 0.00 | 0.00 | 0.00 |  | 0.00 | | 0.00 | 0.00 | 0.00 | 0.00 | 0.00 |
| *Nyctiphanes couchi* | 0.43 | 3.25 | 0.79 |  | 0.00 | 1.06 | 0.00 | 7.78 | 0.00 | 0.59 |  | 0.00 | | 10.00 | 0.00 | 20.20 | 0.00 | 6.96 |
| *Oithona* spp. | 0.00 | 0.00 | 0.00 |  | 0.00 | 0.11 | 0.00 | 0.00 | 0.00 | 0.59 |  | 0.00 | | 0.00 | 0.00 | 0.00 | 0.00 | 0.00 |
| *Oncaea* spp. | 6.03 | 0.06 | 8.73 |  | 4.25 | 11.16 | 0.21 | 0.11 | 6.94 | 3.53 |  | 1.61 | | 5.25 | 0.07 | 0.01 | 4.00 | 8.23 |
| *Paraeuchaeta* sp | 0.00 | 0.00 | 0.00 |  | 0.00 | 0.11 | 0.00 | 0.07 | 0.00 | 0.59 |  | 0.00 | | 0.34 | 0.00 | 0.06 | 0.00 | 1.27 |
| *Penilia avirostris* | 0.00 | 0.00 | 0.00 |  | 0.00 | 0.00 | 0.00 | 0.00 | 0.00 | 0.00 |  | 0.00 | | 0.00 | 0.00 | 0.00 | 0.00 | 0.00 |
| *Pleuromamma abdominalis* | 0.00 | 0.00 | 0.00 |  | 1.54 | 1.38 | 1.49 | 0.27 | 2.78 | 2.94 |  | 1.61 | | 4.58 | 1.33 | 0.25 | 2.00 | 5.06 |
| *Podon* sp. | 0.86 | 0.05 | 1.59 |  | 0.39 | 9.78 | 0.10 | 0.53 | 1.39 | 2.94 |  | 0.40 | | 0.17 | 0.09 | 0.00 | 2.00 | 0.63 |
| Siphonostomatoida ord. | 0.00 | 0.00 | 0.00 |  | 0.00 | 0.00 | 0.00 | 0.00 | 0.00 | 0.00 |  | 0.00 | | 0.51 | 0.00 | 0.00 | 0.00 | 1.27 |
| *Temora* spp. | 2.59 | 0.41 | 3.97 |  | 0.00 | 0.11 | 0.00 | 0.02 | 0.00 | 0.59 |  | 0.81 | | 0.17 | 0.51 | 0.01 | 2.00 | 0.63 |
| *Temora stylifera* | 0.00 | 0.00 | 0.00 |  | 0.00 | 0.00 | 0.00 | 0.00 | 0.00 | 0.00 |  | 0.00 | | 0.00 | 0.00 | 0.00 | 0.00 | 0.00 |
| Unidentified amphipod | 1.29 | 15.63 | 2.38 |  | 0.00 | 0.11 | 0.00 | 1.25 | 0.00 | 0.59 |  | 0.00 | | 0.17 | 0.00 | 0.55 | 0.00 | 0.63 |
| Unidentified calanoid | 11.64 | 1.66 | 13.49 |  | 19.31 | 12.22 | 13.08 | 1.70 | 11.11 | 9.41 |  | 6.45 | | 7.97 | 3.75 | 0.31 | 6.00 | 9.49 |
| Unidentified cyclopoid | 0.00 | 0.00 | 0.00 |  | 0.00 | 0.00 | 0.00 | 0.00 | 0.00 | 0.00 |  | 0.00 | | 0.00 | 0.00 | 0.00 | 0.00 | 0.00 |
| Unidentified decapod | 0.00 | 0.00 | 0.00 |  | 0.77 | 4.89 | 20.45 | 26.53 | 2.78 | 2.35 |  | 1.21 | | 3.05 | 27.45 | 4.57 | 4.00 | 2.53 |
| Unidentified euphausiid | 0.43 | 3.25 | 0.79 |  | 0.00 | 3.19 | 0.00 | 23.33 | 0.00 | 1.76 |  | 0.00 | | 24.07 | 0.00 | 48.61 | 0.00 | 10.13 |
| Unidentified isopod | 0.00 | 0.00 | 0.00 |  | 0.00 | 0.11 | 0.00 | 1.25 | 0.00 | 0.59 |  | 0.00 | | 0.00 | 0.00 | 0.00 | 0.00 | 0.00 |
| Unidentified malacostraca | 7.76 | 58.42 | 11.11 |  | 0.00 | 0.00 | 0.00 | 0.00 | 0.00 | 0.00 |  | 0.00 | | 0.00 | 0.00 | 0.00 | 0.00 | 0.00 |
| Unidentified mysid | 0.43 | 3.25 | 0.79 |  | 0.00 | 0.00 | 0.00 | 0.00 | 0.00 | 0.00 |  | 0.40 | | 0.00 | 12.34 | 0.00 | 2.00 | 0.00 |

**b) sardine**

|  | GSA07 | | |  | GSA06-North | | | | | |  | GSA06-South | | | | | |
| --- | --- | --- | --- | --- | --- | --- | --- | --- | --- | --- | --- | --- | --- | --- | --- | --- | --- |
|  | %ABD | %BIO | %FO |  | %ABD | | %BIO | | %FO | |  | %ABD | | %BIO | | %FO | |
| Prey group (species)⇓ stage⇒ | juv | juv | juv |  | juv | ad | juv | ad | juv | ad |  | juv | ad | juv | ad | juv | ad |
| *Acartia* spp. | 6.49 | 2.07 | 6.00 |  | 20.54 | 15.12 | 0.31 | 0.94 | 12.16 | 9.17 |  | 1.71 | 2.92 | 0.09 | 0.01 | 3.73 | 5.15 |
| Bivalve veliger | 0.00 | 0.00 | 0.00 |  | 1.16 | 0.00 | 0.01 | 0.00 | 4.05 | 0.00 |  | 1.50 | 0.25 | 0.05 | 0.00 | 3.73 | 1.55 |
| Calanoid copepodite | 18.92 | 4.12 | 17.00 |  | 8.53 | 10.39 | 0.09 | 0.44 | 14.86 | 5.83 |  | 3.43 | 5.43 | 0.13 | 0.02 | 6.83 | 7.73 |
| Calanoid naupli | 4.32 | 0.29 | 7.00 |  | 0.78 | 1.10 | 0.00 | 0.01 | 1.35 | 3.33 |  | 50.00 | 3.76 | 0.57 | 0.00 | 7.45 | 6.19 |
| *Calanus gracilis* | 0.00 | 0.00 | 0.00 |  | 0.00 | 0.00 | 0.00 | 0.00 | 0.00 | 0.00 |  | 0.00 | 0.00 | 0.00 | 0.00 | 0.00 | 0.00 |
| *Calanus helgolandicus* | 0.00 | 0.00 | 0.00 |  | 0.00 | 2.05 | 0.00 | 2.05 | 0.00 | 4.17 |  | 0.21 | 1.92 | 0.19 | 0.14 | 1.24 | 3.61 |
| *Calocalanus tenuis* | 0.00 | 0.00 | 0.00 |  | 0.00 | 0.00 | 0.00 | 0.00 | 0.00 | 0.00 |  | 0.00 | 0.00 | 0.00 | 0.00 | 0.00 | 0.00 |
| Calyptopis larvae | 0.00 | 0.00 | 0.00 |  | 0.00 | 0.16 | 0.00 | 2.78 | 0.00 | 0.83 |  | 0.00 | 14.29 | 0.00 | 18.07 | 0.00 | 5.67 |
| *Candacia armata* | 0.00 | 0.00 | 0.00 |  | 1.94 | 4.41 | 2.58 | 24.52 | 4.05 | 5.00 |  | 1.07 | 1.75 | 5.24 | 0.70 | 4.35 | 4.12 |
| *Centropages chierchiae* | 0.00 | 0.00 | 0.00 |  | 0.00 | 0.00 | 0.00 | 0.00 | 0.00 | 0.00 |  | 0.21 | 0.00 | 0.08 | 0.00 | 1.24 | 0.00 |
| *Centropages* spp. | 0.00 | 0.00 | 0.00 |  | 0.00 | 0.31 | 0.00 | 0.13 | 0.00 | 0.83 |  | 0.32 | 0.25 | 0.12 | 0.01 | 1.86 | 1.03 |
| *Centropages typicus* | 1.08 | 2.37 | 1.00 |  | 0.39 | 0.00 | 0.04 | 0.00 | 1.35 | 0.00 |  | 0.00 | 0.08 | 0.00 | 0.00 | 0.00 | 0.52 |
| Cirripedia | 0.00 | 0.00 | 0.00 |  | 0.00 | 0.00 | 0.00 | 0.00 | 0.00 | 0.00 |  | 1.93 | 0.08 | 0.15 | 0.00 | 4.97 | 0.52 |
| Cirripedia naupli | 0.00 | 0.00 | 0.00 |  | 0.00 | 0.00 | 0.00 | 0.00 | 0.00 | 0.00 |  | 0.32 | 0.00 | 0.05 | 0.00 | 1.24 | 0.00 |
| Clauso-/Para-calanidae fam. | 1.62 | 8.29 | 3.00 |  | 8.53 | 15.59 | 2.04 | 15.59 | 12.16 | 7.50 |  | 0.75 | 1.34 | 0.66 | 0.10 | 3.11 | 3.61 |
| *Clausocalanus* sp. | 0.00 | 0.00 | 0.00 |  | 0.00 | 0.94 | 0.00 | 2.19 | 0.00 | 4.17 |  | 0.21 | 0.33 | 0.44 | 0.06 | 1.24 | 1.55 |
| *Clytemnestra* spp. | 1.08 | 1.83 | 2.00 |  | 0.78 | 0.16 | 0.06 | 0.05 | 2.70 | 0.83 |  | 0.00 | 0.08 | 0.00 | 0.00 | 0.00 | 0.52 |
| *Corycaeus anglicus* | 1.62 | 1.88 | 3.00 |  | 0.00 | 6.14 | 0.00 | 1.39 | 0.00 | 5.83 |  | 0.54 | 1.00 | 0.11 | 0.02 | 1.24 | 3.09 |
| Cyclopoid copepodite | 0.00 | 0.00 | 0.00 |  | 0.00 | 2.20 | 0.00 | 0.09 | 0.00 | 3.33 |  | 0.54 | 0.00 | 0.02 | 0.00 | 1.86 | 0.00 |
| Cyclopoid.naupli | 0.00 | 0.00 | 0.00 |  | 0.00 | 0.00 | 0.00 | 0.00 | 0.00 | 0.00 |  | 1.61 | 0.17 | 0.02 | 0.00 | 3.11 | 1.03 |
| Decapod late larvae | 0.00 | 0.00 | 0.00 |  | 0.39 | 0.00 | 1.64 | 0.00 | 1.35 | 0.00 |  | 3.21 | 3.68 | 49.95 | 4.65 | 3.11 | 5.67 |
| Decapod megalopa larvae | 0.00 | 0.00 | 0.00 |  | 3.88 | 0.79 | 9.59 | 8.13 | 1.35 | 3.33 |  | 0.64 | 0.00 | 5.84 | 0.00 | 1.86 | 0.00 |
| Decapod zoea larvae | 0.00 | 0.00 | 0.00 |  | 1.94 | 0.00 | 4.80 | 0.00 | 2.70 | 0.00 |  | 0.11 | 0.00 | 0.97 | 0.00 | 0.62 | 0.00 |
| *Diaixis hibernica* | 0.00 | 0.00 | 0.00 |  | 0.00 | 0.00 | 0.00 | 0.00 | 0.00 | 0.00 |  | 0.00 | 0.00 | 0.00 | 0.00 | 0.00 | 0.00 |
| Egg: crustacean | 0.54 | 0.10 | 1.00 |  | 1.55 | 1.10 | 0.01 | 0.04 | 5.41 | 3.33 |  | 0.00 | 0.00 | 0.00 | 0.00 | 0.00 | 0.00 |
| Egg: *E. encrasicolus* | 0.00 | 0.00 | 0.00 |  | 0.00 | 0.79 | 0.00 | 7.59 | 0.00 | 3.33 |  | 0.21 | 0.00 | 1.82 | 0.00 | 1.24 | 0.00 |
| Egg: unidentified fish | 1.08 | 37.07 | 2.00 |  | 0.00 | 0.94 | 0.00 | 6.33 | 0.00 | 4.17 |  | 1.71 | 0.08 | 10.10 | 0.04 | 5.59 | 0.52 |
| *Euterpina acutifrons* | 17.84 | 8.72 | 15.00 |  | 0.00 | 0.79 | 0.00 | 0.08 | 0.00 | 3.33 |  | 1.39 | 0.08 | 0.12 | 0.00 | 4.35 | 0.52 |
| Fish larvae (unidentified) | 0.00 | 0.00 | 0.00 |  | 0.00 | 0.00 | 0.00 | 0.00 | 0.00 | 0.00 |  | 0.00 | 0.00 | 0.00 | 0.00 | 0.00 | 0.00 |
| Furcilia larvae | 0.00 | 0.00 | 0.00 |  | 3.88 | 0.16 | 3.31 | 0.56 | 4.05 | 0.83 |  | 0.00 | 1.84 | 0.00 | 0.47 | 0.00 | 3.61 |
| Gastropod veliger Prosobranchia | 1.08 | 0.54 | 2.00 |  | 0.00 | 0.00 | 0.00 | 0.00 | 0.00 | 0.00 |  | 0.54 | 0.42 | 0.05 | 0.00 | 1.24 | 2.06 |
| Harpacticoid copepodite | 17.30 | 3.77 | 15.00 |  | 0.00 | 0.00 | 0.00 | 0.00 | 0.00 | 0.00 |  | 0.43 | 0.58 | 0.02 | 0.00 | 1.24 | 1.55 |
| Harpacticoid naupli | 0.00 | 0.00 | 0.00 |  | 0.39 | 0.00 | 0.00 | 0.00 | 1.35 | 0.00 |  | 0.00 | 0.00 | 0.00 | 0.00 | 0.00 | 0.00 |
| Hydrozoa (unidentified) | 0.00 | 0.00 | 0.00 |  | 0.00 | 0.00 | 0.00 | 0.00 | 0.00 | 0.00 |  | 0.21 | 0.00 | 5.14 | 0.00 | 0.62 | 0.00 |
| Insect larva | 0.00 | 0.00 | 0.00 |  | 0.00 | 0.00 | 0.00 | 0.00 | 0.00 | 0.00 |  | 0.11 | 0.00 | 3.49 | 0.00 | 0.62 | 0.00 |
| *Meganyctiphanes norvegica* | 0.00 | 0.00 | 0.00 |  | 0.00 | 0.00 | 0.00 | 0.00 | 0.00 | 0.00 |  | 0.00 | 20.63 | 0.00 | 54.78 | 0.00 | 5.67 |
| Metanauplius (Euphausiid) | 0.00 | 0.00 | 0.00 |  | 0.00 | 0.00 | 0.00 | 0.00 | 0.00 | 0.00 |  | 0.00 | 0.17 | 0.00 | 0.00 | 0.00 | 0.52 |
| *Microsetella rosea* | 0.00 | 0.00 | 0.00 |  | 0.00 | 0.47 | 0.00 | 0.20 | 0.00 | 1.67 |  | 1.39 | 1.67 | 0.53 | 0.05 | 2.48 | 4.64 |
| *Microsetella* spp. | 0.54 | 1.19 | 1.00 |  | 0.00 | 0.16 | 0.00 | 0.07 | 0.00 | 0.83 |  | 5.89 | 1.34 | 2.23 | 0.04 | 6.83 | 5.15 |
| *Monstrilla* spp. | 0.54 | 2.76 | 1.00 |  | 0.00 | 0.00 | 0.00 | 0.00 | 0.00 | 0.00 |  | 0.00 | 0.00 | 0.00 | 0.00 | 0.00 | 0.00 |
| *Nyctiphanes couchi* | 0.00 | 0.00 | 0.00 |  | 0.00 | 0.00 | 0.00 | 0.00 | 0.00 | 0.00 |  | 0.00 | 2.17 | 0.00 | 5.77 | 0.00 | 1.55 |
| *Oithona* spp. | 0.00 | 0.00 | 0.00 |  | 0.00 | 0.00 | 0.00 | 0.00 | 0.00 | 0.00 |  | 0.00 | 0.00 | 0.00 | 0.00 | 0.00 | 0.00 |
| *Oncaea* spp. | 20.54 | 5.35 | 16.00 |  | 0.00 | 4.88 | 0.00 | 0.25 | 0.00 | 5.00 |  | 12.74 | 18.21 | 0.57 | 0.07 | 8.07 | 8.25 |
| *Paraeuchaeta* sp | 0.00 | 0.00 | 0.00 |  | 0.00 | 0.00 | 0.00 | 0.00 | 0.00 | 0.00 |  | 0.00 | 0.17 | 0.00 | 0.04 | 0.00 | 1.03 |
| *Penilia avirostris* | 0.00 | 0.00 | 0.00 |  | 0.00 | 0.00 | 0.00 | 0.00 | 0.00 | 0.00 |  | 0.00 | 0.00 | 0.00 | 0.00 | 0.00 | 0.00 |
| *Pleuromamma abdominalis* | 0.00 | 0.00 | 0.00 |  | 0.00 | 0.00 | 0.00 | 0.00 | 0.00 | 0.00 |  | 0.00 | 0.00 | 0.00 | 0.00 | 0.00 | 0.00 |
| *Podon* sp. | 0.00 | 0.00 | 0.00 |  | 0.39 | 9.29 | 0.03 | 2.53 | 1.35 | 4.17 |  | 1.82 | 0.00 | 0.44 | 0.00 | 3.73 | 0.00 |
| Siphonostomatoida ord. | 0.00 | 0.00 | 0.00 |  | 0.00 | 0.00 | 0.00 | 0.00 | 0.00 | 0.00 |  | 0.00 | 0.00 | 0.00 | 0.00 | 0.00 | 0.00 |
| *Temora* spp. | 0.54 | 2.14 | 1.00 |  | 0.00 | 2.36 | 0.00 | 1.83 | 0.00 | 4.17 |  | 0.32 | 0.33 | 0.22 | 0.02 | 1.24 | 1.55 |
| *Temora stylifera* | 0.00 | 0.00 | 0.00 |  | 0.00 | 0.00 | 0.00 | 0.00 | 0.00 | 0.00 |  | 0.00 | 0.00 | 0.00 | 0.00 | 0.00 | 0.00 |
| Unidentified amphipod | 0.00 | 0.00 | 0.00 |  | 0.00 | 0.00 | 0.00 | 0.00 | 0.00 | 0.00 |  | 0.00 | 0.00 | 0.00 | 0.00 | 0.00 | 0.00 |
| Unidentified calanoid | 4.86 | 17.51 | 7.00 |  | 35.27 | 19.21 | 5.95 | 13.52 | 18.92 | 12.50 |  | 4.60 | 9.11 | 2.85 | 0.46 | 8.70 | 8.25 |
| Unidentified cyclopoid | 0.00 | 0.00 | 0.00 |  | 0.00 | 0.16 | 0.00 | 0.01 | 0.00 | 0.83 |  | 0.00 | 0.00 | 0.00 | 0.00 | 0.00 | 0.00 |
| Unidentified decapod | 0.00 | 0.00 | 0.00 |  | 8.14 | 0.31 | 53.66 | 8.66 | 6.76 | 1.67 |  | 0.32 | 1.50 | 7.77 | 2.96 | 1.24 | 0.52 |
| Unidentified euphausiid | 0.00 | 0.00 | 0.00 |  | 1.16 | 0.00 | 10.34 | 0.00 | 2.70 | 0.00 |  | 0.00 | 4.34 | 0.00 | 11.53 | 0.00 | 3.09 |
| Unidentified isopod | 0.00 | 0.00 | 0.00 |  | 0.39 | 0.00 | 5.53 | 0.00 | 1.35 | 0.00 |  | 0.00 | 0.00 | 0.00 | 0.00 | 0.00 | 0.00 |
| Unidentified malacostraca | 0.00 | 0.00 | 0.00 |  | 0.00 | 0.00 | 0.00 | 0.00 | 0.00 | 0.00 |  | 0.00 | 0.00 | 0.00 | 0.00 | 0.00 | 0.00 |
| Unidentified mysid | 0.00 | 0.00 | 0.00 |  | 0.00 | 0.00 | 0.00 | 0.00 | 0.00 | 0.00 |  | 0.00 | 0.00 | 0.00 | 0.00 | 0.00 | 0.00 |

**Table S05.** Diet composition observed under the microscope for **(a)** anchovy and **(b)** sardine, presented as numerical frequency (%ABD), estimated biomass (%BIO) and frequency of occurrence (%FO), by stage (*juv*: juveniles; *ad*: adults) and areas (GSA07, GSA06-North, GSA06-South). Number of samples (N) for each category is presented in Table 6.

**a) anchovy**

|  | GSA07 |  | GSA06-North | |  | GSA06-South | |
| --- | --- | --- | --- | --- | --- | --- | --- |
| Prey group (species)⇓ stage⇒ | juv |  | juv | ad |  | juv | ad |
| *Abylopsis tetragona* | 0.00 |  | 0.00 | 0.95 |  | 0.00 | 2.50 |
| *Acartia clausi* | 8.70 |  | 6.01 | 1.58 |  | 3.00 | 1.88 |
| *Acartia discaudata* | 7.61 |  | 0.55 | 0.00 |  | 0.00 | 0.00 |
| Actiniaria ord | 0.00 |  | 0.00 | 1.58 |  | 0.00 | 0.00 |
| *Aglaura hemistoma* | 2.17 |  | 1.09 | 2.52 |  | 2.00 | 1.25 |
| *Ampelisca brevicornis* | 3.26 |  | 0.55 | 0.00 |  | 0.00 | 0.00 |
| *Ampelisca typica* | 1.09 |  | 0.00 | 0.00 |  | 0.00 | 0.00 |
| *Amphiura filiformis* | 0.00 |  | 0.00 | 0.32 |  | 0.00 | 0.00 |
| *Anapagurus breviaculeatus* | 0.00 |  | 0.00 | 0.00 |  | 0.00 | 0.00 |
| *Anapagurus chiroacanthus* | 0.00 |  | 0.00 | 0.00 |  | 0.00 | 0.00 |
| *Aporrhais pespelecani* | 0.00 |  | 0.00 | 0.63 |  | 1.00 | 0.63 |
| *Archiconchoecia striata* | 0.00 |  | 2.19 | 0.63 |  | 0.00 | 0.00 |
| *Bougainvillia muscus* | 0.00 |  | 0.55 | 0.00 |  | 0.00 | 1.25 |
| *Bradyidius armatus* | 0.00 |  | 0.00 | 0.00 |  | 0.00 | 1.88 |
| *Caecum trachea* | 0.00 |  | 0.00 | 0.00 |  | 1.00 | 0.00 |
| Calanoida ord. | 0.00 |  | 0.00 | 0.00 |  | 0.00 | 0.63 |
| *Calanus euxinus* | 2.17 |  | 1.64 | 1.89 |  | 3.00 | 0.00 |
| *Calanus helgolandicus* | 1.09 |  | 0.55 | 2.21 |  | 4.00 | 2.50 |
| *Calocalanus contractus* | 2.17 |  | 2.73 | 3.15 |  | 0.00 | 0.00 |
| *Calocalanus styliremis* | 0.00 |  | 0.55 | 0.00 |  | 0.00 | 0.00 |
| *Candacia armata* | 0.00 |  | 0.00 | 1.26 |  | 0.00 | 0.00 |
| *Cavolinia inflexa* | 0.00 |  | 0.00 | 0.00 |  | 0.00 | 0.63 |
| Cavoliniidae fam. | 0.00 |  | 0.00 | 0.32 |  | 0.00 | 0.00 |
| *Centropages typicus* | 0.00 |  | 0.00 | 0.32 |  | 0.00 | 0.00 |
| *Chaetopterus variopedatus* | 0.00 |  | 0.00 | 0.00 |  | 0.00 | 0.00 |
| *Chlorotocus crassicornis* | 0.00 |  | 0.00 | 0.00 |  | 0.00 | 0.63 |
| *Clausocalanus arcuicornis* | 0.00 |  | 3.28 | 4.42 |  | 2.00 | 0.00 |
| *Clausocalanus jobei* | 0.00 |  | 0.00 | 0.95 |  | 3.00 | 0.00 |
| *Clausocalanus lividus* | 0.00 |  | 7.10 | 4.10 |  | 0.00 | 2.50 |
| *Clausocalanus parapergens* | 0.00 |  | 0.00 | 0.00 |  | 0.00 | 0.00 |
| *Clausocalanus paululus* | 1.09 |  | 2.19 | 2.21 |  | 1.00 | 1.88 |
| *Clausocalanus pergens* | 4.35 |  | 7.10 | 5.68 |  | 4.00 | 3.75 |
| *Clymenura clypeata* | 0.00 |  | 0.00 | 0.00 |  | 0.00 | 0.00 |
| *Clytia hemisphaerica* | 0.00 |  | 1.09 | 0.95 |  | 4.00 | 2.50 |
| Coccolithus sp. | 0.00 |  | 0.55 | 0.63 |  | 1.00 | 3.13 |
| *Copilia quadrata* | 4.35 |  | 7.65 | 4.73 |  | 5.00 | 6.25 |
| *Corbula gibba* | 0.00 |  | 0.00 | 0.32 |  | 0.00 | 0.00 |
| Corymorpha sp. | 0.00 |  | 0.00 | 0.63 |  | 0.00 | 1.88 |
| *Crystallogobius linearis* | 0.00 |  | 0.00 | 0.32 |  | 0.00 | 0.00 |
| Decapoda ord. | 0.00 |  | 0.00 | 0.00 |  | 0.00 | 0.63 |
| *Deltentosteus quadrimaculatus* | 2.17 |  | 0.00 | 0.00 |  | 0.00 | 0.00 |
| *Derilambrus angulifrons* | 0.00 |  | 0.00 | 0.00 |  | 0.00 | 0.00 |
| *Diaixis hibernica* | 4.35 |  | 1.09 | 2.52 |  | 2.00 | 5.00 |
| *Ditrichocorycaeus anglicus* | 0.00 |  | 0.00 | 0.32 |  | 0.00 | 1.25 |
| *Ebalia cranchii* | 0.00 |  | 0.00 | 0.00 |  | 0.00 | 0.00 |
| *Echiodon drummondii* | 0.00 |  | 0.00 | 0.00 |  | 0.00 | 0.63 |
| *Embletonia pulchra* | 0.00 |  | 0.00 | 0.00 |  | 0.00 | 0.00 |
| *Euchaeta* sp. | 0.00 |  | 0.55 | 1.26 |  | 0.00 | 0.00 |
| *Euphausia krohni* | 0.00 |  | 0.00 | 0.00 |  | 0.00 | 1.25 |
| *Euphysa aurata* | 0.00 |  | 0.00 | 0.63 |  | 0.00 | 0.00 |
| *Eurynome spinosa* | 0.00 |  | 0.00 | 0.00 |  | 0.00 | 0.63 |
| *Eusergestes arcticus* | 0.00 |  | 0.00 | 0.00 |  | 0.00 | 0.63 |
| *Euspira nitida* | 0.00 |  | 0.00 | 0.32 |  | 1.00 | 0.63 |
| *Euterpina acutifrons* | 0.00 |  | 0.00 | 0.00 |  | 1.00 | 0.00 |
| *Evadne nordmanni* | 0.00 |  | 0.00 | 0.00 |  | 1.00 | 0.00 |
| Gastropoda cl. | 0.00 |  | 0.55 | 0.32 |  | 0.00 | 0.00 |
| *Goneplax rhomboides* | 3.26 |  | 0.55 | 0.63 |  | 0.00 | 0.63 |
| *Gracilechinus acutus* | 0.00 |  | 0.00 | 0.00 |  | 0.00 | 0.63 |
| *Gymnammodytes cicerelus* | 0.00 |  | 0.00 | 0.00 |  | 0.00 | 0.00 |
| *Helgicirrha cari* | 0.00 |  | 0.00 | 0.00 |  | 0.00 | 0.63 |
| *Illex coindetii* | 0.00 |  | 0.00 | 0.00 |  | 0.00 | 0.00 |
| *Inachus dorsettensis* | 0.00 |  | 0.00 | 0.00 |  | 0.00 | 0.00 |
| *Inachus communissimus* | 1.09 |  | 0.00 | 0.32 |  | 0.00 | 0.00 |
| *Jaxea nocturna* | 1.09 |  | 0.00 | 0.63 |  | 0.00 | 0.00 |
| *Labioleanira yhleni* | 0.00 |  | 0.55 | 0.00 |  | 0.00 | 0.00 |
| *Lesueurigobius friesii* | 3.26 |  | 0.55 | 0.00 |  | 0.00 | 0.00 |
| *Leuckartiara octona* | 0.00 |  | 0.55 | 1.26 |  | 0.00 | 0.00 |
| *Limacina inflata* | 0.00 |  | 0.00 | 0.32 |  | 0.00 | 0.00 |
| *Liocarcinus depurator* | 0.00 |  | 0.00 | 0.00 |  | 0.00 | 0.63 |
| *Liocarcinus maculatus* | 0.00 |  | 1.64 | 0.32 |  | 0.00 | 0.63 |
| *Liocarcinus vernalis* | 1.09 |  | 0.00 | 0.32 |  | 1.00 | 0.63 |
| *Lizzia blondina* | 4.35 |  | 1.09 | 2.52 |  | 2.00 | 3.13 |
| *Longipedia* sp. | 0.00 |  | 1.09 | 0.00 |  | 1.00 | 0.00 |
| *Lucicutia flavicornis* | 0.00 |  | 0.00 | 0.00 |  | 0.00 | 0.00 |
| *Luidia sarsi* | 0.00 |  | 0.00 | 0.00 |  | 0.00 | 0.63 |
| *Magelona* sp. | 2.17 |  | 1.09 | 1.26 |  | 1.00 | 0.00 |
| *Mecynocera clausi* | 0.00 |  | 1.09 | 0.00 |  | 1.00 | 0.00 |
| *Meganyctiphanes norvegica* | 0.00 |  | 0.00 | 0.63 |  | 0.00 | 0.63 |
| *Merluccius merluccius* | 0.00 |  | 0.00 | 0.00 |  | 0.00 | 0.00 |
| *Mesaiokeras hurei* | 0.00 |  | 0.00 | 0.32 |  | 0.00 | 0.00 |
| *Mesocalanus tenuicornis* | 0.00 |  | 1.09 | 1.89 |  | 0.00 | 0.00 |
| *Microsetella norvegica* | 0.00 |  | 2.73 | 3.15 |  | 3.00 | 2.50 |
| *Muggiaea atlantica* | 1.09 |  | 0.00 | 1.89 |  | 5.00 | 3.13 |
| *Mullus barbatus* | 0.00 |  | 0.00 | 0.32 |  | 0.00 | 0.00 |
| *Nannocalanus minor* | 0.00 |  | 0.00 | 0.00 |  | 3.00 | 1.88 |
| *Nanomia bijuga* | 0.00 |  | 6.56 | 5.36 |  | 4.00 | 1.25 |
| *Nematoscelis megalops* | 0.00 |  | 1.64 | 0.63 |  | 1.00 | 1.88 |
| *Obelia dichotoma* | 0.00 |  | 0.00 | 0.63 |  | 2.00 | 2.50 |
| *Oncaea mediterranea* | 0.00 |  | 0.55 | 0.63 |  | 0.00 | 0.00 |
| *Oncaea scottodicarloi* | 0.00 |  | 0.00 | 0.00 |  | 0.00 | 0.00 |
| *Oncaea venusta* | 0.00 |  | 0.00 | 0.32 |  | 1.00 | 0.00 |
| *Ophiocomina nigra* | 0.00 |  | 0.00 | 0.00 |  | 0.00 | 0.00 |
| *Ophiura grubei* | 0.00 |  | 0.00 | 0.32 |  | 1.00 | 0.00 |
| *Ophiura ophiura* | 0.00 |  | 0.00 | 0.00 |  | 0.00 | 0.00 |
| *Pagurus prideaux* | 0.00 |  | 0.00 | 0.00 |  | 0.00 | 0.63 |
| *Pandalina brevirostris* | 0.00 |  | 0.00 | 0.32 |  | 2.00 | 1.25 |
| *Paracalanus parvus* | 8.70 |  | 6.56 | 3.15 |  | 5.00 | 5.00 |
| *Paracalanus spp.* | 8.70 |  | 2.73 | 0.95 |  | 3.00 | 1.88 |
| *Paracentrotus lividus* | 0.00 |  | 2.19 | 1.26 |  | 1.00 | 0.00 |
| *Pasiphaea sivado* | 0.00 |  | 0.00 | 0.00 |  | 0.00 | 0.63 |
| *Pectinaria koreni* | 0.00 |  | 0.00 | 0.00 |  | 0.00 | 0.00 |
| *Penilia avirostris* | 0.00 |  | 0.00 | 0.00 |  | 0.00 | 0.00 |
| *Philocheras bispinosus* | 1.09 |  | 0.00 | 2.21 |  | 3.00 | 3.75 |
| *Phrosina semilunata* | 0.00 |  | 0.00 | 0.00 |  | 0.00 | 1.25 |
| *Phyllodoce rosea* | 0.00 |  | 0.00 | 0.00 |  | 0.00 | 0.00 |
| Phyllodocida ord. | 0.00 |  | 0.55 | 0.32 |  | 0.00 | 0.00 |
| *Pleopis polyphemoides* | 5.43 |  | 1.64 | 0.00 |  | 1.00 | 0.00 |
| *Pleuromamma borealis* | 0.00 |  | 0.00 | 0.95 |  | 0.00 | 1.25 |
| *Pleuromamma gracilis* | 0.00 |  | 1.09 | 1.89 |  | 1.00 | 0.63 |
| *Podocoryna areolata* | 0.00 |  | 0.00 | 0.32 |  | 1.00 | 1.25 |
| *Podon intermedius* | 0.00 |  | 0.00 | 1.58 |  | 1.00 | 1.25 |
| Poecilostomatoida ord. | 0.00 |  | 0.00 | 0.00 |  | 0.00 | 0.00 |
| *Pomatoschistus pictus* | 1.09 |  | 0.00 | 0.00 |  | 0.00 | 0.00 |
| Porifera ph. | 0.00 |  | 0.55 | 0.95 |  | 1.00 | 3.13 |
| *Porroecia spinirostris* | 0.00 |  | 0.55 | 0.32 |  | 0.00 | 0.00 |
| *Proceroecia microprocera* | 0.00 |  | 0.55 | 0.00 |  | 0.00 | 0.00 |
| *Processa edulis* | 0.00 |  | 0.00 | 0.32 |  | 0.00 | 0.00 |
| *Processa modica* | 6.52 |  | 1.09 | 0.32 |  | 3.00 | 1.25 |
| *Processa nouveli* | 1.09 |  | 1.64 | 1.26 |  | 2.00 | 2.50 |
| *Pseudameira* sp. | 0.00 |  | 0.55 | 0.00 |  | 0.00 | 0.00 |
| *Pseudocuma similis* | 1.09 |  | 0.00 | 0.00 |  | 0.00 | 0.00 |
| *Pseudosagitta lyra* | 1.09 |  | 0.00 | 0.32 |  | 0.00 | 0.63 |
| *Pusillina inconspicua* | 0.00 |  | 0.00 | 0.00 |  | 1.00 | 0.00 |
| *Rhincalanus nasutus* | 0.00 |  | 0.00 | 0.32 |  | 0.00 | 1.25 |
| *Rissoides desmaresti* | 0.00 |  | 0.00 | 0.00 |  | 0.00 | 0.00 |
| *Salmo salar* | 0.00 |  | 1.64 | 0.00 |  | 0.00 | 0.00 |
| *Sciaena umbra* | 0.00 |  | 0.00 | 0.00 |  | 1.00 | 0.00 |
| *Scolaricia* sp. | 0.00 |  | 0.00 | 0.00 |  | 0.00 | 0.00 |
| *Scolecithricella dentata* | 0.00 |  | 1.64 | 0.95 |  | 0.00 | 0.00 |
| *Sepia officinalis* | 0.00 |  | 0.00 | 0.00 |  | 0.00 | 0.00 |
| *Solenocera membranacea* | 0.00 |  | 0.55 | 3.15 |  | 2.00 | 1.25 |
| *Sphaeronectes gracilis* | 0.00 |  | 0.00 | 0.32 |  | 0.00 | 0.00 |
| *Spicara maena* | 0.00 |  | 0.55 | 1.26 |  | 3.00 | 1.25 |
| *Spisula subtruncata* | 0.00 |  | 1.09 | 0.95 |  | 0.00 | 0.00 |
| *Sprattus sprattus* | 2.17 |  | 0.00 | 0.00 |  | 0.00 | 0.00 |
| *Stauridiosarsia gemmifera* | 1.09 |  | 3.28 | 0.63 |  | 0.00 | 0.00 |
| Stomiiformes ord. | 0.00 |  | 1.09 | 0.32 |  | 0.00 | 0.00 |
| *Subeucalanus pileatus* | 0.00 |  | 0.00 | 0.32 |  | 1.00 | 0.00 |
| Terebellida ord. | 0.00 |  | 0.00 | 0.00 |  | 0.00 | 0.00 |
| *Triconia dentipes* | 0.00 |  | 0.00 | 0.00 |  | 0.00 | 0.63 |
| *Trisopterus capelanus* | 0.00 |  | 0.00 | 1.26 |  | 1.00 | 0.00 |
| Tubulanidae fam. | 0.00 |  | 2.19 | 2.21 |  | 2.00 | 1.88 |
| Unidentified decapod | 0.00 |  | 0.00 | 0.32 |  | 0.00 | 0.00 |
| *Vibilia armata* | 0.00 |  | 0.00 | 0.32 |  | 0.00 | 0.00 |

**b) sardine**

|  | GSA07 |  | GSA06-North | |  | GSA06-South | |
| --- | --- | --- | --- | --- | --- | --- | --- |
| Prey group (species)⇓ stage⇒ | juv |  | juv | ad |  | juv | ad |
| *Abylopsis tetragona* | 0.00 |  | 0.00 | 0.74 |  | 0.00 | 0.00 |
| *Acartia clausi* | 4.92 |  | 7.32 | 2.95 |  | 3.30 | 2.83 |
| *Acartia discaudata* | 4.92 |  | 0.00 | 0.00 |  | 0.00 | 0.00 |
| Actiniaria ord | 0.55 |  | 0.00 | 1.48 |  | 0.00 | 0.00 |
| *Aglaura hemistoma* | 3.83 |  | 1.22 | 2.58 |  | 0.47 | 0.94 |
| *Ampelisca brevicornis* | 0.00 |  | 0.00 | 0.00 |  | 0.00 | 0.00 |
| *Ampelisca typica* | 0.00 |  | 0.00 | 0.00 |  | 0.00 | 0.00 |
| *Amphiura filiformis* | 0.00 |  | 0.00 | 0.00 |  | 0.00 | 0.00 |
| *Anapagurus breviaculeatus* | 0.00 |  | 0.00 | 0.00 |  | 0.24 | 0.94 |
| *Anapagurus chiroacanthus* | 0.00 |  | 0.00 | 0.00 |  | 0.00 | 0.47 |
| *Aporrhais pespelecani* | 0.00 |  | 0.00 | 0.74 |  | 0.94 | 0.47 |
| *Archiconchoecia striata* | 0.00 |  | 0.00 | 0.37 |  | 0.00 | 0.00 |
| *Bougainvillia muscus* | 0.00 |  | 0.00 | 0.37 |  | 0.00 | 4.72 |
| *Bradyidius armatus* | 0.00 |  | 0.00 | 0.37 |  | 0.00 | 4.25 |
| *Caecum trachea* | 0.00 |  | 0.61 | 0.00 |  | 0.94 | 0.47 |
| Calanoida ord. | 0.55 |  | 0.00 | 0.00 |  | 1.89 | 0.47 |
| *Calanus euxinus* | 1.09 |  | 1.22 | 4.43 |  | 2.36 | 0.47 |
| *Calanus helgolandicus* | 0.55 |  | 1.83 | 3.69 |  | 3.30 | 2.36 |
| *Calocalanus contractus* | 2.73 |  | 2.44 | 2.58 |  | 2.36 | 0.47 |
| *Calocalanus styliremis* | 2.73 |  | 1.22 | 0.74 |  | 2.59 | 0.47 |
| *Candacia armata* | 0.00 |  | 0.00 | 1.11 |  | 0.71 | 0.47 |
| *Cavolinia inflexa* | 0.00 |  | 0.00 | 0.00 |  | 0.00 | 0.00 |
| Cavoliniidae fam. | 0.00 |  | 0.00 | 0.00 |  | 0.24 | 0.00 |
| *Centropages typicus* | 0.55 |  | 1.22 | 0.37 |  | 0.00 | 0.00 |
| *Chaetopterus variopedatus* | 0.00 |  | 0.00 | 0.37 |  | 0.00 | 0.00 |
| *Chlorotocus crassicornis* | 0.00 |  | 0.00 | 0.00 |  | 0.00 | 0.00 |
| *Clausocalanus arcuicornis* | 0.55 |  | 3.66 | 3.69 |  | 1.18 | 0.47 |
| *Clausocalanus jobei* | 0.00 |  | 0.00 | 2.21 |  | 1.65 | 0.47 |
| *Clausocalanus lividus* | 0.00 |  | 7.32 | 3.69 |  | 0.71 | 0.94 |
| *Clausocalanus parapergens* | 0.00 |  | 0.00 | 0.00 |  | 0.00 | 0.94 |
| *Clausocalanus paululus* | 0.55 |  | 1.22 | 0.37 |  | 0.24 | 1.89 |
| *Clausocalanus pergens* | 3.83 |  | 7.32 | 4.80 |  | 3.54 | 5.19 |
| *Clymenura clypeata* | 0.00 |  | 0.00 | 0.37 |  | 0.00 | 0.00 |
| *Clytia hemisphaerica* | 0.55 |  | 1.22 | 0.74 |  | 3.54 | 1.42 |
| Coccolithus sp. | 0.00 |  | 0.00 | 0.37 |  | 1.18 | 0.94 |
| *Copilia quadrata* | 4.37 |  | 6.71 | 3.32 |  | 2.36 | 3.77 |
| *Corbula gibba* | 0.00 |  | 0.00 | 0.37 |  | 0.24 | 0.47 |
| Corymorpha sp. | 0.00 |  | 0.00 | 0.37 |  | 0.71 | 1.89 |
| *Crystallogobius linearis* | 0.00 |  | 0.00 | 0.00 |  | 0.00 | 0.00 |
| Decapoda ord. | 0.00 |  | 0.00 | 0.00 |  | 0.00 | 0.00 |
| *Deltentosteus quadrimaculatus* | 0.00 |  | 0.00 | 0.00 |  | 0.00 | 0.00 |
| *Derilambrus angulifrons* | 0.00 |  | 1.83 | 0.00 |  | 0.00 | 0.00 |
| *Diaixis hibernica* | 0.00 |  | 0.00 | 2.95 |  | 1.89 | 5.66 |
| *Ditrichocorycaeus anglicus* | 0.00 |  | 0.00 | 0.00 |  | 0.00 | 1.89 |
| *Ebalia cranchii* | 0.55 |  | 0.00 | 0.00 |  | 0.00 | 0.47 |
| *Echiodon drummondii* | 0.00 |  | 0.00 | 0.00 |  | 0.00 | 0.00 |
| *Embletonia pulchra* | 0.00 |  | 0.61 | 0.00 |  | 0.00 | 0.00 |
| *Euchaeta* sp. | 0.00 |  | 0.00 | 0.00 |  | 0.00 | 0.00 |
| *Euphausia krohni* | 0.00 |  | 0.00 | 0.00 |  | 0.00 | 3.77 |
| *Euphysa aurata* | 0.00 |  | 0.00 | 1.85 |  | 0.24 | 0.94 |
| *Eurynome spinosa* | 0.00 |  | 0.00 | 0.00 |  | 0.00 | 0.47 |
| *Eusergestes arcticus* | 0.00 |  | 0.00 | 0.00 |  | 0.00 | 0.00 |
| *Euspira nitida* | 0.00 |  | 0.00 | 0.00 |  | 0.24 | 0.00 |
| *Euterpina acutifrons* | 0.00 |  | 0.00 | 0.00 |  | 0.00 | 0.00 |
| *Evadne nordmanni* | 1.64 |  | 1.22 | 0.00 |  | 2.12 | 0.94 |
| Gastropoda cl. | 0.00 |  | 0.00 | 0.37 |  | 0.24 | 0.00 |
| *Goneplax rhomboides* | 3.83 |  | 0.61 | 0.74 |  | 0.00 | 0.00 |
| *Gracilechinus acutus* | 0.00 |  | 0.00 | 0.00 |  | 0.00 | 0.00 |
| *Gymnammodytes cicerelus* | 0.00 |  | 0.00 | 0.74 |  | 0.00 | 0.00 |
| *Helgicirrha cari* | 0.00 |  | 0.00 | 0.74 |  | 0.00 | 0.00 |
| *Illex coindetii* | 0.00 |  | 0.00 | 0.37 |  | 0.00 | 0.00 |
| *Inachus dorsettensis* | 0.00 |  | 0.00 | 0.00 |  | 0.00 | 0.47 |
| *Inachus communissimus* | 0.00 |  | 0.00 | 0.00 |  | 0.00 | 0.00 |
| *Jaxea nocturna* | 2.19 |  | 0.00 | 1.48 |  | 0.00 | 0.47 |
| *Labioleanira yhleni* | 0.00 |  | 0.00 | 0.00 |  | 0.00 | 0.00 |
| *Lesueurigobius friesii* | 0.00 |  | 0.00 | 0.37 |  | 0.00 | 0.00 |
| *Leuckartiara octona* | 0.00 |  | 0.00 | 1.11 |  | 0.71 | 0.00 |
| *Limacina inflata* | 0.00 |  | 0.00 | 0.00 |  | 0.00 | 0.00 |
| *Liocarcinus depurator* | 0.00 |  | 0.00 | 0.37 |  | 0.00 | 0.47 |
| *Liocarcinus maculatus* | 0.55 |  | 1.22 | 0.00 |  | 0.00 | 0.00 |
| *Liocarcinus vernalis* | 0.55 |  | 4.27 | 0.74 |  | 0.24 | 0.00 |
| *Lizzia blondina* | 2.19 |  | 0.61 | 2.21 |  | 3.54 | 1.89 |
| *Longipedia* sp. | 1.09 |  | 1.83 | 0.00 |  | 1.89 | 0.47 |
| *Lucicutia flavicornis* | 0.00 |  | 0.00 | 1.85 |  | 0.71 | 4.25 |
| *Luidia sarsi* | 0.00 |  | 0.00 | 0.00 |  | 0.00 | 0.00 |
| *Magelona* sp. | 4.37 |  | 2.44 | 1.48 |  | 3.30 | 0.94 |
| *Mecynocera clausi* | 0.00 |  | 0.00 | 0.37 |  | 2.83 | 0.47 |
| *Meganyctiphanes norvegica* | 0.00 |  | 0.00 | 0.00 |  | 0.00 | 0.00 |
| *Merluccius merluccius* | 0.00 |  | 0.00 | 0.37 |  | 0.00 | 0.00 |
| *Mesaiokeras hurei* | 0.00 |  | 0.00 | 0.37 |  | 0.00 | 0.00 |
| *Mesocalanus tenuicornis* | 0.55 |  | 0.00 | 2.21 |  | 0.47 | 0.00 |
| *Microsetella norvegica* | 0.00 |  | 0.00 | 1.85 |  | 2.36 | 2.83 |
| *Muggiaea atlantica* | 0.00 |  | 0.00 | 2.58 |  | 3.54 | 1.89 |
| *Mullus barbatus* | 0.55 |  | 0.61 | 0.37 |  | 0.00 | 0.47 |
| *Nannocalanus minor* | 0.00 |  | 0.00 | 0.00 |  | 1.89 | 0.94 |
| *Nanomia bijuga* | 0.55 |  | 6.10 | 4.43 |  | 3.07 | 1.42 |
| *Nematoscelis megalops* | 0.00 |  | 0.00 | 0.00 |  | 0.00 | 2.36 |
| *Obelia dichotoma* | 0.00 |  | 0.61 | 0.37 |  | 3.07 | 2.36 |
| *Oncaea mediterranea* | 0.00 |  | 0.00 | 0.00 |  | 0.00 | 0.00 |
| *Oncaea scottodicarloi* | 0.00 |  | 0.00 | 0.00 |  | 0.24 | 0.47 |
| *Oncaea venusta* | 3.83 |  | 3.66 | 0.37 |  | 1.42 | 0.94 |
| *Ophiocomina nigra* | 0.55 |  | 0.00 | 0.00 |  | 0.24 | 0.00 |
| *Ophiura grubei* | 4.92 |  | 1.22 | 0.37 |  | 0.47 | 0.00 |
| *Ophiura ophiura* | 0.00 |  | 0.00 | 0.37 |  | 0.00 | 0.00 |
| *Pagurus prideaux* | 0.00 |  | 0.00 | 0.00 |  | 0.24 | 0.00 |
| *Pandalina brevirostris* | 0.00 |  | 0.00 | 0.74 |  | 0.47 | 0.94 |
| *Paracalanus parvus* | 4.92 |  | 7.32 | 4.80 |  | 3.30 | 3.30 |
| *Paracalanus spp.* | 4.92 |  | 7.32 | 1.85 |  | 2.83 | 0.94 |
| *Paracentrotus lividus* | 1.09 |  | 0.00 | 2.21 |  | 0.47 | 0.47 |
| *Pasiphaea sivado* | 0.00 |  | 0.00 | 0.00 |  | 0.24 | 0.00 |
| *Pectinaria koreni* | 0.00 |  | 0.00 | 0.00 |  | 0.24 | 0.00 |
| *Penilia avirostris* | 3.83 |  | 0.00 | 0.37 |  | 0.00 | 0.00 |
| *Philocheras bispinosus* | 0.55 |  | 0.61 | 1.48 |  | 1.65 | 0.47 |
| *Phrosina semilunata* | 0.00 |  | 0.00 | 0.00 |  | 0.00 | 0.47 |
| *Phyllodoce rosea* | 0.55 |  | 0.00 | 0.37 |  | 0.24 | 0.00 |
| Phyllodocida ord. | 0.00 |  | 0.00 | 0.00 |  | 0.00 | 0.00 |
| *Pleopis polyphemoides* | 4.92 |  | 5.49 | 0.74 |  | 3.07 | 0.00 |
| *Pleuromamma borealis* | 0.00 |  | 0.00 | 0.37 |  | 0.00 | 2.36 |
| *Pleuromamma gracilis* | 0.00 |  | 0.00 | 1.11 |  | 0.24 | 2.36 |
| *Podocoryna areolata* | 0.00 |  | 0.00 | 0.37 |  | 0.71 | 0.00 |
| *Podon intermedius* | 3.28 |  | 0.00 | 2.21 |  | 2.83 | 0.94 |
| Poecilostomatoida ord. | 0.00 |  | 0.00 | 0.00 |  | 0.00 | 0.47 |
| *Pomatoschistus pictus* | 0.00 |  | 0.00 | 0.00 |  | 0.00 | 0.00 |
| Porifera ph. | 0.00 |  | 0.00 | 0.00 |  | 1.42 | 1.42 |
| *Porroecia spinirostris* | 0.00 |  | 0.00 | 0.00 |  | 0.00 | 0.47 |
| *Proceroecia microprocera* | 0.00 |  | 0.00 | 0.00 |  | 0.00 | 0.00 |
| *Processa edulis* | 0.00 |  | 0.00 | 0.00 |  | 0.47 | 0.00 |
| *Processa modica* | 0.55 |  | 1.83 | 0.00 |  | 0.47 | 0.00 |
| *Processa nouveli* | 2.19 |  | 1.22 | 1.11 |  | 2.12 | 1.89 |
| *Pseudameira* sp. | 0.00 |  | 0.00 | 0.00 |  | 0.24 | 0.00 |
| *Pseudocuma similis* | 0.00 |  | 0.00 | 0.00 |  | 0.00 | 0.00 |
| *Pseudosagitta lyra* | 0.00 |  | 0.00 | 0.00 |  | 0.00 | 0.00 |
| *Pusillina inconspicua* | 0.00 |  | 0.00 | 0.00 |  | 0.00 | 0.00 |
| *Rhincalanus nasutus* | 0.00 |  | 0.00 | 0.00 |  | 0.24 | 1.42 |
| *Rissoides desmaresti* | 0.55 |  | 0.00 | 0.00 |  | 0.00 | 0.00 |
| *Salmo salar* | 0.00 |  | 0.00 | 0.00 |  | 0.24 | 0.00 |
| *Sciaena umbra* | 0.55 |  | 0.00 | 0.00 |  | 0.24 | 0.00 |
| *Scolaricia* sp. | 0.00 |  | 0.00 | 0.37 |  | 0.00 | 0.00 |
| *Scolecithricella dentata* | 0.00 |  | 0.00 | 0.00 |  | 0.00 | 0.47 |
| *Sepia officinalis* | 0.00 |  | 0.00 | 0.37 |  | 0.00 | 0.00 |
| *Solenocera membranacea* | 2.73 |  | 0.61 | 2.58 |  | 2.59 | 0.94 |
| *Sphaeronectes gracilis* | 0.00 |  | 0.00 | 0.00 |  | 0.94 | 2.36 |
| *Spicara maena* | 0.00 |  | 0.00 | 0.37 |  | 0.24 | 0.47 |
| *Spisula subtruncata* | 0.55 |  | 0.61 | 0.74 |  | 0.71 | 0.94 |
| *Sprattus sprattus* | 3.83 |  | 0.00 | 0.00 |  | 0.00 | 0.00 |
| *Stauridiosarsia gemmifera* | 2.19 |  | 2.44 | 0.74 |  | 0.00 | 0.00 |
| Stomiiformes ord. | 0.00 |  | 0.00 | 0.00 |  | 0.00 | 0.00 |
| *Subeucalanus pileatus* | 0.00 |  | 0.00 | 0.00 |  | 0.24 | 0.00 |
| Terebellida ord. | 0.00 |  | 0.61 | 0.00 |  | 0.00 | 0.00 |
| *Triconia dentipes* | 0.00 |  | 0.00 | 0.00 |  | 1.65 | 0.47 |
| *Trisopterus capelanus* | 2.73 |  | 0.00 | 0.00 |  | 0.24 | 0.47 |
| Tubulanidae fam. | 0.00 |  | 0.61 | 2.21 |  | 2.12 | 0.00 |
| Unidentified decapod | 0.00 |  | 0.00 | 0.00 |  | 0.00 | 0.00 |
| *Vibilia armata* | 0.00 |  | 0.00 | 0.37 |  | 0.00 | 0.47 |

**Table S06.** Diet composition applying DNA metabarcoding to stomach contents of **(a)** anchovy and **(b)** sardine, presented as frequency of occurrence (%O), by stage (*juv*: juveniles; *ad*: adults) and areas (GSA07, GSA06-North, GSA06-South). Number of samples (N) for each category is presented in Table 6.

**a) anchovy**

|  | GSA07 |  | GSA06-North | |  | GSA06-South | |
| --- | --- | --- | --- | --- | --- | --- | --- |
| Prey group (species)⇓ stage⇒ | juv |  | juv | ad |  | juv | ad |
| *Amphora helenensis* | 0.93 |  | 0.00 | 0.00 |  | 0.00 | 0.00 |
| *Arcocellulus mammifer* | 0.00 |  | 4.55 | 4.29 |  | 1.69 | 1.27 |
| *Asterionellopsis guyunusae* | 2.80 |  | 3.64 | 0.86 |  | 0.00 | 0.64 |
| Bacillariophyta ph. | 3.74 |  | 6.36 | 5.58 |  | 6.78 | 10.19 |
| *Cerataulina pelagica* | 3.74 |  | 8.18 | 3.00 |  | 5.08 | 3.82 |
| *Chaetoceros didymus* | 3.74 |  | 0.00 | 1.29 |  | 0.00 | 0.00 |
| *Chaetoceros socialis* | 8.41 |  | 0.00 | 4.72 |  | 5.08 | 3.82 |
| *Chaetoceros danicus* | 2.80 |  | 3.64 | 2.15 |  | 0.00 | 0.64 |
| *Corethron hystrix* | 0.00 |  | 0.00 | 0.00 |  | 0.00 | 0.00 |
| *Coscinodiscus wailesii* | 1.87 |  | 0.91 | 0.43 |  | 0.00 | 0.64 |
| *Cylindrotheca closterium* | 1.87 |  | 0.00 | 2.15 |  | 1.69 | 0.00 |
| *Eucampia cornuta* | 4.67 |  | 0.00 | 3.00 |  | 5.08 | 1.27 |
| *Guinardia striata* | 4.67 |  | 1.82 | 2.15 |  | 3.39 | 1.91 |
| *Hemiaulus sinensis* | 0.93 |  | 0.91 | 0.86 |  | 1.69 | 0.64 |
| *Lauderia annulata* | 3.74 |  | 0.00 | 1.29 |  | 0.00 | 0.00 |
| *Leptocylindrus danicus* | 3.74 |  | 2.73 | 3.43 |  | 5.08 | 7.64 |
| *Licmophora abbreviata* | 0.00 |  | 0.00 | 0.00 |  | 1.69 | 0.00 |
| *Melosira varians* | 0.00 |  | 5.45 | 0.43 |  | 3.39 | 2.55 |
| *Minidiscus trioculatus* | 2.80 |  | 10.91 | 7.30 |  | 8.47 | 12.10 |
| *Nitzschia* sp. | 0.00 |  | 1.82 | 0.43 |  | 1.69 | 2.55 |
| *Odontella mobiliensis* | 0.00 |  | 0.00 | 0.00 |  | 0.00 | 0.00 |
| *Papiliocellulus simplex* | 6.54 |  | 7.27 | 5.15 |  | 1.69 | 3.82 |
| *Paralia sulcata* | 0.00 |  | 0.91 | 0.00 |  | 0.00 | 0.64 |
| *Planktoniella sol* | 0.93 |  | 0.00 | 2.15 |  | 1.69 | 1.91 |
| *Psammodictyon constrictum* | 0.00 |  | 4.55 | 2.58 |  | 3.39 | 0.00 |
| *Pseudo-nitzschia americana* | 1.87 |  | 0.00 | 2.58 |  | 3.39 | 2.55 |
| *Pseudo-nitzschia delicatissima* | 8.41 |  | 9.09 | 6.44 |  | 6.78 | 8.92 |
| *Pseudo-nitzschia galaxiae* | 3.74 |  | 6.36 | 5.15 |  | 8.47 | 7.01 |
| *Pseudo-nitzschia multiseries* | 0.00 |  | 0.00 | 1.72 |  | 3.39 | 0.00 |
| *Pseudo-nitzschia multistriata* | 0.00 |  | 0.00 | 1.72 |  | 0.00 | 0.64 |
| *Pseudo-nitzschia* sp. | 3.74 |  | 0.91 | 2.15 |  | 1.69 | 4.46 |
| *Pseudo-nitzschia calliantha* | 5.61 |  | 0.91 | 2.58 |  | 1.69 | 0.00 |
| *Pseudosolenia calcar-avis* | 6.54 |  | 0.00 | 3.00 |  | 3.39 | 0.64 |
| *Rhizosolenia fallax* | 5.61 |  | 7.27 | 4.29 |  | 5.08 | 7.01 |
| *Rhizosolenia shrubsolei* | 5.61 |  | 7.27 | 5.58 |  | 3.39 | 5.73 |
| *Skeletonema potamos* | 0.00 |  | 0.00 | 2.58 |  | 0.00 | 0.00 |
| *Thalassionema frauenfeldii* | 0.00 |  | 0.91 | 1.72 |  | 0.00 | 0.00 |
| *Thalassiosira mediterranea* | 0.93 |  | 2.73 | 3.86 |  | 3.39 | 3.82 |
| *Thalassiosira oceanica* | 0.00 |  | 0.91 | 2.58 |  | 1.69 | 3.18 |
| *Thalassiosira oestrupii* | 0.00 |  | 0.00 | 0.00 |  | 0.00 | 0.00 |
| *Thalassiosira pseudonana* | 0.00 |  | 0.00 | 0.86 |  | 0.00 | 0.00 |

**b) sardine**

|  | GSA07 |  | GSA06-North | |  | GSA06-South | |
| --- | --- | --- | --- | --- | --- | --- | --- |
| Prey group (species)⇓ stage⇒ | juv |  | juv | ad |  | juv | ad |
| *Amphora helenensis* | 0.00 |  | 0.00 | 0.00 |  | 0.88 | 1.36 |
| *Arcocellulus mammifer* | 0.40 |  | 0.00 | 1.98 |  | 0.00 | 2.72 |
| *Asterionellopsis guyunusae* | 4.05 |  | 0.00 | 2.78 |  | 0.00 | 0.00 |
| Bacillariophyta ph. | 4.05 |  | 3.75 | 4.37 |  | 3.53 | 6.80 |
| *Cerataulina pelagica* | 4.05 |  | 12.50 | 5.16 |  | 4.41 | 4.76 |
| *Chaetoceros didymus* | 4.05 |  | 2.50 | 2.38 |  | 1.76 | 0.68 |
| *Chaetoceros socialis* | 4.05 |  | 1.25 | 3.17 |  | 4.41 | 6.12 |
| *Chaetoceros danicus* | 4.05 |  | 5.00 | 3.17 |  | 2.35 | 3.40 |
| *Corethron hystrix* | 0.00 |  | 0.00 | 0.79 |  | 0.00 | 0.68 |
| *Coscinodiscus wailesii* | 4.05 |  | 1.25 | 0.40 |  | 3.24 | 0.00 |
| *Cylindrotheca closterium* | 4.05 |  | 5.00 | 2.78 |  | 2.94 | 1.36 |
| *Eucampia cornuta* | 0.81 |  | 0.00 | 1.98 |  | 4.12 | 2.04 |
| *Guinardia striata* | 4.05 |  | 0.00 | 2.38 |  | 2.94 | 1.36 |
| *Hemiaulus sinensis* | 3.24 |  | 0.00 | 3.17 |  | 2.65 | 2.04 |
| *Lauderia annulata* | 2.83 |  | 0.00 | 1.98 |  | 2.65 | 2.72 |
| *Leptocylindrus danicus* | 4.05 |  | 5.00 | 3.17 |  | 4.41 | 4.08 |
| *Licmophora abbreviata* | 0.00 |  | 0.00 | 0.00 |  | 0.00 | 0.68 |
| *Melosira varians* | 0.00 |  | 3.75 | 0.00 |  | 0.00 | 0.00 |
| *Minidiscus trioculatus* | 2.43 |  | 5.00 | 3.57 |  | 4.41 | 10.20 |
| *Nitzschia* sp. | 0.40 |  | 0.00 | 0.40 |  | 3.82 | 3.40 |
| *Odontella mobiliensis* | 2.43 |  | 0.00 | 1.98 |  | 0.59 | 0.68 |
| *Papiliocellulus simplex* | 4.05 |  | 8.75 | 4.37 |  | 4.41 | 3.40 |
| *Paralia sulcata* | 0.81 |  | 0.00 | 0.40 |  | 0.00 | 0.00 |
| *Planktoniella sol* | 0.00 |  | 0.00 | 0.40 |  | 1.76 | 0.68 |
| *Psammodictyon constrictum* | 1.21 |  | 3.75 | 1.19 |  | 2.35 | 1.36 |
| *Pseudo-nitzschia americana* | 4.05 |  | 0.00 | 3.17 |  | 4.41 | 2.04 |
| *Pseudo-nitzschia delicatissima* | 4.05 |  | 12.50 | 4.76 |  | 4.41 | 5.44 |
| *Pseudo-nitzschia galaxiae* | 4.05 |  | 0.00 | 3.17 |  | 4.41 | 4.76 |
| *Pseudo-nitzschia multiseries* | 3.24 |  | 0.00 | 2.38 |  | 3.82 | 2.72 |
| *Pseudo-nitzschia multistriata* | 0.00 |  | 0.00 | 1.98 |  | 1.76 | 1.36 |
| *Pseudo-nitzschia* sp. | 4.05 |  | 0.00 | 3.57 |  | 2.35 | 2.04 |
| *Pseudo-nitzschia calliantha* | 4.05 |  | 1.25 | 4.37 |  | 3.53 | 1.36 |
| *Pseudosolenia calcar-avis* | 4.05 |  | 0.00 | 2.38 |  | 3.82 | 2.04 |
| *Rhizosolenia fallax* | 4.05 |  | 15.00 | 5.56 |  | 4.41 | 5.44 |
| *Rhizosolenia shrubsolei* | 4.05 |  | 13.75 | 5.16 |  | 4.41 | 6.80 |
| *Skeletonema potamos* | 0.00 |  | 0.00 | 2.38 |  | 0.88 | 0.68 |
| *Thalassionema frauenfeldii* | 4.05 |  | 0.00 | 0.79 |  | 0.00 | 0.00 |
| *Thalassiosira mediterranea* | 0.00 |  | 0.00 | 2.38 |  | 2.06 | 1.36 |
| *Thalassiosira oceanica* | 0.40 |  | 0.00 | 3.17 |  | 1.76 | 2.72 |
| *Thalassiosira oestrupii* | 0.81 |  | 0.00 | 1.19 |  | 0.29 | 0.68 |
| *Thalassiosira pseudonana* | 0.00 |  | 0.00 | 1.59 |  | 0.00 | 0.00 |

**Table S07.** Diatoms detected in gut contents applying DNA metabarcoding in **(a)** anchovy and **(b)** sardine, presented as frequency of occurrence (%O), by stage (*juv*: juveniles; *ad*: adults) and areas (GSA07, GSA06-North, GSA06-South). Number of samples (N) for each category is presented in Table 6.

| **Species** | **Area** | **Stage** | ***N*** | **Mean *δ*^13^C ± S*D*** | **Mean *δ*^15^N ± *SD*** |
| --- | --- | --- | --- | --- | --- |
| Anchovy | GSA07 | juv | 26 | -19.75 ±0.34 | 7.13±0.32 |
|  |  | ad | 13 | -19.66 ±0.47 | 7.00±0.53 |
|  |  | tot | 39 | -19.72 ±0.38 | 7.09±0.40 |
|  | GSA06-North | juv | 10 | -19.60 ±0.30 | 8.02±0.59 |
|  |  | ad | 14 | -19.16 ±0.28 | 8.38±0.38 |
|  |  | tot | 24 | -19.34 ±0.36 | 8.23±0.50 |
|  | GSA06-South | juv | 6 | -18.97 ±0.59 | 8.30±0.33 |
|  |  | ad | 16 | -18.85 ±0.30 | 8.11±0.24 |
|  |  | tot | 22 | -18.88 ±0.39 | 8.17±0.27 |
| Sardine | GSA07 | juv | 38 | -19.33 ±0.70 | 7.75±0.34 |
|  | GSA06-North | juv | 12 | -19.94 ±0.77 | 8.74±0.17 |
|  |  | ad | 12 | -19.32 ±0.78 | 8.82±0.25 |
|  |  | tot | 24 | -19.63 ±0.82 | 8.78±0.21 |
|  | GSA06-South | juv | 10 | -19.18 ±0.20 | 7.73±0.17 |
|  |  | ad | 15 | -18.99 ±0.56 | 8.49±0.22 |
|  |  | tot | 25 | -19.06 ±0.46 | 8.18±0.43 |

**Table S08.** Mean *δ*^13^C and *δ*^15^N values (and standard deviations, *SD*) obtained for *N* number of anchovy (*Engraulis encrasicolus*) and sardine (*Sardina pilchardus*) samples, analysed per area and stage (*juv*: juveniles; *ad*: adults; *tot*: total).

**a**

| ***δ*^15^N** |  | **D^2^ %** | **AIC** | | |
| --- | --- | --- | --- | --- | --- |
| anchovy | |  |  | | |
| **Model 1** | **1*+ Lat*** | **70.6** | **79.65** | | |
| Model 2 | 1*+ TL +L* + D + A | 73.0 | 83.10 | | |
| Model 3 | 1*+ Lat * + D + A | 72.4 | 82.08 | | |
| Model 4 | 1*+ Lat * + D | 72.1 | 81.21 | | |
| Model 5 | 1*+D* | 54.6 | 118.75 | | |
| sardine | |  | |  |  |
| **Model 1** | **1*+ TL*+ A *** | **67.4** | **52.36** | | |
| Model 2 | 1*+ TL*+Lat + D + A* | 71.9 | 46.88 | | |
| Model 3 | 1*+ TL*+Lat + A* | 71.5 | 46.11 | | |
| Model 4 | 1*+ TL | 22.9 | 125.94 | | |
| Model 5 | 1*+ A | 61.7 | 64.46 | | |

**b**

| ***δ*^13^C** |  | **D^2^ %** | **AIC** | | |
| --- | --- | --- | --- | --- | --- |
| **anchovy** | |  |  | | |
| **Model 1** | **1*+ Lat* + TL*+ D*** | **53.2** | **71.57** | | |
| Model 2 | 1*+ TL* + Lat + D* + A | 53.3 | 75.26 | | |
| Model 3 | 1*+ Lat * + D* + TL* | 53.2 | 76.52 | | |
| Model 4 | 1*+ Lat * | 47.9 | 94.41 | | |
| Model 5 | 1*+D* | 16.1 | 73.56 | | |
| **sardine** | |  | |  |  |
| **Model 1** | **1*+ D*** | **28.0** | **167.44** | | |
| Model 2 | 1*+ TL +Lat + D* + A | 30.9 | 172.14 | | |
| Model 3 | 1*+ D* + Lat + A | 29.4 | 179.56 | | |
| Model 4 | 1*+ D* + Lat | 29.0 | 168.31 | | |
| Model 5 | 1*+ Lat* | 9.51 | 169.17 | | |

**c**

|  |  | **D^2^ %** | **AIC** | | |
| --- | --- | --- | --- | --- | --- |
| **anchovy** | |  |  | | |
| **Model 1** | **1*+Lat*+TL*+Lat:TL*** | **50.6** | **97.31** | | |
| Model 2 | 1*+Lat+TL | 19.7 | 122.88 | | |
| Model 3 | 1*+Lat+TL*+D* | 30.2 | 113.87 | | |
| Model 4 | 1*+D*+Lat | 23.0 | 116.25 | | |
| Model 5 | 1*+D*+TL* | 30.1 | 112.08 | | |
| **sardine** | |  | |  |  |
| **Model 1** | **1*+L*+TL*+L:TL*** | **66.3** | **70.03** | | |
| Model 2 | 1*+D+L*+TL | 36.4 | 92.37 | | |
| Model 3 | 1*+D+TL*+L*+A* | 58.1 | 72.23 | | |
| Model 4 | 1*+ TL+L* | 34.0 | 92.17 | | |
| Model 5 | 1*+D*+TL | 28.5 | 96.70 | | |

**Table S09.** Comparison of the most relevant GAMs tested for **(a)** *δ*^15^N, **(b)** for *δ*^13^C, and **(c)** for the Shannon diversity index (*H*’) of anchovy and sardine (the corresponding numerical summaries are presented in Table 2). Explanatory variable acronyms are: D = depth (m), Lat = latitude, TL = total length of fish (cm), A = area. Statistics acronyms are: AIC = Akaike Information Criterion, D^2^ = deviance explained. Significant predictors are indicated with ‘*’. The best *δ*^15^N GAM model is highlighted in bold.

|  |  |  | GSA07 | |  | GSA06-North | |  | GSA06-South | |
| --- | --- | --- | --- | --- | --- | --- | --- | --- | --- | --- |
|  |  |  | *E.enc* | *S.pil* |  | *E.enc* | *S.pil* |  | *E.enc* | *S.pil* |
|  |  |  |  |  |  |  |  |  |  |  |
| GSA07 | *E.enc* |  |  | 3.09  (0.00-13.93) |  | 0.00  (0.00-0.01) |  |  | 0 |  |
|  | *S.pil* |  | 4.39  (0.00-19.61) |  |  |  | 0 |  |  | 28.17  (10.44-44.44) |
|  |  |  |  |  |  |  |  |  |  |  |
| GSA06-North | *E.enc* |  | 0.00  (0.00-0.01) |  |  |  | 2.59  (0.00-22.12) |  | 11.07  (0.61-27.67) |  |
|  | *S.pil* |  |  | 0 |  | 2.42  (0.00-18.63 |  |  |  | 0.00  (0.00-5.86) |
|  |  |  |  |  |  |  |  |  |  |  |
| GSA06-South | *E.enc* |  | 0 |  |  | 17.96  (1.06-46.45) |  |  |  | 27.53  (12.92-39.72) |
|  | *S.pil* |  |  | 30.16  (10.53-47.81) |  |  | 0.00  (0.00-4.90) |  | 54.80  (25.72-79.31) |  |

**Table S10.** Paired comparisons of the median isotopic standard ellipses area overlap of anchovy and sardine between sampling areas. The SEA_B_ overlap should be read as the percentage of the sampling area of the rows versus the sampling area of the columns. 25% and 75% percentiles are given in parenthesis.

**References**

1. Bachiller, E. & Irigoien, X. Allometric relations and consequences for feeding in small pelagic fish in the Bay of Biscay. *ICES J. Mar. Sci.* **70**, 232–243 (2013).

2. Hallegraeff, G. *Manual on harmful marine microalgae*. *Monogr. Oceanogr. Methodol* **11**, (UNESCO Publishing, 2003).

3. Vila, M. & Masó, M. Phytoplankton functional groups and harmful algal species in anthropogenically impacted waters of the NW Mediterranean Sea. *Sci. Mar.* **69**, 31–45 (2005).

4. Smayda, T. J. *Harmful algal bloom communities in Scottish coastal water: Relationship to fish farming and regional comparisons - A review*. (2006).

5. Lassus, P., Chomerat, N., Hess, P. & Nezan, E. *Toxic and harmful microalgae of the World Ocean*. (International Society for the Study of Harmful Algae / Intergovernmental Oceanographic Commission of UNESCO, 2016).

6. Percopo, I., Siano, R., Cerino, F., Sarno, D. & Zingone, A. Phytoplankton diversity during the spring bloom in the northwestern Mediterranean Sea. *Bot. Mar.* **54**, 243–267 (2011).
